# Programmed Iteration Controls the Assembly of the Nonanoic Acid Side Chain of the Antibiotic Mupirocin

**DOI:** 10.1002/anie.202212393

**Published:** 2022-11-10

**Authors:** Ashley J. Winter, Matthew T. Rowe, Angus N. M. Weir, Nahida Akter, Sbusisiwe Z. Mbatha, Paul D. Walker, Christopher Williams, Zhongshu Song, Paul R. Race, Christine L. Willis, Matthew P. Crump

**Affiliations:** ^1^ School of Chemistry University of Bristol Bristol BS8 1TS UK; ^2^ School of Biochemistry University of Bristol Bristol BS8 1TD UK

**Keywords:** Fatty Acids, Mupirocin, Natural Product Biosynthesis, Polyketide Antibiotics, Pseudomonic Acid

## Abstract

Mupirocin is a clinically important antibiotic produced by *Pseudomonas fluorescens* NCIMB 10586 that is assembled by a complex *trans*‐AT polyketide synthase. The polyketide fragment, monic acid, is esterified by a 9‐hydroxynonanoic acid (9HN) side chain which is essential for biological activity. The ester side chain assembly is initialised from a 3‐hydroxypropionate (3HP) starter unit attached to the acyl carrier protein (ACP) MacpD, but the fate of this species is unknown. Herein we report the application of NMR spectroscopy, mass spectrometry, chemical probes and in vitro assays to establish the remaining steps of 9HN biosynthesis. These investigations reveal a complex interplay between a novel iterative or “stuttering” KS‐AT didomain (MmpF), the multidomain module MmpB and multiple ACPs. This work has important implications for understanding the late‐stage biosynthetic steps of mupirocin and will be important for future engineering of related *trans*‐AT biosynthetic pathways (e.g. thiomarinol).

## Graphical abstract

Mupirocin is a clinically important antibiotic in which the polyketide fragment, monic acid, is esterified to a 9‐hydroxynonanoic acid (9HN) side chain, which is essential for biological activity. NMR spectroscopy, mass spectrometry, chemical probes and in vitro assays were used to establish how 9HN is assembled through the action of multiple acyl carrier proteins, two ketosynthases and fatty acid β‐keto processing enzymes.

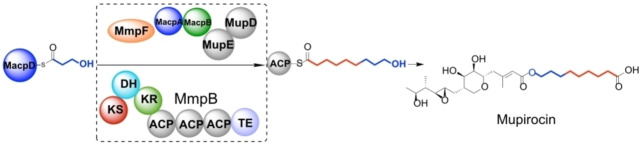

## Introduction

**Figure 1:**
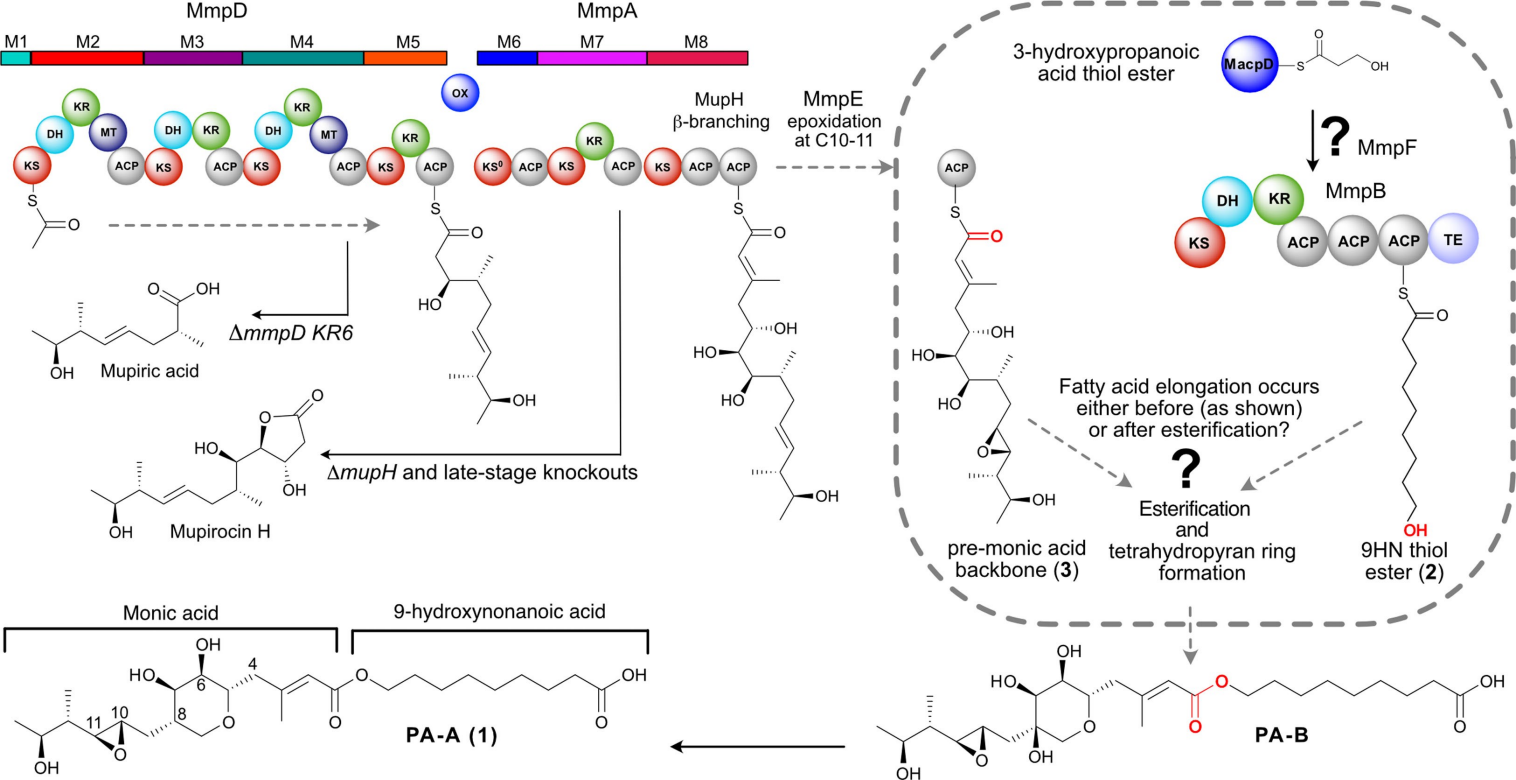
Mupirocin biosynthesis. The C_17_ monic acid backbone is assembled by MmpD, MmpA and MmpE. Esterification of the C_17_ pre‐monic acid backbone (**3**) with the fatty acid chain (shown is the full length 9‐hydroxynonanoic acid thiol ester (**2**)) and tetrahydropyran ring (THP) formation yields PA‐B. PA‐B is subsequently converted to PA‐A (**1**) in a further multistep cascade. Accumulation of both mupiric acid and mupirocin H is observed when specific mutations in *mmpD* or *mmpA* are made and importantly also when late‐stage enzymes are inactivated. ACP—acyl carrier protein, AT—acyl transferase, ER—enoylreductase, DH—dehydratase, KR—ketoreductase, KS—ketosynthase, MT, methyl transferase, Ox—oxidoreductase, TE—thioesterase.

Mupirocin is a polyketide antibiotic first isolated from *Pseudomonas fluorescens* NCIMB 10585 that has specific activity against methicillin resistant *Staphylococcus aureus* (MRSA).^[1]^ Mupirocin is marketed under the name of Bactroban^TM^ and is used predominantly for the topical treatment of bacterial skin infections and as a protective agent.^[2]^ Mupirocin exists as a mixture of pseudomonic acids (PA), whereby the main active component PA‐A (**1**) accounts for 90 % of the mixture (Figure 1).[^1^, ^3^] The molecular framework consists of a 9‐hydroxynonanoic acid (9HN) esterified to a C_17_ monic acid polyketide containing a tetrahydropyran (THP) core. Mupirocin is produced by a hybrid *trans*‐acyltransferase (*trans*‐AT) polyketide synthase (PKS).^[4]^ The *trans*‐AT PKSs are a subclass of the type I non‐iterative synthases which are large protein assemblies comprising multiple domains covalently bound together in modules. Each extension module assembles the polyketide backbone through a decarboxylative Claisen condensation of a malonyl group (or methylmalonyl) that successively incorporates two backbone carbon atoms and is catalysed by ketosynthase (KS) domains. Each module contains an acyl carrier protein (ACP) bearing a phosphopantetheine sidearm that is primed with the acyl extender unit by a *trans*‐acting AT.^[5]^ In contrast, the *cis*‐AT PKSs have distinct acyl transferase domains covalently bound within each respective extension domain.^[6]^ These act once, loading an acyl group onto a specific adjacent ACP. A hallmark of the *cis*‐AT PKSs is broad adherence to co‐linearity, whereby each module incorporates one unit, and a processive mechanism, where each catalytic step has a single associated enzyme domain. In these cases, the polyketide product is straightforwardly predicted by analysis of the PKS gene cluster and the sequence of encoded catalytic domains, although increasingly there are exceptions.^[7]^ Conversely, *trans*‐AT PKSs often comprise a series of multifunctional proteins arranged in a nonlinear fashion, supplemented with an array of *trans*‐acting partners (in addition to the AT) that can selectively functionalise an intermediate. This generates greater chemical diversity and makes these pathways attractive, but challenging targets for mechanistic investigation.

Elucidation of the mupirocin biosynthetic pathway has arguably presented one of the greatest biosynthetic challenges. The gene cluster totaling 74 kb consists of six large open reading frames (ORFs), termed mupirocin multifunctional proteins (*mmpA* to *mmpF*) and 30 ORFs (*mupA‐Z* and five *trans*‐acting ACPs, *macpA‐E*), many of which had unassigned function or apparent overabundance (eg ACPs and free‐standing KS like domains).^[8]^ Much of the pathway has resisted traditional genetic interrogation, with key late‐stage biosynthetic knock‐outs and mutations yielding uninformative early stage intermediates such as mupiric acid and mupirocin H (Figure 1).^[9]^ Recently several of the critical nonlinear processing steps have, however, been elucidated using a combination of in vitro and in vivo approaches, including THP formation,^[10]^ β‐branch formation,^[11]^ epoxidation of the 10,11‐alkene,^[12]^ conversion of PA‐B into PA‐A by removal of the 8‐OH group^[13]^ and the generation of the unusual 3‐hydroxyproprionate (3HP) fatty acid starter unit from malonyl‐CoA catalysed by MupQ, MupS and MacpD.^[14]^

Despite this progress, many questions remain, including notably how the 9HN fatty acid side chain is assembled from the 3HP precursor. Typically, fatty acid biosynthesis proceeds through recursive 2‐carbon extensions (KS) and reductive β‐keto processing using ketoreductase (KR), dehydratase (DH) and enoyl reductase (ER) catalytic domains. 9HN production from 3HP requires three complete rounds of decarboxylative Claisen condensation (DCC) and reduction, but a single contiguous set of these enzymes are not encoded within any ORF in the biosynthetic pathway. The formation of 9HN has previously been hypothesised to be exclusively biosynthesised in an iterative manner by the modular MmpB, but this has not been proven experimentally (Figure 1). Further this does not account for additional uncharacterised fatty acid synthase (FAS) like domains (eg MmpF (KS‐AT like), MupD and MupE may also play a role.^[8]^ The precise timing of 3HP extension is also unknown and may occur before or after esterification with a pre‐monic acid thiol ester (**3**) (Figure 1 depicts this happening prior to esterification).^[12]^ Gene knock‐outs of the mupirocin and related thiomarinol biosynthetic pathways produce a variety of minor metabolites containing different fatty acid chain lengths on fully or partially processed polyketide scaffolds but the underlying mechanisms are not well understood.^[15]^

In this study we report the elucidation of the steps from 3HP to 9HN in mupirocin biosynthesis. As several ketosynthase domains may be responsible for the three extension rounds and multiple KS‐like domains and ACPs have unassigned function, we applied an in vitro approach using purified protein components and NMR interaction studies, synthetic chemical probes and mass spectrometry assays. This approach allowed the delineation of the 9HN pathway assembly and the involvement of multiple ACPs, two KS domains and FAS β‐keto processing enzymes.

## Results and Discussion

**Figure 2:**
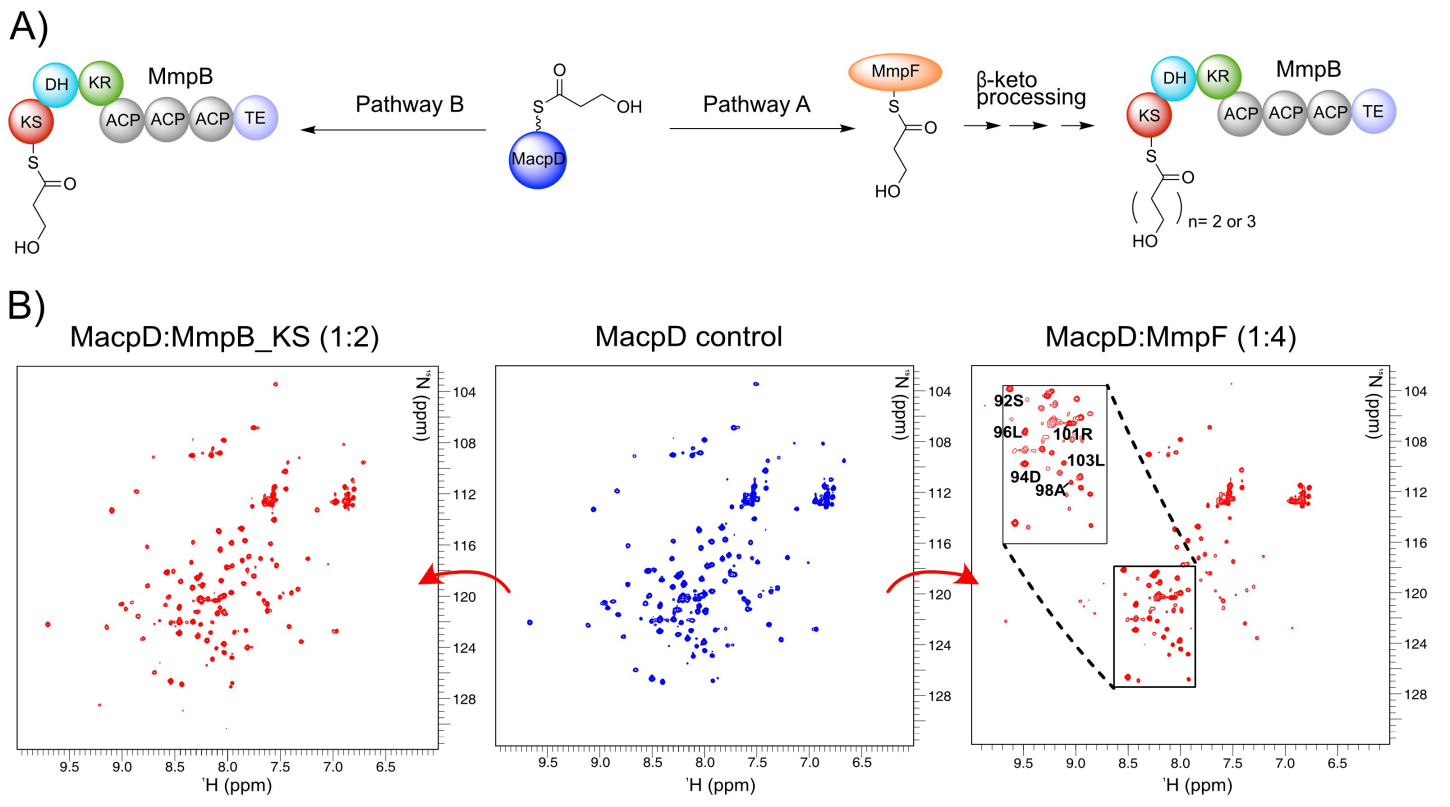
Reaction scheme and ^1^H‐^15^N HSQC titration experiments. A) The possible fate of 3HP‐MacpD. In pathway A, MacpD delivers the 3HP starter unit to MmpF, where chain extension begins. A C_5_ or C_7_ carbon chain is delivered to MmpB for a final round of extension. In pathway B the 3HP unit is delivered directly to MmpB, which catalyses all three rounds of carbon chain extension, and MmpF plays no part. B) ^1^H‐^15^N HSQC spectra of MacpD (centre), MacpD+MmpF (right) and MacpD+MmpB_KS (left).

A significant feature of the mupirocin biosynthetic pathway is an apparent overabundance of enzymes exhibiting KS‐like sequence homology that could represent different paths for how the fatty acid sidechain is assembled (Figure 2A).^[8]^ The modular KS within MmpB (MmpB_KS) and the type 1 mini module MmpF (745 aa) are two such KS type enzymes that have not been functionally characterised. Both contain a canonical β‐ketoacyl synthase “KAS” I like catalytic triad (Cys‐His‐His). A further KS‐like enzyme, MupB, is also unannotated but contains a different catalytic triad (Cys‐His‐Asp) not associated with ketosynthase like activity and may be involved in ester formation (Figure S1).^[16]^

*MmpF* is co‐located immediately downstream of the genes *macpD*, *mupQ and mupS*, responsible for the generation of 3HP and may form an extension of this gene cassette. The encoded MmpF KS (10–506 aa) is fused to a downstream AT‐like domain and C‐terminal loop (507–745 aa) with sequence homology to linker regions adjacent to KS‐AT pairs in Type I PKS synthases.^[17]^ Bioinformatic analysis revealed the AT‐like domain region to be a truncated α/β hydrolase when compared to the intact KS‐AT domain from the mixed *cis*‐AT/NRPS curacin A biosynthetic pathway (Module CurL: KS‐AT‐OMT‐KR‐ACP) and lacking the Ser‐His catalytic dyad (Figure S2). Partial truncations of AT domains have also been observed in *trans* KS domains encoded within other type 1 modules (eg OzmQ_KS1 in the oxazolomycin A pathway and MgsF_KS4 in the iso‐migrastatin pathway) and are predicted to be evolutionary intermediates between the type I *cis*‐AT PKS and the *trans*‐AT type I PKS modules.^[18]^

A bioinformatic study based on MmpB and MmpF KS phylogeny and the known or inferred module functions of 58 characterised *trans‐*AT PKS was performed (Figure S3). MmpF was assigned to a group of KS domains accepting aminoacyl intermediates (Clade XVI) whilst in contrast, MmpB was assigned to a clade predominantly associated with β‐enonyl substrates (Clade IX).^[19]^ MmpF and other members within Clade XVI contain an alanine residue preceding the active site cysteine, which has been associated with the acceptance of bulky substrates, such as the 3HP starter unit. On the other hand, MmpB possesses a methionine, consistent with KS domains that accept linear carbon chains.^[20]^

The mupirocin biosynthetic pathway also contains five discrete ACPs, MacpA‐E, all of which are essential for function. MacpC is involved in the introduction of a methyl β‐branch at C‐3^[11]^ whilst MacpD has been shown to work in tandem with MupQ (an adenylation domain) and MupS (a malonyl‐3‐CoA‐1 ACP reductase) in the formation of the 3HP starter unit.^[14]^ MacpE has been shown to act in late‐stage conversion of PA‐B to PA‐A^[21]^ but MacpA and MacpB remain functionally uncharacterised.^[22]^ Like MacpD, MacpA has a C‐terminal extension that is important for function^[22]^ and MacpA displays high sequence similarity to bacterial (*Rhizobiales*) AcpXL proteins (37–40 %). This class contains an α‐helical C‐terminal extension important for processing 28–30 carbon atom 3‐hydroxylated fatty acids in lipid A biosynthesis (Figure S4). MacpB has previously been shown to share homology with ACPs implicated in polyunsaturated fatty acid biosynthesis.^[22]^

Gene knockouts of *macpA*, *macpB* and *mmpF*_*KS* in *P. fluorescens* were generated and cultured. Purification of metabolites and ESI‐MS analysis confirmed that *ΔmmpF*_*KS, ΔmacpA* and *ΔmacpB* all abolished PA‐A production (Figure S5). Mupirocin H and mupiric acid were detected in extracts from each mutant, albeit in trace amounts for *ΔmacpB*. As previously observed, mutations of post‐PKS assembly genes all produce an essentially identical phenotype in which pseudomonic acid biosynthesis is blocked and the two truncated metabolites, mupiric acid and mupirocin H are isolated (the “leaky hosepipe” mechanism).[^9^, ^23^]

To circumvent these limitations an NMR triage was applied to screen for which KS and ACP domains might form functional pairs using MacpD as the starting point.^[14]^ The genes encoding MmpF, MmpB_KS and MupB proteins were cloned and over‐expressed in *Escherichia coli* and each purified to homogeneity (Figure S6, Table S1). ^15^N MacpD was prepared as previously described.^[14]^ Samples of MmpB_KS, MupB and MmpF were then added to solutions of ^15^N‐*apo* MacpD and monitored by ^1^H‐^15^N HSQC spectra. The ^1^H‐^15^N HSQC of ^15^N‐*apo* MacpD is well resolved and titration experiments with MmpB_KS (Figure 2B left) or MupB (Figure S7) showed no line broadening or observable chemical shift perturbations (CSP). This suggested there was no interaction with MacpD. Conversely, titration of ^15^N‐*apo* MacpD with MmpF revealed striking changes with many resonances significantly line broadened (Figure 2B right). MacpD showed similar interactions with MupS and MupQ, both of which have been shown to be functional partners.^[14]^ It was therefore likely that 3HP‐MacpD would transfer the 3HP starter unit to MmpF in preparation for the first round of decarboxylative Claisen condensation (DCC 1).

To determine the role of the additional ACPs, both MacpA and MacpB, were overexpressed and purified (Figure S8). In addition, each ACP of the MmpB ACP tridomain (MmpB_ACP5‐7) was cloned and expressed as an individual, ^15^N‐labelled domain (MmpB_ACP5, 6 and 7, Figure S7). MacpB failed to isotopically label due to the generation of insoluble protein in minimal media. It was therefore substituted by ^15^N‐labelled TacpB from the homologous thiomarinol biosynthetic pathway where ACP components have been successfully exchanged both in vivo and in vitro.[^15b^, ^24^]

Titration of MmpF with ^15^N MacpA showed distinct line broadening of peaks, indicative of an interaction between these proteins (Figure S7). An NMR response between TacpB and MmpF was also observed, although this was much less pronounced than with MacpA (Figure S7). On the other hand, ACP5, which was chosen as a representative ACP from the tridomain of MmpB, showed no discernible interaction with MmpF (Figure S7).

**Scheme 1:**
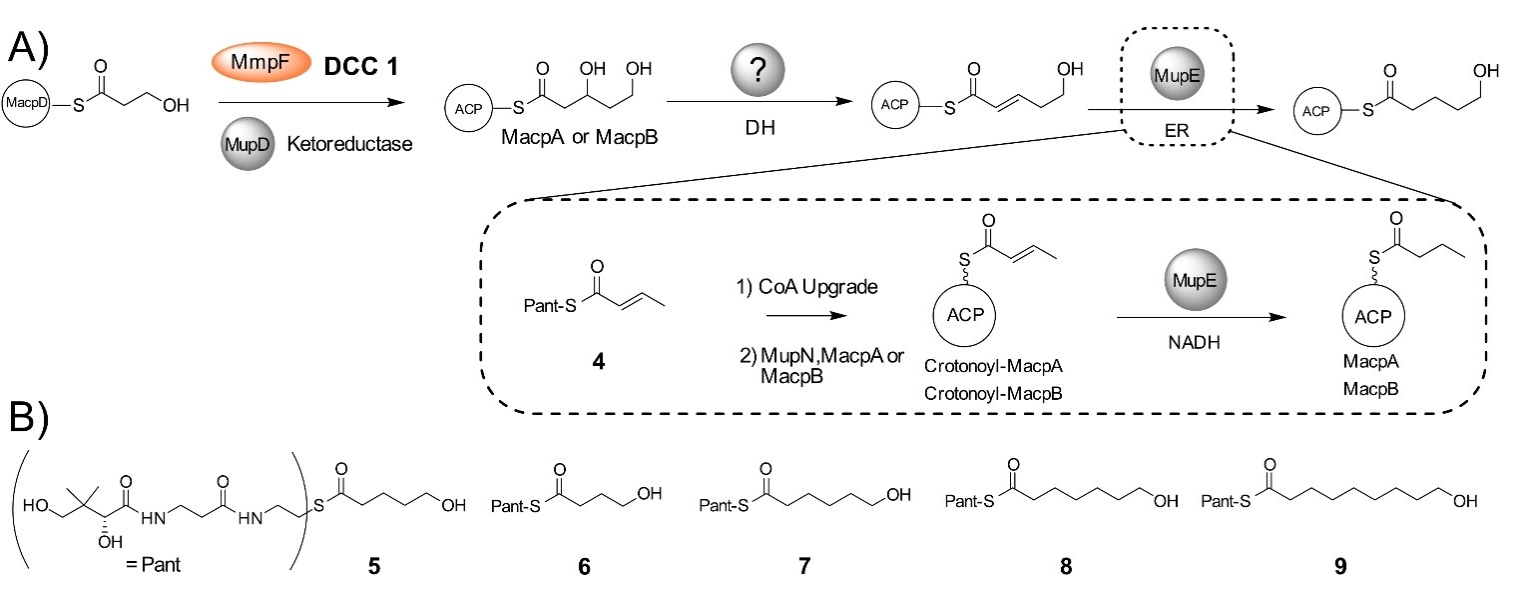
A) Conversion of 3‐hydroxyproprionate attached to MacpD to 5‐hydroxypentanoyl‐ACP. Investigations into MupE utilised crotonyl pantetheine. B) Synthetic acyl pantetheines.

**Figure 3:**
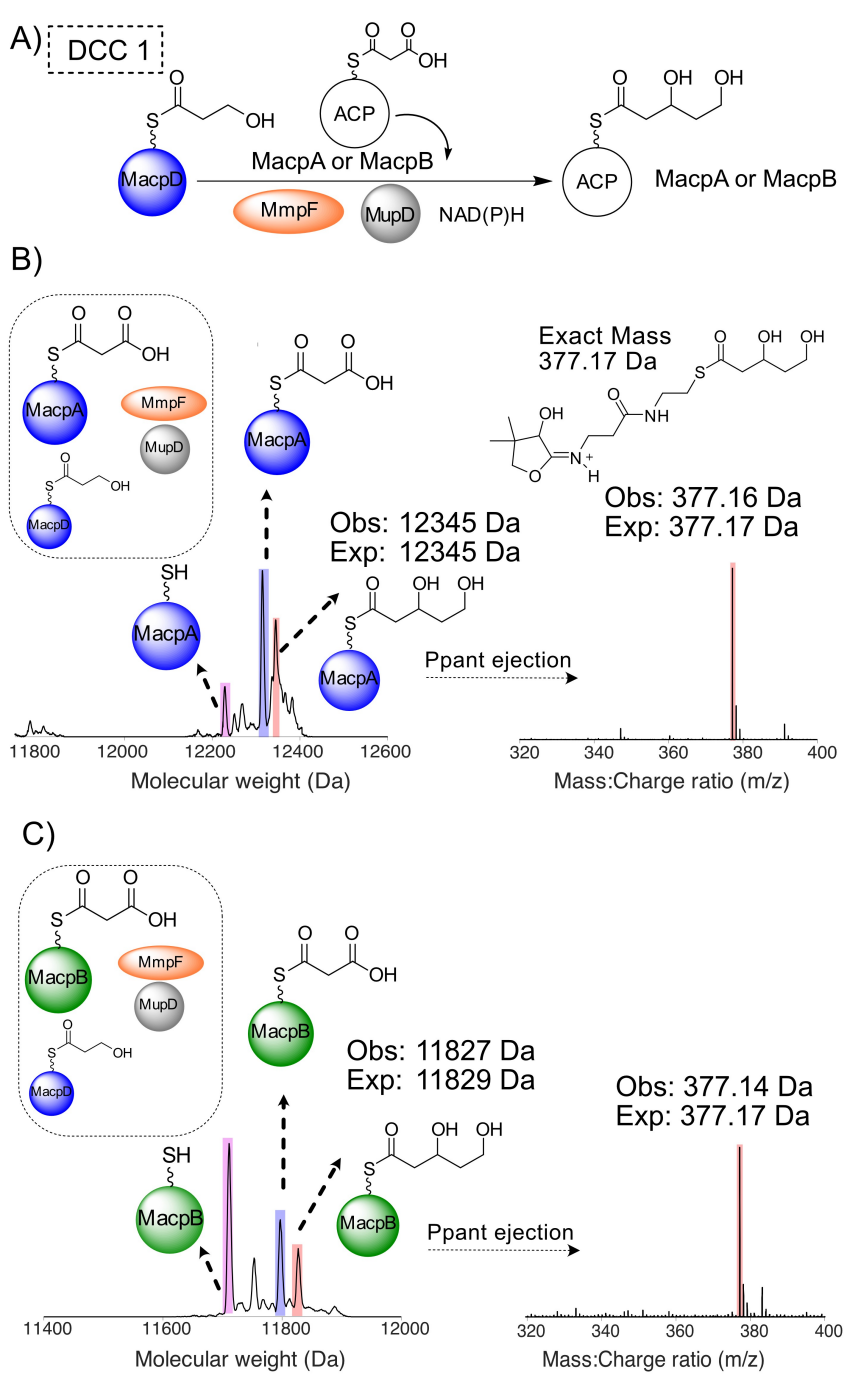
DCC 1—Generation of 3,5‐dihydroxypentanoyl‐ACP by MmpF and MupD. A) Decarboxylative Claisen condensation (DCC) of 3HP‐MacpD by MmpF and malonyl‐MacpA/B. B) Deconvoluted spectra and corresponding Ppant ejection for malonyl‐MacpA in the assay. C) Deconvoluted spectra and corresponding Ppant ejection for the malonyl‐MacpB assay.

Following evidence that MmpF was able to interact with MacpD, MacpA and (likely) MacpB, the ability of MmpF to catalyse DCC 1 (Scheme 1) was tested and monitored by ESI‐MS phosphopantetheine (Ppant) ejection assays. This MS‐MS technique fragments the derivatised phosphopanetheine arm from the ACP, thereby allowing accurate mass determination of low molecular weight species borne by the carrier protein.^[25]^ To assay DCC 1, samples of 3HP‐MacpD, malonyl‐MacpA and malonyl‐MacpB were prepared (Figure S9).^[14]^ It has been previously noted that in studies of fatty acid biosynthesis in *E. coli*, the presence of downstream β‐processing enzymes (eg the KR, DH, and ER) was critical in controlling the flux of condensation products. Bioinformatic analysis suggested MupD to be a putative *trans*‐acting KR that might fulfil this role by providing the first reductive step following chain extension. Hence the assay was also supplemented with purified MupD and NAD(P)H (Figure S10, S11; Figure 3A).

Incubation of malonyl‐MacpA with MmpF/MupD and 3HP‐MacpD yielded a new MacpA bound species (obs: 12 345 Da; exp: 12 345 Da) and Ppant ejection yielded an ion fragment consistent with a 3,5‐dihydroxypentanoyl‐MacpA parent (obs: 377.16 Da; exp: 377.17 Da) (Figure 3B). An analogous assay with malonyl‐MacpB also led to the formation of a C_5_ species (obs: 11 827 Da, exp: 11 829 Da). Ppant ejection again revealed a fragment at 377.14 Da consistent with generation of 3,5‐dihydroxypentanoyl‐MacpB (Figure 3C). As expected, in the absence of MupD, conversion to 5‐hydroxy‐3‐oxopentanoyl‐ACP was poor (Figure S12). Control experiments utilising a mutant of MmpF lacking the active site cysteine (C183A) failed to generate product (Figure S13). In summary, both malonylated ACPs were able to undergo the initial chain elongation and subsequent ketoreduction to the 5‐carbon fatty acid intermediate.

Following ketoreduction, the ACP‐bound acyl chain will be further processed by enzyme catalysed dehydration and enoyl reduction (Scheme 1A). However, dehydratase (DH) functionality mediated by a *trans*‐acting DH domain appears to be absent within this biosynthetic gene cluster. The ACP bound β‐hydroxythiolester was stable in our DCC 1 assays suggesting that the dehydration is unlikely to occur spontaneously. This raised the possibility that this activity might be supplied in a nonlinear fashion by one of the remaining modular DH domains in the mupirocin gene cluster with unassigned or proven function.^[26]^ In this category two modular DH domains were identified. The first was the dehydratase of MmpD (MmpD_DH1) which is skipped in polyketide assembly^[8]^ and the second, the DH domain of MmpB. Either may act through a nonlinear “long‐range” interaction on the nascent fatty acid chain. We therefore overexpressed and purified both excised domains (Figure S11). Inclusion of either of these proteins in an extended Claisen condensation assay (with 3HP‐MacpD, malonyl‐MacpB, MmpF and MupD) failed, however, to catalyse dehydration (Figure S14). Further bioinformatic analysis revealed neither of these modular DH domains (or indeed any of them) contained a 23 amino acid extension that has been associated with an additional “long‐range” dehydratase function (Figure S15).^[27]^ Therefore, we assume that FabA or FabZ‐like enzymes within *P. fluorescens* fatty acid biosynthesis may substitute for the missing DH activity.

Following dehydration, the final step of the cycle involves reduction of the enoyl moiety to yield the fully saturated fatty acid chain. Sequence analysis of MupE showed homology to the medium‐chain dehydrogenase/reductase (MDR) superfamily (Figure S16) and MupE has previously been annotated as an enoyl reductase.^[8]^ Interestingly a Δ*mupE* knockout strain produced mupirocin E that contained a single 6,7‐*E*‐alkene in the fatty acid chain, consistent with its role in this phase of the pathway.^[2]^ MupE was therefore expressed and purified (Figure S11) but attempts to produce (*E*)‐5‐hydroxypent‐2‐enoyl‐ACP (Scheme 1A) synthetically failed due to spontaneous cyclisation of the substrate. Hence a crotonyl mimic of the natural substrate as the crotonyl pantetheine thiol ester (**4**) was synthesised. Thiol ester **4** was chemoenzymatically upgraded onto both MacpA and MacpB and Ppant ejection yielded the expected ions (obs: 329.10 Da and 329.23 Da for MacpA/B respectively; exp: 329.15 Da). Each acyl‐ACP was then incubated with MupE and NADH (Figure S17) and Ppant ejection revealed formation of a new species with an increased mass of 331.23 Da (exp: 331.17 Da), consistent with the addition of two Da. This confirmed successful reduction of crotonoyl‐MacpA/B to the corresponding butanoyl species (Scheme 1A).

### MmpF Catalyses a Second Round of Fatty Acid Chain Extension

**Scheme 2:**
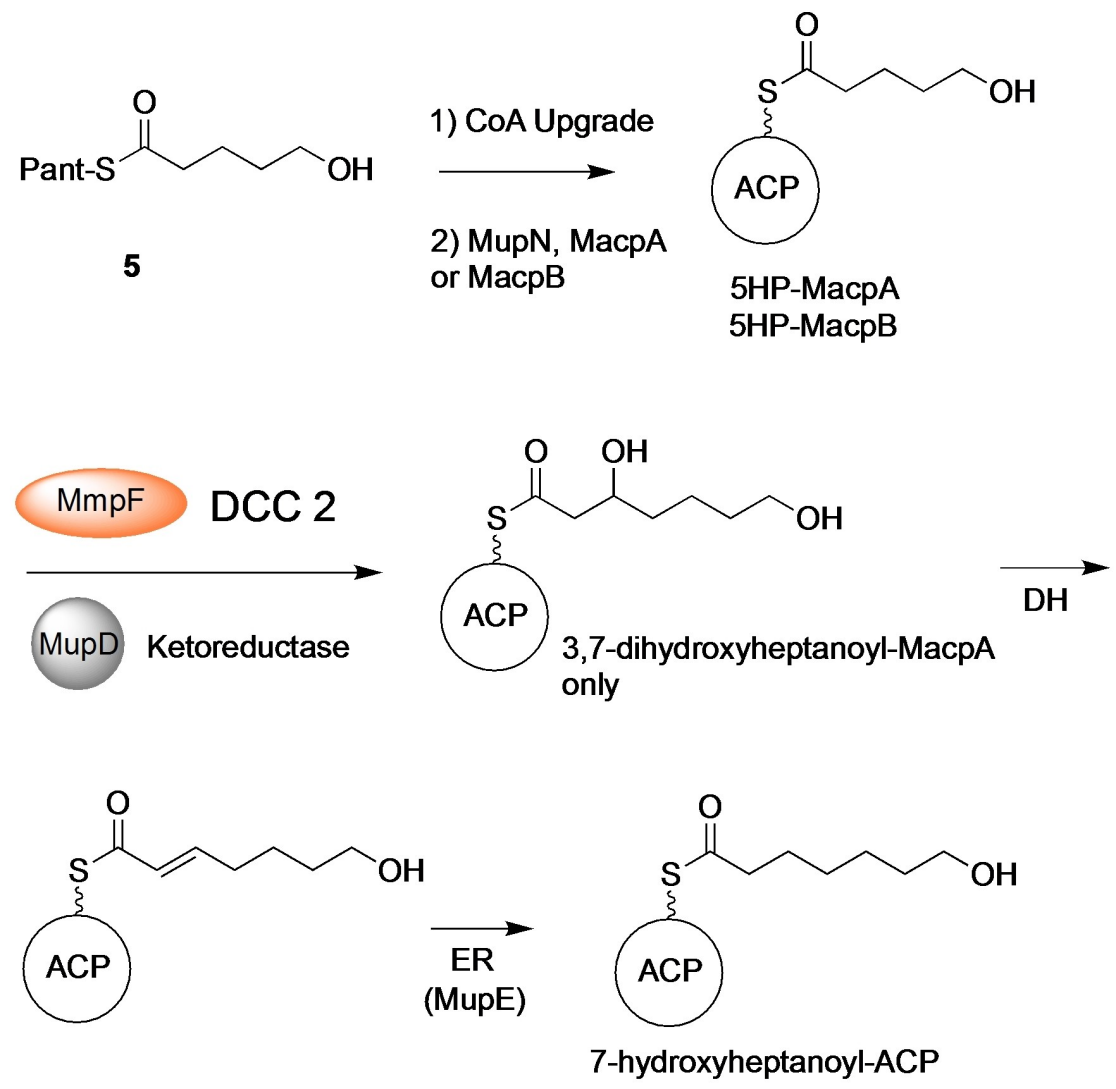
MmpF catalyses DCC 2 and alongside tailoring enzymes (KR, DH, ER) 5‐hydroxypentanoyl‐MacpA is converted to 7‐hydroxyheptanoyl‐ACP.

A further assay was designed to probe if MmpF acted in an iterative manner by successively catalysing DCC 1 and then DCC 2 to produce a 7‐hydroxy‐3‐oxoheptanoyl intermediate (Scheme 2). Initially 5‐hydroxypentanoyl (5HP) pantetheine thiol ester **5** was synthesised for direct use in the assay without an ACP. MmpF was incubated with an excess of **5** in the presence of MupD, NAD(P)H and either malonyl‐MacpA or ‐MacpB (as the acceptor) and the reaction monitored by ESI‐MS (Figure S18). For MacpA, a new species was formed (obs: 12 373 Da, exp: 12 374 Da) with a Ppant ejection fragment of mass 405.23 Da (exp: 405.21 Da), consistent with formation of 3,7‐dihydroxyheptanoyl‐MacpA. This species was not formed in a control reaction with the C183A MmpF mutant (Figure S18). Interestingly, assays utilising malonyl‐MacpB failed to yield 3,7‐dihydroxyheptanoyl‐MacpB suggesting that MmpF was selective for MacpA for DCC 2.

**Figure 4:**
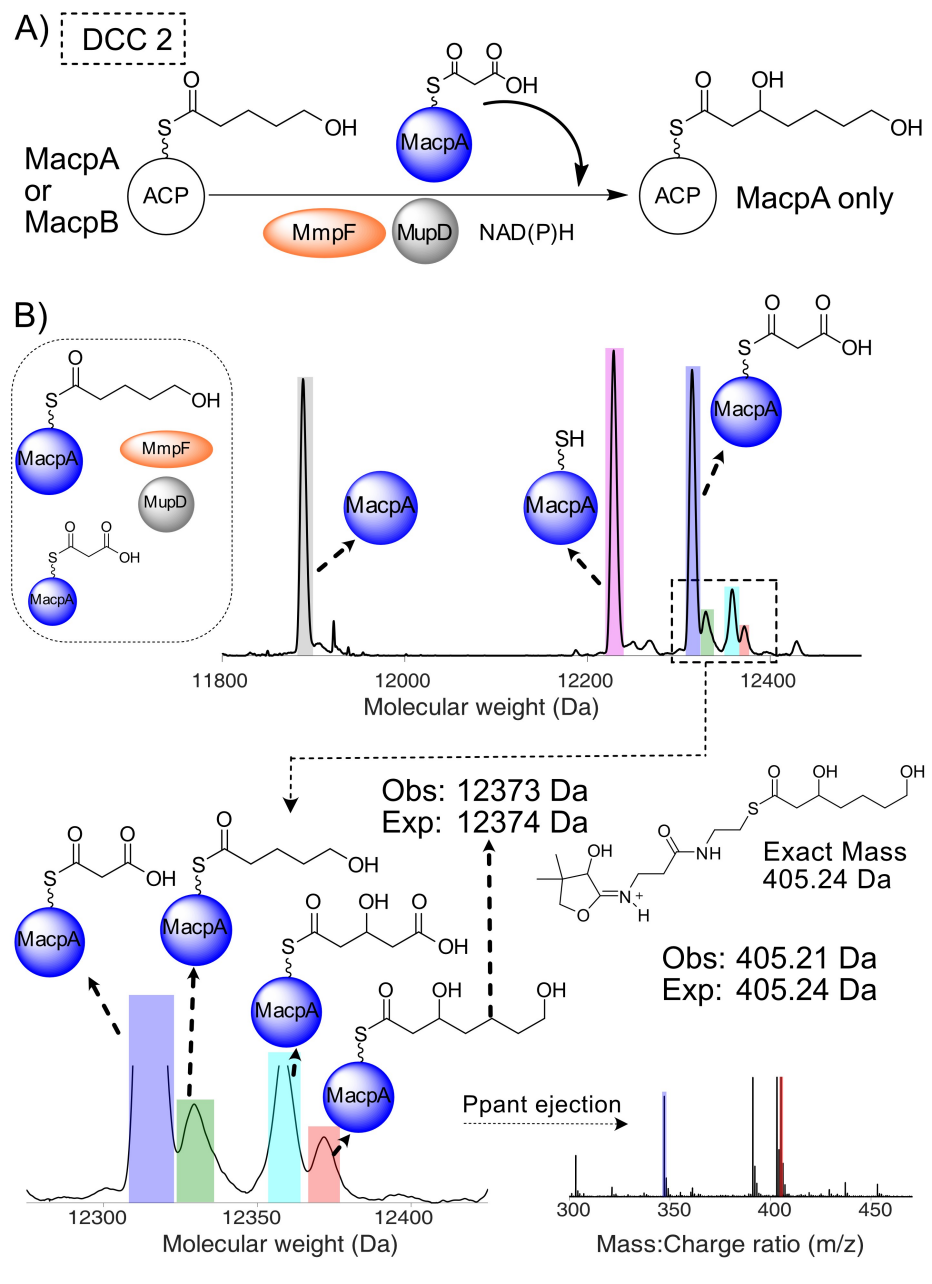
DDC 2—MacpA and MmpF function iteratively to produce 3,7‐dihydroxyheptanoyl‐MacpA. A) Proposed reaction scheme. B) Deconvoluted spectra and corresponding Ppant ejection of 5HP‐MacpA/malonyl‐MacpA, MmpF and MupD assay. A side reaction arising presumably from transfer of malonate to MmpF and DCC with malonyl‐MacpA followed by ketoreduction led to the generation of a significant proportion of MacpA bound 3‐hydroxypentan‐1,5‐dioic acid (cyan) and was also observed in reactions using preloaded MmpF (Figure S18).

Next, MacpA/B was tested for donor ACP activity and priming MmpF with 5HP. MacpA and B were upgraded with 5HP (Figure S19) and incubated with MmpF, malonyl‐MacpA, MupD and NAD(P)H (Figure 4). When 5HP‐MacpA was present, 3,7‐dihydroxyheptanoyl‐MacpA was again successfully generated (obs: 12 373 Da, exp 12 374 Da; Ppant ejection (405.24 Da, exp 405.21 Da)) (Figure 4B). Substitution with 5HP‐MacpB as the donor ACP failed to generate product. Instead 5HP‐MacpB was hydrolysed to *holo‐*MacpB (Figure S19) whilst malonyl‐MacpA levels remained constant.

### MmpB_KS is Responsible for the Final Extension to a 9‐Carbon Acyl Chain

Saturated hydroxylated fatty acids of varying chain lengths: 4‐hydroxybutanoate (4HB) (**6**), 6‐hydroxyhexanoate (6HH) (**7**), 7‐hydroxyheptanoate (7HH) (**8**) and 9‐hydroxynonanoate (9HN) (**9**) were synthesised as pantetheine analogues (Scheme 1) to probe the selectivity of MmpB_KS and the final DCC3 to yield 9‐hydroxynonanoic acid. Initially, self‐loading in vitro assays of MmpB_KS in the presence of an excess of pantetheine substrates containing an odd number of carbons revealed that MmpB_KS could be primed with 7HH but selected against 5HP and 9HN pantetheine analogues (Figure S20). Selectivity for 7HH was consistent with a model in which MmpB only performs the last round of fatty acid extension. Interestingly there was tolerance for the shortened, non‐natural, 6HH analogue (**7**) (obs+114 Da, exp+115 Da) although a comparatively significant reduction in self‐loading was observed. MmpB_KS was unable to self‐load with 4HB.

To determine if MmpB_KS was also able to catalyse the final fatty acid extension step (DCC 3), the individual MmpB ACP domains (ACP5, 6 and 7) were chemoenzymatically upgraded with malonyl CoA (Figure S21). Incubation of MmpB KS with an excess of 7HH (**8**) and malonyl‐ACP5 resulted in the formation of a new ACP5 bound species (obs: 12 884 Da) yielding a 431.23 Da Ppant ejection ion, consistent with the successful formation of a C_9_ 3‐ketothiol ester intermediate (exp: 431.22 Da and 12 884 Da) (Figure S22). Equivalent assays using either malonyl‐ACP6 or ‐ACP7 also formed the 9‐hydroxy‐3‐oxononanoyl ACP, confirming the three tandem ACPs were, at least in this context, functionally equivalent.^[28]^ Denaturing MmpB_KS completely abrogated this activity and both malonyl‐MacpA and B were unable to substitute for these ACPs (Figure S23A–C). MmpB_KS was unable to turnover using excess 5HP‐pantetheine (**5**) or 6HH‐pantetheine (**7**) despite the latter partially self‐loading (Figure S23). MmpB_KS is therefore only catalytically active with a C_7_ substrate.

**Figure 5:**
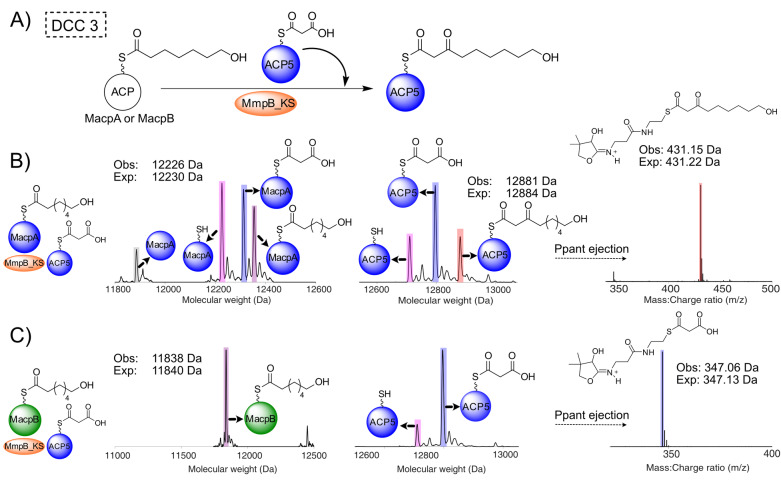
DCC 3—MacpA delivers a C7 chain to MmpB_KS which generates 9‐hydroxy‐3‐oxononanoyl‐ACP5. A) Proposed reaction scheme. B) Deconvoluted spectra of MacpA (left), ACP5 (centre) and the corresponding Ppant ejection (right) when 7HH‐MacpA is incubated with MmpB_KS and malonyl‐ACP5 to produce 9‐hydroxy‐3‐oxononanoyl‐ACP5. C) Deconvoluted spectra of MacpB (left), ACP5 (centre) and the corresponding Ppant ejection (right) when 7HH‐MacpB is incubated with MmpB_KS and malonyl‐ACP5. Key: apo ACP (grey), holo ACP (pink), malonyl‐ACP (blue), 7HH‐ACP (purple) and 9‐hydroxy‐3‐oxononanoyl‐ACP5 (red).

Finally, to demonstrate an authentic catalytic cycle, both MacpA and MacpB were upgraded with 7HH‐pantetheine (**8)** (Figure S24) and incubated with MmpB_KS and malonyl‐ACP5 (Figure 5). For 7HH‐MacpA this resulted in the formation of a new ACP5 bound species (obs: 12 881 Da, exp: 12 884 Da) (Figure 5B) and an ejection species at 431.15 Da (exp: 431.22 Da) derived from the desired parent 9‐hydroxy‐3‐oxononanoyl‐ACP. When 7HH‐MacpB was substituted as the donor ACP, no turnover was observed (Figure 5C). Analysis of the ESI‐MS spectra of 7HH‐MacpB in the presence of MmpB_KS showed it to be stable but unable to transfer 7HH to MmpB_KS. Lastly when a mixture of 7HH‐ACP5 and malonyl‐ACP5 was supplied, there was no turnover or transfer of 7HH from 7HH‐ACP5 to MmpB_KS. This confirmed supply of the 7HH intermediate is solely from MacpA and not an MmpB ACP, further corroborating that MmpB_KS only catalyses the final extension step (7HH‐ACP5 might arise if MmpB_KS could also catalyse conversion of 5HP to 7HH). Therefore, once MmpB_KS is primed with the 7HP, the tridomain ACPs are all able to act as malonyl donors for DCC 3 to yield 9‐hydroxy‐3‐oxononanoyl‐ACP. β‐processing of this fatty acid intermediate is then presumed to be completed by the MmpB KR and DH with the ER activity being supplied by the MmpC ER or possibly even MupE.

The role of MacpA in all three DCC steps firmly establishes the function of this ACP. Although MacpB appears to partially work in parallel to generate a 5HP intermediate, it is unable to affect a back‐transfer of 5HP to MmpF in preparation of DCC 2 nor catalyse later mechanistic steps which disfavours this model. Reinforcing this, our in vivo studies using single knock‐outs of *macpA or macpB* did not yield PA‐A, which might be expected if these ACPs acted in parallel and were partially redundant. Instead, Δ*macpA* yields mupirocin H and mupiric acid, consistent with an exclusive late‐stage role in mupirocin assembly whereas Δ*macpB* gave significantly reduced titres of all metabolites. This could therefore point to a role for MacpB in an early phase of the biosynthetic pathway, for example in priming the first KS of MmpD which lacks a GCN5‐related *N*‐acetyl transferase. It is also possible that MacpB is not malonylated in vivo and cannot take part in fatty acid biosynthesis. An alternate mechanism where instead MacpB is converted to acetyl‐MacpB would be consistent with a role in pathway priming.

The co‐linearity model that predicts each module of a Type I biosynthetic pathway performs one elongation and one set of tailoring steps does not apply in cases where there is a programmed iteration of domains, modules, or the entire PKS.^[29]^ In bacterial type I aromatic iterative PKSs (iPKS), multiple iterations involve the entire complex, yielding mono‐ or polycyclic aromatic polyketides. Conversely, partially iterative PKSs can invoke a programmed iteration or “stuttering” of a single module, where the downstream ACP in the PKS passes the polyketide intermediate back to the upstream KS for an additional round (or rounds) of elongation and β‐keto processing. This has been reported for several bacterial type I PKSs including aureothin,^[30]^ stigmatellin,^[31]^ and borellidin.^[32]^ To our knowledge there are only rare examples of iterative modules in *trans*‐AT PKSs and to‐date these involve the assembly of conjugated alkene portions of the polyketide.[^33^, ^34^]

**Figure 6:**
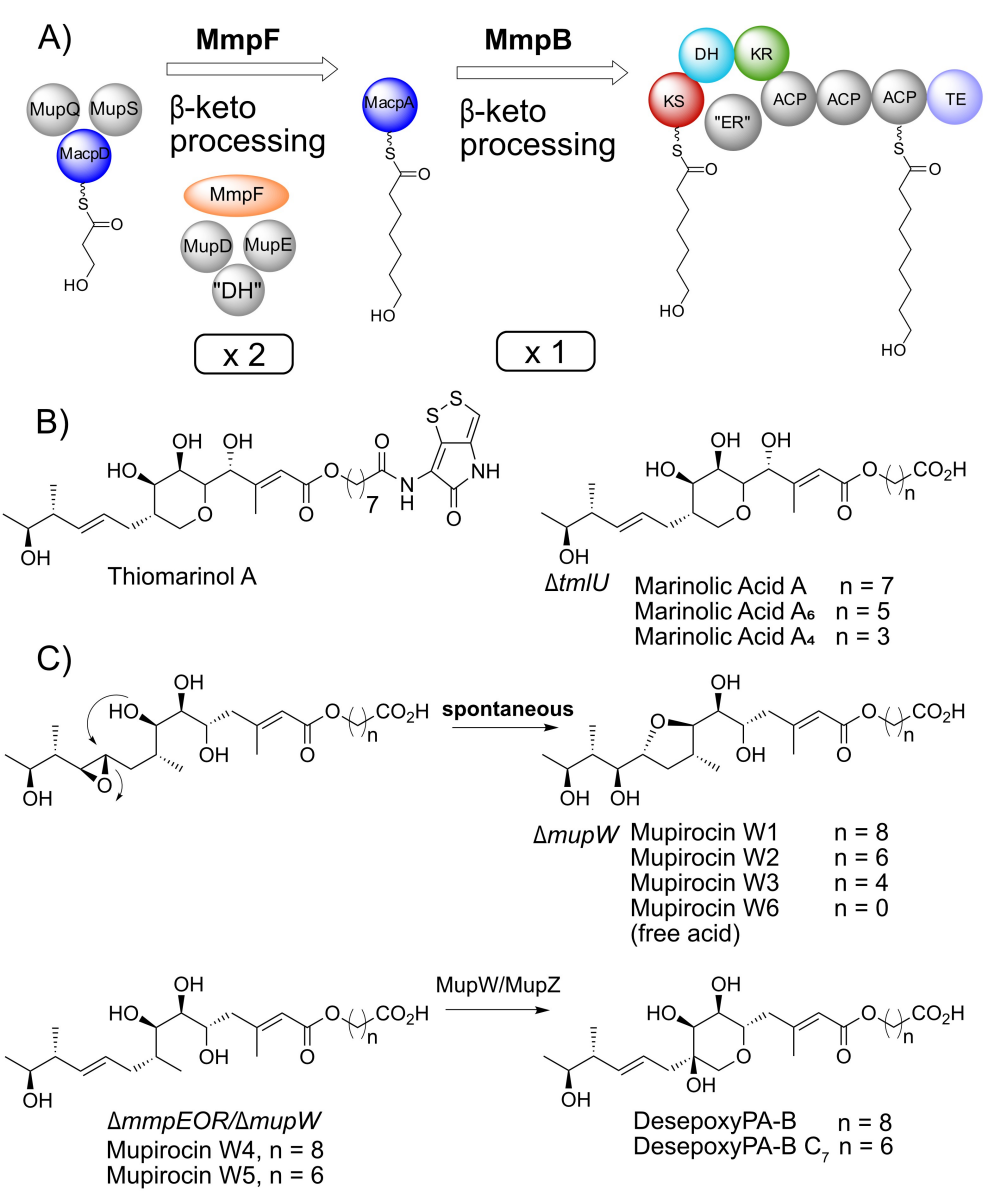
MmpF is a fully reducing stuttering module that controls FAS within mupirocin biosynthesis. A) MmpF catalyses two extensions of the 3HP‐MacpD starter unit, alongside *trans*‐acting tailoring enzymes, before MmpB exclusively mediates the extension of a C_7_ acyl chain to the 9‐hydroxynonanoic fatty acid. B) Thiomarinol A and metabolites produced by a Δ*tmlU* (CoA ligase) mutant. C) Metabolites from Δ*mupW* and Δ*mmpEOR*/Δ*mupW* mutants (MmpEOR is the oxidoreductase domain in MmpE responsible for epoxide formation).

MmpF, derived from a degraded *cis*‐AT KS‐AT didomain represents a new example of a fully reducing stuttering module that has been acquired by a *trans*‐AT system (Figure 6A). Uniquely this is not embedded in a larger multi‐functional module but as a standalone unit that has certain interesting implications. For the *cis*‐AT PKSs, programmed iteration requires the iterating KS to remain free and not to accept a new starter unit before the polyketide is returned. This is presumably under kinetic and steric control^[35]^. The action of MmpF essentially resembles bacterial Type II fatty acid biosynthesis where a full reductive cycle of β‐keto processing occurs before the fatty acid is returned to a KS for the next elongation step (Figure 6A). The number of iterations performed may be guided by a combination of the elongation kinetics and noncovalent protein–protein interactions alongside a chain‐length gatekeeping role of the downstream KS.^[33]^ Elegant experiments involving the expression of *aurA*, the gene encoding the iterating module of the aureothin PKS from *Streptomyces thioluteus*, in a heterologous host led to the observation of additional iterations (up to four) in the absence of gatekeeper control (the downstream KS in AurB).^[36]^ For MmpF and MacpA, the MmpB_KS functions as a strict gatekeeper, recognising and transferring only the 7HH‐MacpA intermediate.

TmpF, a homolog of MmpF, is present in the biosynthetic pathway of the structurally related thiomarinols (the major metabolite thiomarinol A is shown in Figure 6B). These metabolites contain a similar polyketide scaffold esterified to a 8‐hydroxyoctanoyl chain (8HO) that is *N*‐linked to a pyrrothine moiety from the holomycin class of antibiotics.^[15]^ The fatty acid side chain is primed from a 4‐hydroxybutanoyl (4HB) starter unit as opposed to 3HP in mupirocin and therefore requires two rounds of fatty acid extension. Presumably therefore, TmpF may act only once to produce 6‐hydroxyhexanoyl‐TacpA which subsequently undergoes a final round of elongation on TmpB, the functional equivalent of MmpB. In this instance TmpF may be unable to perform a second round of fatty acid elongation or the system may be under strict gatekeeper control by TmpB that ensures rapid substrate transfer from 6‐hydroxyhexanoyl‐TacpA. Interestingly, although the thiomarinol biosynthetic pathway contains functional homologs of the mupirocin fatty acid generating enzymes, it is missing equivalents of MupD (KR) and MupE (ER) as well as a discrete DH meaning it lacks all of the initial β‐ketoprocessing enzymes. Again, these are presumably supplied by enzymes utilised in fatty acid biosynthesis in *Pseudoalteromonas* SANK73390.

**Figure 7:**
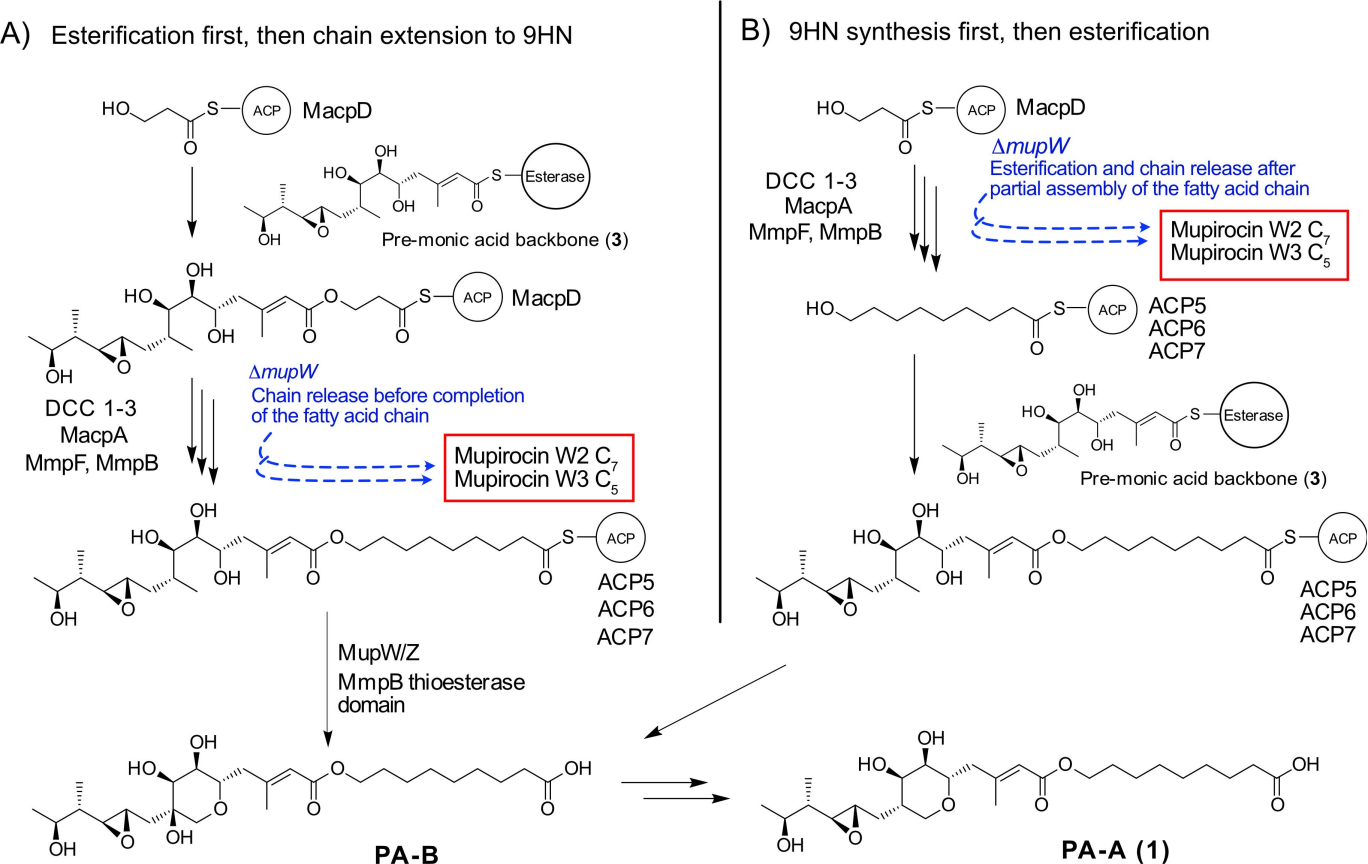
Different pathways towards the coupling of fatty acid synthesis and esterification. Pathway A) 3HP‐MacpD is presumed to be esterified to a pre‐monic acid moiety before fatty acid synthesis. B) Alternative pathway proposed within this study.

A key question is the timing of fatty acid chain extension versus esterification and THP ring closure. Previous work using a knock‐out of the Rieske non‐haem oxygenase MupW and/or combinations of other late stage acting enzymes led to the isolation of low titres of metabolites lacking a THP ring (eg mupirocin W1).[^10b^, ^12^] A number of these ring open metabolites also possessed shortened fatty acid chains, eg mupirocin W3 and W2 with C_5_ and C_7_ chains respectively (Figure 6C, note all of the isolated metabolites contain an aberrant tetrahydrofuran ring generated by attack of the 7‐OH on the 10,11‐epoxide). In addition, all metabolites isolated to date from cultures of *P. fluorescens* which incorporate the THP ring also have the full 9‐hydroxynonanoic acid side chain. These initial observations suggested the 3HP fatty acid starter unit is first esterified, then sequentially elongated to a nine carbon chain and the full fatty acid side chain is essential for THP formation (Figure 7A). More recent studies feeding acyclic substrates with C_7_ and C_9_ fatty acid side chains to *E. coli* strains expressing MupW and MupZ (Figure 6C, the epoxide hydrolase MupZ controls 6‐ versus 5‐ membered ring formation) showed these compounds were converted to their THP ring counterparts. These results supported this sequence but also indicated tolerance for a shorter C_7_ chain.^[10a]^ Analogous THP containing marinolic acid structures have been isolated with different length fatty acids from *Pseudoalteromonas* SANK73390 suggesting TmlW/TmlZ are either more promiscuous or ring closure precedes fatty acid extension.^[13]^

The in vitro results in this study support a different mechanism in which the fatty acid extension phase occurs first, followed by esterification and then ring closure to yield PA‐B (Figure 7B). The existence of low titre metabolites, such as mupirocin W2 and W3, with curtailed fatty acid chains arise in gene knock‐out studies when the pathway is defective or stalled and esterification occurs prematurely. For example, MacpA, which carries both 5HP and 7HP intermediates, may have weak activity with the esterase yielding these minor products when the normal metabolite flux has been significantly perturbed. The lack of any intermediates with a C_3_ fatty acid side chain might suggest that esterification reactions involving 3HP‐MacpD may be disfavoured, although it cannot be ruled out that the product is unstable and readily undergoes hydrolysis. Finally, it could also be argued that MmpF or MmpB might elongate an esterified substrate, starting from the 3HP ester. Although plausible, the strict chain length selectivity observed for MmpB would disfavour this hypothesis as it is unlikely to accept such a bulky substrate.

## Conclusion

The work presented here provides an extensive investigation into the stepwise assembly of the fatty acid (9HN) moiety of mupirocin. Following starter unit generation (3HP) on MacpD, MmpF catalyses two further two carbon extensions (DCC1 and DCC2) in conjunction with the necessary β‐processing of the fatty acid chain. Tailoring enzymes for KR and ER β‐processing steps have been identified as MupD and MupE respectively but a DH activity remains elusive. MacpA and MacpB are able to function in DCC1, but MacpA exclusively mediates DCC2. The MmpB_KS catalyses the final round of fatty acid extension, DCC3, to yield a C_9_ chain. In summary, these studies have revealed the function of several unannotated ACP and KS domains that form a sequential pathway for fatty acid synthesis. Several key questions surrounding the pathway remain, but this study marks a major step in closing in on a “blueprint” for the enigmatic biosynthetic sequence of mupirocin.

## Conflict of interest

The authors declare no conflict of interest.

1

## Supporting information

As a service to our authors and readers, this journal provides supporting information supplied by the authors. Such materials are peer reviewed and may be re‐organized for online delivery, but are not copy‐edited or typeset. Technical support issues arising from supporting information (other than missing files) should be addressed to the authors.

Supporting Information

## Data Availability

The data that support the findings of this study are available from the corresponding author upon reasonable request.
