# Supplementary material for: Programmed Iteration Controls the Assembly of the Nonanoic Acid Side Chain of the Antibiotic Mupirocin
Source: Angew Chem Int Ed Engl. 2022 Nov 10;61(50):e202212393. doi: 10.1002/anie.202212393 (PMC10098928; doi:10.1002/anie.202212393)
Supplement: Supplementary file 1 — Supporting Information [file ANIE-61-0-s001.pdf]

Angewandte Chemie
Eine Zeitschrift der Gesellschaft Deutscher Chemiker

Supporting Information

Programmed Iteration Controls the Assembly of the Nonanoic Acid Side Chain of the Antibiotic Mupirocin

A. J. Winter, M. T. Rowe, A. N. M. Weir, N. Akter, S. Z. Mbatha, P. D. Walker, C. Williams, Z. Song, P. R. Race, C. L. Willis, M. P. Crump**

1. Fermentation procedure for mutant strains of *Pseudomonas fluorescens* NCIMB 10586

The Δ*macpA*, Δ*macpB* and Δ*mmpF_KS10* mutant strains[1] of *P. fluorescens* NCIMB 10586 were incubated on LB agar plates (1% Bacto tryptone, 0.5% yeast extract, 0.5% sodium chloride, 2% agar) supplemented with tetracycline (30 μg/ml) and incubated at 30°C for 48 hrs. The seed medium was prepared in 100 ml of LB media (1% Bacto tryptone, 0.5% yeast extract, 0.5% sodium chloride, 1% glucose) in a 500 ml flask. The seed medium contained 30 μg/ml tetracycline and was inoculated with a single colony from a freshly incubated plate and incubated at 200 rpm, 25°C for 20 hrs.

Fermentation was inoculated with 1% seed culture in modified LB medium (1% Bacto tryptone, 0.5% yeast extract, 0.5% sodium chloride) supplemented with 4% w/v glucose and 0.5 μg/ml IPTG (3 x 100 ml) in 500 ml baffled flasks. The culture was incubated at 200 rpm at 22°C for 50 hrs then centrifuged at 8000 rpm for 15 min. The supernatant was extracted with EtOAc three times and the combined extracts were evaporated *in vacuo* to give a crude extract, which was resuspended in MeOH for LC-MS analysis.

2. General Experimental Procedures for chemical synthesis

All reagents and solvents were obtained from commercial sources and were used as purchased. Petroleum ether is of the 40 – 60 °C boiling point range. Where anhydrous conditions were necessary, reactions were carried out in flame-dried glassware under a positive pressure of nitrogen using standard Schlenk syringe-septa techniques. Anhydrous solvents were obtained from an Anhydrous Engineering Ltd. modified Grubbs system of double alumina and alumina copper catalysed drying columns. [2]

Routine monitoring of reactions was conducted by analytical TLC using aluminium sheets precoated with silica (MerckKieselgel 60 F_{254}) with a suitable solvent system and visualised using 254 nm UV light and/or developed with potassium permanganate and heat. Flash column chromatography was performed according to the procedure used by Still *et al.* [3] Infrared spectra were recorded on a Perkin Elmer Spectrum 100 FTIR spectrometer with an ATR diamond cell and frequencies are reported in wavenumbers (cm^{-1}). Only strong and selected absorbances are reported. Mass spectrometry was performed by the University of Bristol mass spectrometry service by electrospray ionisation (ESI) or atmospheric pressure chemical ionisation (APCI) using a Bruker MicrOTOF II or Thermo Scientific Orbitrap Elite spectrometer.

^{1}H and ^{13}C NMR spectra were recorded using JEOL ECS 400 (400 MHz), JEOL ECZ 400 (400 MHz), JEOL-VAR ECZ 400 (400 MHz), Bruker Advance III HD 500 Cryo (500 MHz) and Varian VNMR S500 spectrometers. NMR samples were analysed as solutions with the solvent specified at ambient temperature and referenced to residual solvent peaks. Chemical shifts (δ) are reported in parts per million (ppm) and

coupling constants (*J*) are reported to the nearest 0.5 Hz. Assignments were made with the aid of 2D NMR experiments. Unless stated, data for all known compounds agree with published data.

General Procedure 01 for monoprotection of diols (GP01)

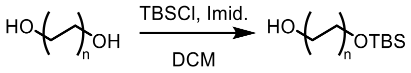

TBSCl (1 eq.) and imidazole (1 eq.) were added portion wise to a solution of diol (1 eq.) under an inert atmosphere in anhydrous DCM (0.5 M) at 0 °C and the mixture was warmed to rt and stirred for 16 h. Et_2O (1 *v/v*) was added and the organic phase washed with water (3 *v/v*). The organic layer was dried ($MgSO_4$) and the solvent removed *in vacuo*. The crude material was purified by flash chromatography (0-10% EtOAc in petroleum ether) to give the mono-TBS-protected diols.

6-((tert-butyldimethylsilyl)oxy)hexan-1-ol (S2)

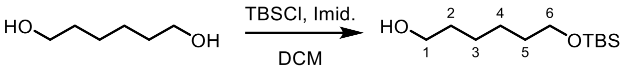

S1 **S2**

According to **GP01**, reaction of 1,6-hexanediol **(S1)** (1.00 g, 8.46 mmol), TBSCl (1.27 g, 8.46 mmol) and imidazole (575 mg, 8.46 mmol) in DCM gave the title compound **S2** (202 mg, 10.3 % yield) as a pale-yellow oil. **δ_H (400 MHz, $CDCl_3$)** 3.67 – 3.57 (4H, m, 1-H_2, 6-H_2), 1.61 – 1.49 (4H, m, 2-H_2, 5-H_2), 1.41 – 1.31 (5H, m, 3-H_2, 4-H_2, O*H*), 0.89 (9H, s, SiC(C*H*$_3$)$_3$), 0.04 (6H, s, Si(C*H*$_3$)$_2$). **δ_C (100 MHz, $CDCl_3$)** 63.3, 63.1 (C-1 & C-6), 33.0, 32.9 (C-2 & C-5), 26.1 (SiC(*C*H$_3$)$_3$), 25.8, 25.7 (C-3 & C-4), 18.5 (Si*C*(CH$_3$)$_3$), -5.1 (Si(*C*H$_3$)$_2$. **HRMS** (ESI+) calc. for $[C_{12}H_{28}NaO_2Si]^+$ 255.1756 found 255.1760.

All data are in accordance with the literature.[4]

7-((tert-butyldimethylsilyl)oxy)heptan-1-ol (S4)

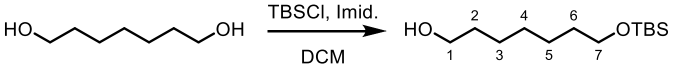

S3 **S4**

According to **GP01**, reaction of 1,7-heptanediol **S3** (1.00 g, 7.56 mmol), TBSCl (1.14 g, 7.56 mmol) and imidazole (514 mg, 7.56 mmol) in DCM gave the title compound **S4** (724 mg, 43.8 % yield) as a pale-yellow oil. **δ_H (400 MHz, $CDCl_3$)** 3.63 – 3.55 (4H, m, 1H_2, 7-H_2), 1.59 – 1.42 (4H, m, 2-H_2, 6-H_2), 1.36 – 1.27 (6H, m, 3-H_2, 4-H_2, 5-H_2), 0.87 (9H, s, SiC(CH$_3$)$_3$), 0.03 (6H, s, Si(CH$_3$)$_2$). **δ_C (100 MHz, $CDCl_3$)** 63.4, 63.0 (C-1 & C-7), 32.9, 32.8 (C-2 & C-6), 29.3 (CH_2), 26.1 (SiC(*C*H$_3$)$_3$, 25.9 (CH_2), 25.8 (CH_2), 18.5 (Si*C*(CH$_3$)$_3$), -5.2 (Si(*C*H$_3$)$_2$). **HRMS** (ESI+) calc. for $[C_{13}H_{31}O_2Si]^+$ 247.2093 found 247.2099.

All data are in accordance with the literature. [5]

9-((tert-butyldimethylsilyl)oxy)nonan-1-ol (S6)

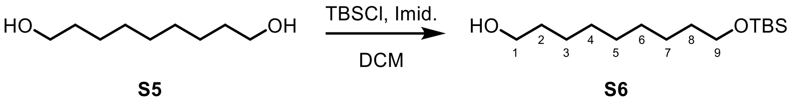

According to **GP01**, reaction of 1,9-nonanediol **S5** (2.16 g, 13.5 mmol), TBSCl (1.36 g, 9.0 mmol) and imidazole (610 mg, 9.0 mmol) in DCM gave the title compound **S6** (1.44 g, 54% yield) as a pale-yellow oil. **δ_H (400 MHz, $CDCl_3$)** 3.63 (2H, t, *J* 6.5, 1-H_2), 3.59 (2H, t, *J* 6.5, 9-H_2), 1.60 – 1.52 (2H, m, 2-H_2), 1.52 – 1.46 (2H, m, 8-H_2), 1.33 – 1.28 (10H, m, 5 x CH_2), 0.89 (9H, s, $SiC(CH_3)_3$), 0.04 (6H, s, $Si(CH_3)_2$). **δ_C (100 MHz, $CDCl_3$)** 63.5 (C-9), 63.2 (C-1), 33.0 (C-8), 32.9 (C-2), 29.7 (CH_2), 29.5 (CH_2), 29.5 (CH_2), 26.1 ($SiC(\textit{C}H_3)_3$), 25.9 (CH_2), 25.9 (CH_2), 18.5 ($Si\textit{C}(CH_3)_3$), -5.1($Si(\textit{C}H_3)_2$). **HRMS** (ESI+) calc. for $[C_{15}H_{34}NaO_2Si]^+$ 297.2226 found 297.2223.

All data are in accordance with the literature. [6]

General Procedure 02 for oxidation to carboxylic acids (GP02)

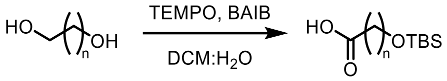

BAIB (2.86 eq.) and TEMPO (0.3 eq.) were added to a solution of mono-TBS-protected diol (1 eq.) in DCM (1 *v*) and water (1 *v*) and the reaction was stirred vigorously at ambient temperature for 16 h. The reaction was quenched (sat. aq. NaS_2O_3, 1 *v/v*), extracted with EtOAc (2 *v/v*) and the organic layer washed with water (3 x 2 *v/v*), brine (3 x 2 *v/v*), dried ($MgSO_4$) and the solvent removed *in vacuo*. The crude material was purified by flash chromatography (5-15% EtOAc in petroleum ether) to afford the desired acids.

5-((tert-Butyldimethylsilyl)oxy)pentanoic acid (S8)

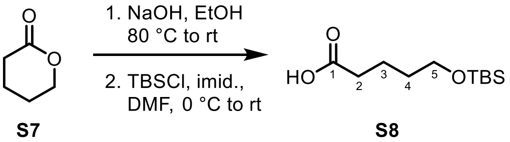

NaOH (2.20 g, 54.9 mmol) was dissolved in EtOH (100 mL) at 0 °C and valerolactone **S7** (4.63 mL, 49.9 mmol) was added dropwise. The mixture was heated at 80 °C for 0.5 h before cooling to rt and stirring for a further 2 h. The solvent was removed *in vacuo* to give the sodium salt (8.23g, quant).

The residue (8.23 g, 50.8 mmol) was suspended in DMF (100 mL) and cooled to 0 °C. TBSCl (15.3 g, 102 mmol) and imidazole (11.4 g, 168 mmol) were added sequentially, and the reaction was stirred overnight at rt. The reaction was quenched with H_2O (100 mL) and hexane (150 mL) was added. The layers were separated, and the aqueous layer was extracted with hexane (2 × 150 mL). The combined organics were dried over $MgSO_4$ and concentrated *in vacuo* to give diprotected product (10.19 g, 58%) as a colourless oil.

The crude deprotected product (10.19 g, 29.4 mmol) was dissolved in MeOH (30 mL) and THF (59 mL). K_2CO_3 (8.13 g, 58.8 mmol in 98 mL H_2O) was added and the reaction was stirred at rt overnight. The solution was cooled to 0 °C and acidified to pH 2 with 1 M HCl before extracting with Et_2O (3 × 200 mL). The combined organics were dried over $MgSO_4$ and concentrated *in vacuo* to give the title compound **S8** as a colourless oil (6.64 g, 97%). **δ_H (400 MHz, $CDCl_3$)** 3.63 (2H, t, *J* 6.0, 2-H_2), 2.39 (2H, t, *J* 7.5, 5-H_2), 1.76 – 1.65 (2H, m, 3-H_2), 1.62 – 1.52 (2H, m, 4-H_2), 0.89 (9H, s, $SiC(CH_3)_3$), 0.05 (6H, s, $Si(CH_3)_2$). **δ_C (100 MHz, $CDCl_3$)** 179.6 (C-1), 62.8 (C-5), 33.9 (C-2), 32.1 (C-4), 26.1 (Si(*C*H_3)$_2$), 21.4 (C-3), 18.5 (Si*C*(CH_3)$_3$, -5.2 (Si(*C*H_3)$_2$). **ν_{max}/cm^{-1} (film)** 3064 (br), 2954, 2930, 2858, 1710, 1254, 1105, 835, 775. **HRMS** (ESI+) calculated for [$C_{11}H_{24}O_3SiNa$] 255.1387 found 255.1399.

All data are in accordance with the literature. [7]

6-((tert-butyldimethylsilyl)oxy)hexanoic acid (S9)

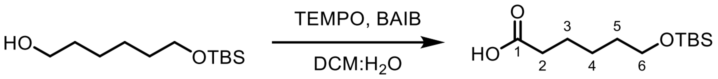

S2 **S9**

According to **GP02**, reaction of 6-((*tert*-butyldimethylsilyl)oxy)hexan-1-ol **S2** (150 mg, 0.645 mmol), TEMPO (30.3 mg, 0.194 mmol) and BAIB (623 mg, 1.94 mmol) in DCM and H_2O gave the title compound **S9** (81.0 mg, 51 % yield) as a colourless oil. **δ_H (400 MHz, $CDCl_3$)** 3.61 (2H, t, *J* 6.5, 6-H_2), 2.36 (2H, t, *J* 7.5, 2-H_2), 1.66 (2H, p, *J* 7.5, 3-H_2), 1.58 – 1.49 (2H, m, 5-H_2), 1.44 – 1.34 (2H, m, 4-H_2), 0.89 (s, 9H), 0.04 (s, 6H). **δ_C (100 MHz, $CDCl_3$)** 179.6 (C-1), 63.1 (C-6), 34.1 (C-2), 32.6 (C-5), 26.1 (Si(*C*H_3)$_3$), 25.5 (C-4), 24.7 (C-3), 18.5 (Si*C*(CH_3)$_3$, -5.1 (Si(*C*H_3)$_2$). **HRMS** (ESI+) calc. for [$C_{12}H_{26}NaO_3Si$]$^+$ 269.1549 found 269.1550.

All data are in accordance with the literature. [8]

7-((tert-butyldimethylsilyl)oxy)heptanoic acid (S10)

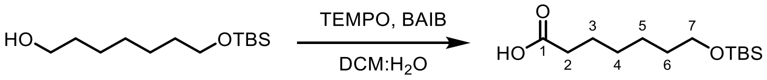

S4 **S10**

According to **GP02**, reaction of 7-((*tert*-butyldimethylsilyl)oxy)heptan-1-ol **S4** (500 mg, 2.29 mmol), TEMPO (107 mg, 0.3 mmol) and BAIB (2.21 g, 6.87 mmol) in DCM and H_2O gave the title compound **S10** (270 mg, 45.2 % yield) as a colourless oil. **δ_H (400 MHz, $CDCl_3$)** 3.60 (2H, t, *J* 6.5, 7-H_2), 2.35 (2H, t, *J* 7.5, 2-H_2), 1.70 – 1.59 (2H, m, 3-H_2), 1.56 – 1.46 (2H, m, 6-H_2), 1.41 – 1.28 (4H, m, 4-H_2, 5-H_2), 0.89 (9H, s, $SiC(CH_3)_3$), 0.04 (6H, s, $Si(CH_3)_2$). **δ_C (100 MHz, $CDCl_3$)** 179.8 (C-1), 63.2 (C-7), 34.1 (C-2), 32.7 (C-6), 29.0 (C-4), 26.1 (SiC(*C*H_3)$_3$), 25.6 (C-5), 24.8 (C-3), 18.5 (Si*C*(CH_3)$_3$), -5.2 (Si(*C*H_3)$_2$). **HRMS** (ESI+) calc. for [$C_{13}H_{29}O_3Si$]$^+$ 261.1886 found 261.1872.

All data are in accordance with the literature. [9]

9-((tert-butyldimethylsilyl)oxy)nonanoic acid (S11)

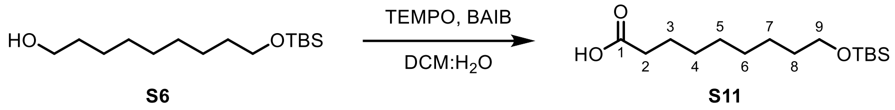

According to **GP02**, reaction of 9-((*tert*-butyldimethylsilyl)oxy)nonan-1-ol **S6** (1.00 g, 3.64 mmol), TEMPO (0.16 g, 1.04 mmol) and BAIB (3.35 g, 10.4 mmol) in DCM and H_2O gave the title compound **S11** (0.51 g, 48 % yield) as a colourless oil. **δ_H (500 MHz, $CDCl_3$)** 3.59 (2H, td, *J* = 6.5, 1.0, 9-H_2), 2.34 (2H, t, *J* = 7.5, 2-H_2), 1.62 (2H, p, *J* = 7.5, 8-H_2), 1.50 (2H, p, *J* = 6.5 Hz, 3-H_2), 1.39 – 1.27 (8H, m, 4-H_2, 5-H_2, 6-H_2, 7-H_2), 0.89 (9H, s, $SiC(CH_3)_3$), 0.04 (6H, s, $Si(CH_3)_2$). **δ_C (125 MHz, $CDCl_3$)** 180.3 (C-1), 63.4 (C-9), 34.2 (C-2), 32.9 (C-3), 29.4 (C-5, C-6), 29.1 (C-4), 26.1 ($SiC(\mathit{C}H_3)_3$), 25.9 (C-7), 24.8 (C-8), 18.5 ($Si\mathit{C}(CH_3)_3$), -5.1 ($Si(\mathit{C}H_3)_2$). **HRMS** (ESI+) calc. for $[C_{15}H_{32}NaO_3Si]^+$ 311.2018 found 311.2017.

All data are in accordance with the literature. [6]

General Procedure 03 for Thioester Coupling (GP03)

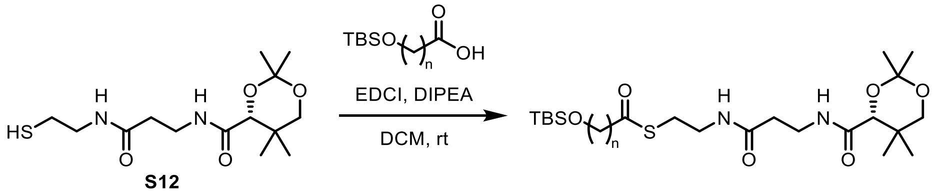

Carboxylic acid (1.2 equiv.) and EDCI (1.5 equiv.) were added to a solution of thiol **S12** (1.0 equiv.) – prepared as previously described[10] in anhydrous DCM under an inert atmosphere. DIPEA (2.0 equiv.) was added dropwise, and the reaction mixture was stirred for 16 h at rt. The solvent was removed *in vacuo* and the crude material was purified by flash chromatography (80-90% EtOAc in petrol or 0-10% MeOH in DCM) to give the protected coupled protected pantetheines.

Acetonide of 4-Hydroxybutanoic pantetheine (S14)

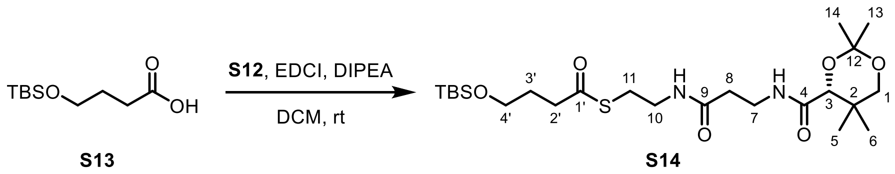

According to **GP03**, reaction of acid **S13** (83.0 mg, 0.38 mmol), EDCI (74.1 mg, 0.48 mmol), thiol (100 mg, 0.32 mnol) and DIPEA (110 µL, 0.64 mmol) gave the title compound **S14** (52 mg, 31.5%) as a viscous colourless oil. **δ_H (400 MHz, $CDCl_3$)** 7.01 (1H, d, *J* 7.5, NH), 6.15 (1H, s, NH), 4.07 (1H, s, 3-H), 3.67 (1H, d, *J* 11.5, 1-*H*H), 3.62 (2H, t, *J* 6.0, 4'-H_2), 3.62 – 3.45 (2H, m, 7-H_2), 3.48 – 3.36 (2H, m, 10-H_2), 3.27 (1H, d, *J* 11.5, 1-H*H*), 3.00 (2H, t, *J* 6.5, 11-H_2), 2.66 (2H, t, *J* 7.5, 2'-H_2), 2.42 (2H, t, *J* 6.0, 8-H_2), 1.85 (2H, m, 3'-H_2), 1.48 – 1.43 (3H, m, 13-H_3), 1.41 (3H, s, 14-H_3), 1.03 (3H, s, 5-H_3), 0.96 (3H, s, 6-H_3), 0.88 (9H, s, $SiC(C\mathit{H}_3)_3$), 0.04 (6H, s, $Si(C\mathit{H}_3)_2$). **δ_C (100 MHz, $CDCl_3$)** 199.8 (C-1'), 171.3 (C-4), 170.3 (C-9), 99.2 (C-

12), 77.3 (C-3), 71.6 (C-1), 61.8 (C-4'), 40.7 (C-2'), 39.7 (C-10), 36.1 (C-8), 34.9 (C-7), 33.1 (C-2), 29.6 (C-13), 28.7 (C-3'), 28.6 (C-11), 26.0 (SiC(*C*H_3)$_3$, 22.3 (C-5), 19.1 (C-6), 18.8 (C-14), 18.4 (Si*C*(CH_3)$_3$), -5.2 (Si(CH_3)$_2$). **HRMS** (ESI+) calc. for $[C_{24}H_{46}N_2NaO_6SSi]^+$ 541.2744 found 541.2739.

Acetonide of 5-Hydroxypentanoic pantetheine (S15)

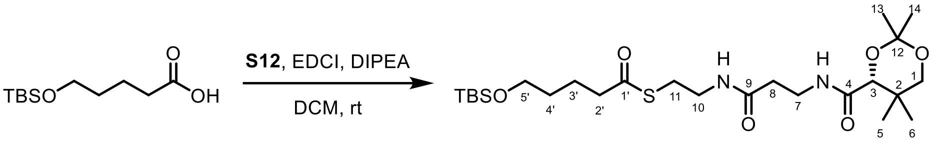

According to **GP03**, reaction of acid **S8** (219 mg, 0.942 mmol), EDCI (183 mg, 1.18 mmol), thiol **S12** (250 mg, 0.785 mmol) and DIPEA (0.27 mL, 1.57 mmol) gave the title compound **S15** (205 mg, 49%) as a viscous colourless oil. **δ_H (500 MHz, $CDCl_3$)** 7.01 (1H, t, *J* 6.5, NH), 6.05 (2H, t, *J* 5.0, NH), 4.08 (1H, s, 3-H), 3.68 (1 H, d, *J* 12.0, 1-*H*H), 3.61 (2H, t, *J* 6.0, 5′-H_2), 3.59 – 3.37 (4H, m, 7-H_2, 10-H_2), 3.28 (1H, d, *J* 12.0, 1-H*H*), 3.01 (2H, t, *J* 6.5, 11-H_2), 2.60 (2 H, t, *J* 7.5, 2′-H_2), 2.42 (2H, t, *J* 6.0, 8-H_2), 1.76 – 1.69 (2H, m, 3′-H_2), 1.55 – 1.50 (1H, m, 4′-H_2), 1.46 (3H, s, 13-H_3), 1.42 (3 H, s, 14-H_3), 1.04 (3H, s, 5-H_3), 0.97 (3H, s, 6-H_3), 0.89 (9H, s, SiC(CH_3)$_3$), 0.04 (6H, s, Si(CH_3)$_2$). **δ_C (125 MHz, $CDCl_3$)** 199.8 (C-1′), 171.3 (C-9), 170.3 (C-4), 99.2 (C-12), 77.3 (C-3), 71.6 (C-1), 62.7 (C-5′), 44.0 (C-2′), 39.8 (C-10), 36.1 (C-8), 34.9 (C-7), 33.1 (C-2), 32.0 (C-4′), 29.6 (C-13), 28.6 (C-11), 26.1 (SiC(*C*H_3)$_3$), 22.3 (C-5 and C-3′), 19.1 (C-6), 18.8 (C-14), 18.5 (Si*C*(CH_3)$_3$), -5.17 (Si(*C*H_3)$_2$. **ν_{max}/cm^{-1} (film)** 3313, 2952, 2929, 2858, 1658, 1652, 1567, 1098. **HRMS** (ESI+) calc. for $[C_{25}H_{49}N_2NaO_6SSi]^+$ 555.2895 found 555.2907.

Acetonide of 6-Hydroxyhexanoic pantetheine (S16)

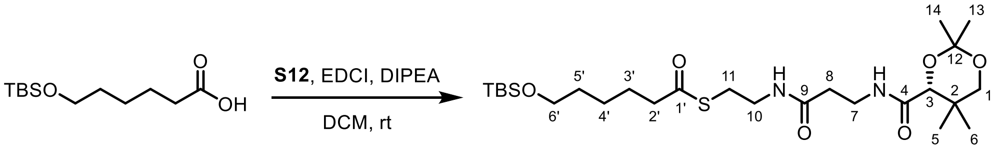

According to **GP03**, reaction of acid **S9** (75.0 mg, 0.31 mmol), EDCI (60.0 mg, 0.38 mmol), thiol **S12** (81 mg, 0.254 mmol) and DIPEA (90 µL, 0.51 mmol) gave the title compound **S16** (37.0 mg, 26.6 %) as a viscous colourless oil. **δ_H (400 MHz, $CDCl_3$)** 7.01 (1H, t, *J* 7.0, N-H), 6.13 (2H, t, *J* 6.5, N-H), 4.07 (1H, s, 3-H), 3.67 (1H, d, *J* 11.5, 1-*H*H), 3.59 (2H, t, *J* 6.5, 6'-H_2), 3.58 - 3.46 (2H, m, 7-H_2,), 3.43 (2H, t, *J* 6.5, 10-H_2), 3.27 (1H, d, *J* 11.5, 1-H*H*), 3.00 (2H, t, *J* 6.5, 11-H_2), 2.57 (2H, t, *J* 7.5, 2'-H_2), 2.41 (2H, t, *J* 6.0, 8-H_2), 1.67 (2H, p, *J* 7.5, 3'-H_2), 1.51 (2H, p, *J* 6.5, 5'-H_2), 1.45 (3H, s, 13-H_3), 1.41 (3H, s, 14-H_3), 1.41 – 1.32 (2H, m, 4'-H_2), 1.03 (3H, s, 5-H_3), 0.96 (3H, s, 6-H_3), 0.88 (9H, s, SiC(C*H_3*)$_3$), 0.03 (6H, s, Si(C*H_3*)$_2$). **δ_C (100 MHz, $CDCl_3$)** 199.8 (C-1'), 171.3 (C-4), 170.3 (C-9), 99.2 (C-12), 77.3 (C-3), 71.6 (C-1), 63.0 (C-6'), 44.3 (C-2'), 39.7 (C-10), 36.1 (C-8), 34.9 (C-7), 33.1 (C-2), 32.5 (C-5'), 29.6 (C-13), 28.6 (C-11), 26.1 (SiC(*C*H_3)$_3$), 25.6 (C-3'), 25.4 (C-4'), 22.3 (C-5), 19.1 (C-6), 18.9 (C-14), 18.5 (Si*C*(CH_3)$_3$), -5.1 (Si(*C*H_3)$_2$). **HRMS** (ESI+): calc. for $[C_{26}H_{50}N_2NaO_6SSi]^+$ 569.3057 found 569.3060.

Acetonide of 7-Hydroxyheptanoic pantetheine (S17)

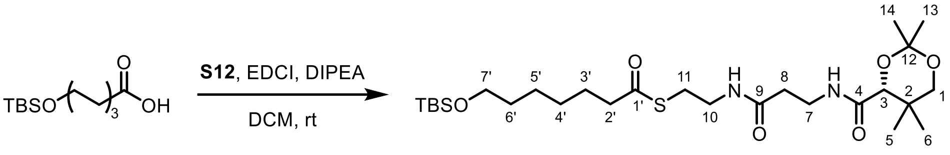

According to **GP03**, reaction of acid **S10** (67.0 mg, 0.304 mmol), EDCI (60.0 mg, 0.381 mmol), thiol **S12** (75.0 mg, 0.304 mmol) and DIPEA (90 µL, 0.508 mmol) gave the title compound **S17** (67.1 mg, 40.4 %) as viscous colourless oil. **δ_H (400 MHz, CDCl$_3$)** 7.02 (1H, t, *J* 6.5, N-H), 6.22 (1H, t, *J* 5.5, N-H), 4.06 (1H, s, 3-H), 3.67 (1H, d, *J* 11.5, 1-*H*H), 3.58 (2H, t, *J* 6.5, 7'-H$_2$), 3.58 – 3.45 (2H, m, 7-H$_2$), 3.49 – 3.31 (2H, m, 10-H$_2$), 3.26 (1H, d, *J* 11.5, 1-H*H*), 2.99 (2H, t, *J* 6.5, 11-H$_2$), 2.60 – 2.50 (2H, m, 2'-H$_2$), 2.41 (2H, t, *J* 6.0, 8-H$_2$), 1.65 (2H, ddt, *J* 12.0, 9.5, 6.0, 3'-H$_2$), 1.49 (2H, t, *J* 7.0, 6'-H$_2$), 1.45 (3H, s, 13-H$_3$), 1.40 (3H, s, 14-H$_3$), 1.37 – 1.27 (4H, m, 4'-H$_2$, 5'-H$_2$), 1.02 (3H, s, 5-H$_3$), 0.95 (3H, s, 6-H$_3$), 0.87 (9H, s, SiC(CH$_3$)$_3$), 0.03 (6H, s, Si(CH$_3$)$_2$). **δ_C (100 MHz, CDCl$_3$)** 199.90 (C-1'), 171.3 (C-9), 170.3 (C-4), 99.2 (C-12), 77.3 (C-3), 71.6 (C-1), 63.2 (C-7'), 44.2 (C-2'), 39.7 (C-10), 36.0 (C-8), 34.9 (C-7), 33.1 (C-2), 32.7 (C-6'), 29.6 (C-13), 28.9 (C-4'), 28.5 (C-11), 26.1 (SiC(*C*H$_3$)$_3$), 25.7 (C-3'), 25.6 (C-5'), 22.3 (C-5), 19.0 (C-6), 18.8 (C-14), 18.5 (Si*C*(CH$_3$)$_3$, -5.2 (Si(CH$_3$)$_2$). **HRMS** (ESI+) calc. for $[C_{27}H_{52}N_2NaO_6SSi]^+$ 583.3222 found 583.3208.

Acetonide of 9-Hydroxynonanoic pantetheine (S18)

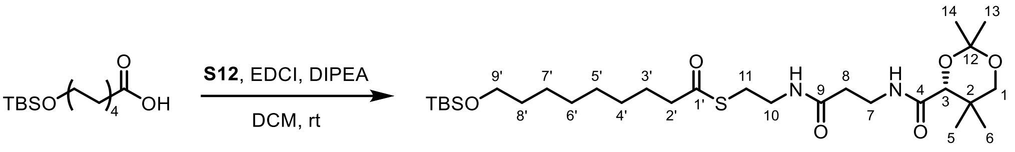

According to **GP03**, reaction of acid **S11** (67.9 mg, 0.24 mmol), EDCI (67.7 mg, 0.35 mmol), thiol **S12** (75.0 mg, 0.24 mmol) and DIPEA (82 µL, 0.47 mmol) gave the title compound **S18** (63 mg, 45.5 %) as a viscous colourless oil. **δ_H (400 MHz, CDCl$_3$)** 7.01 (1H, t, *J* 6.0, N-H), 6.12 (1H, t, *J* 6.0, N-H), 4.07 (1H, s, 3-H), 3.67 (1H, d, *J* 11.5, 1-*H*H), 3.58 (2H, t, *J* 6.5, 9'-H$_2$), 3.57 – 3.36 (4H, m, 7-H$_2$, 10-H$_2$), 3.27 (1H, d, *J* 11.5, 1-H*H*), 3.00 (2H, t, *J* 6.5, 11-H$_2$), 2.55 (2H, t, *J* 7.5, 2'-H$_2$), 2.41 (2H, t, *J* 6.0, 8-H$_2$), 1.64 (2H, p, *J* 7.0, 3'-H$_2$), 1.54 – 1.47 (2H, m, 8'-H$_2$), 1.45 (3H, s, 13-H$_3$), 1.41 (3H, s, 14-H$_3$), 1.29 (8H, m, 4 x C'H$_2$), 1.03 (3H, s, 5-H$_3$), 0.96 (3H, s, 6-H$_3$), 0.88 (9H, s, SiC(*C*H$_3$)$_3$), 0.04 (6H, s, Si(*C*H$_3$)$_2$). **δ_C (100 MHz, CDCl$_3$)** 199.9 (C-1'), 171.3 (C-9), 170.2 (C-4), 99.2 (C-12), 77.3 (C-3), 71.6 (C-1), 63.4 (C-9'), 44.3 (C-2'), 39.7 (C-10), 36.1 (C-8), 34.9 (C-7), 33.1 (C-2), 33.0 (C-8'), 29.6 (C-13), 29.3 (**CH$_2$**), 29.0 (**CH$_2$**), 28.6 (C-11), 26.1 SiC(*C*H$_3$)$_3$, 25.9 (**CH$_2$**), 25.7 (C-3'), 22.3 (C-5), 19.1 (C-6), 18.8 (C-14), 18.5 (Si*C*(CH$_3$)$_3$), -5.1 (Si(*C*H$_3$)$_2$). **HRMS (ESI+)** calc. for $[C_{29}H_{57}N_2O_6SSi]^+$ 589.3707 found 589.3702 $(M+H)^+$.

Acetonide of Crotonoyl pantetheine (S20)

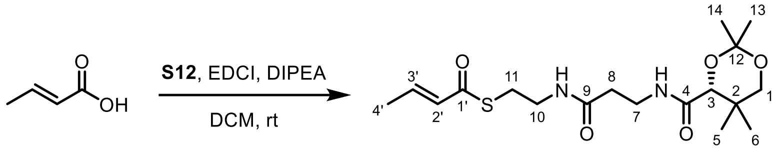

S19 **S20**

According to **GP03**, reaction of crotonic acid **S19** (35 mg, 0.38 mmol), EDCI (90 mg, 0.48 mmol), thiol (100 mg, 0.32 mmol) and DIPEA (110 µL, 0.64 mmol) gave the title compound **S20** (85 mg, 68.7 %) as a viscous pale-yellow oil. **δ_H (400 MHz, $CDCl_3$)** 7.05 – 6.99 (1H, m, NH), 6.94 (1H, dq, *J* 15.5, 7.0, 3'-H), 6.15 (1H, dq, *J* 15.5, 1.5, 2'-H), 6.14 – 6.08 (1H, m, NH), 4.07 (1H, s, 3-H), 3.68 (1H, d, *J* = 11.5, 1-*H*H), 3.62 – 3.39 (4H, m, 7-H_2, 10-H_2), 3.27 (1H, d, *J* = 11.5, 1-H*H*), 3.08 (2H, t, *J* = 6.5, 11-H_2), 2.41 (2H, t, *J* = 6.0, 8-H_2), 1.90 (3H, *J* = 7.0, 1.5, 4'-H_3), 1.46 (3H, s, 13-H_3), 1.42 (3H, s, 14-H_3), 1.04 (3H, s, 5-H_3), 0.97 (3H, s, 6-H_3). **δ_C (125 MHz, $CDCl_3$)** 190.2 (C-1'), 171.3 (C-9), 170.2 (C-4), 142.1 (C-3'), 130.0 (C-2'), 99.2 (C-12), 77.3 (C-3), 71.6 (C-1), 39.9 (C-10), 36.1 (C-8), 34.9 (C-7), 33.1 (C-2), 29.6 (C-13), 28.3 (C-11), 22.3 (C-5), 19.1 (C-6), 18.8 (C-14), 18.2 (C-4'). **HRMS** (ESI+): calc. for $[C_{18}H_{30}N_2NaO_5S]^+$ 409.1767 found 409.1784.

General Procedure 04 for Pantetheine Deprotection (GP04)

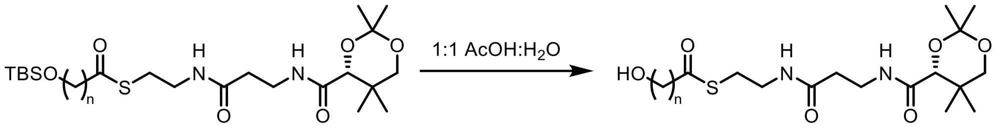

Thiol (1.0 equiv.) was added to a 1:1 solution of AcOH (0.1 M) and H_2O and stirred at rt overnight. The solvent was removed *in vacuo* and the crude material was purified (0-10% MeOH in CH_2Cl_2) to give the deprotected pantetheine substrates.

Crotonoyl pantetheine (4)

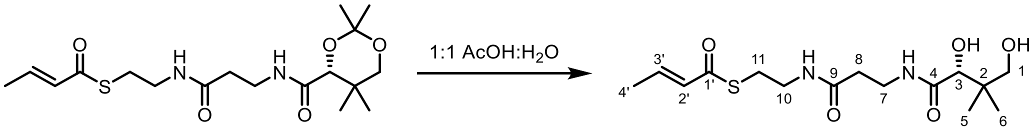

S20 **4**

According to **GP04**, reaction of protected pantetheine **S20** (85 mg, 0.22 mmol) in AcOH and H_2O gave the title compound **4** (43 mg, 56.4 %) as a viscous pale-yellow oil. **δ_H (500 MHz, CD_3OD)** 6.96 (1H, dq, *J* 15.5, 7.0, 3'-H), 6.21 (1H, dq, *J* 15.5, 1.5, 2'-H), 3.90 (1H, s, 3-H), 3.54 – 3.34 (6H, m, 1-H_2, 7-H_2, 10-H_2), 3.07 (2H, t, *J* 6.5, 11-H_2), 2.42 (2H, t, *J* 6.5, 8-H_2), 1.90 (3H, dd, *J* 7.0, 1.5, 4'-H_3), 0.93 (6H, s, 5-H_3, 6-H_3). **δ_C (125 MHz, CD_3OD)** δ 190.9 (C-1'), 176.1 (C-4), 173.9 (C-9), 142.9 (C-3'), 130.9 (C-2'), 77.3 (C-3), 70.4 (C-1), 40.4 (C-2), 40.3 (C-10), 36.4 (C-8), 36.3 (C-7), 28.9 (C-11), 21.3 (C-5), 20.9 (C-6), 18.0 (C-4'). **HRMS** (ESI+): calc. for $[C_{15}H_{26}N_2NaO_5S]^+$ 369.1455 found 369.1456.

All data are in accordance with the literature. [11]

5-Hydroxypentanoic pantetheine (5)

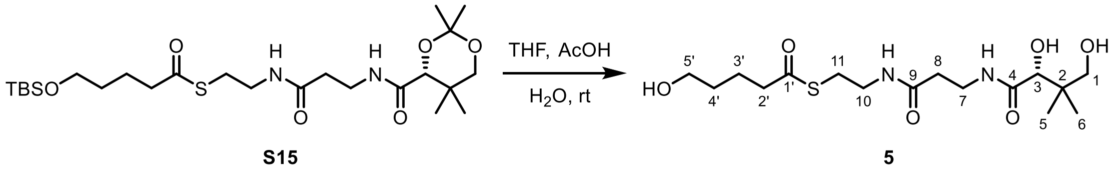

Thiol **S15** (231 mg, 0.434 mmol) was dissolved in a solution of THF (5 mL), $HCl_{(aq)}$ (1 M, 5 mL) and H_2O (5 mL) and stirred at rt for 1 h. EtOAc (10 mL) was added and the layers were separated. The aqueous layer was extracted with EtOAc (4 × 10 mL), and the combined organics were washed with $NaHCO_3$ (25 mL), dried over $MgSO_4$ and concentrated *in vacuo*. The crude material was purified by flash chromatography (0-10% MeOH in CH_2Cl_2) to give the title compound **5** (10 mg, 6%) as a viscous colourless oil. **δ_H (600 MHz, CD_3OD)** 3.90 (1H, s, 3-H), 3.56 (2H, t, *J* 6.5, 5'-H_2), 3.53 – 3.43 (3H, m, 7-H_2,1-*H*H), 3.40 (1H, d, *J* 11.0, 1-H*H*), 3.36 – 3.34 (2H, m, 10-H_2), 3.02 (2H, t, *J* 7.0, 11-H_2), 2.63 (2H, t, *J* 7.5, 2'-H_2), 2.42 (2H, t, *J* 7.0, 8-H_2), 1.76 – 1.70 (2H, m, 3'-H_2), 1.60 – 1.54 (2H, m, 4'-H_2), 0.93 (6H, s, 5-H_3, 6-H_3). **δ_C (150 MHz, CD_3OD)** 200.5 (C-1'), 176.0 (C-9), 173.9 (C-4), 77.3 (C-3), 70.3 (C-1), 62.4 (C-5'), 44.5 (C-2'), 40.4 (C-2), 40.1 (C-10), 36.4 (C-8), 36.3 (C-7), 32.7 (C-4'), 29.2 (C-11), 23.1 (C-3'), 21.3 (5-H_3), 20.9 (6-H_3). **ν_{max}/cm^{-1} (film)** 3324 (br), 2927, 2871, 1649, 1645, 1532. **HRMS (ESI+)** calc. for $[C_{16}H_{30}N_2NaO_6S]^+$ 401.1717 found 401.1731.

4-Hydroxybutanoic pantetheine (6)

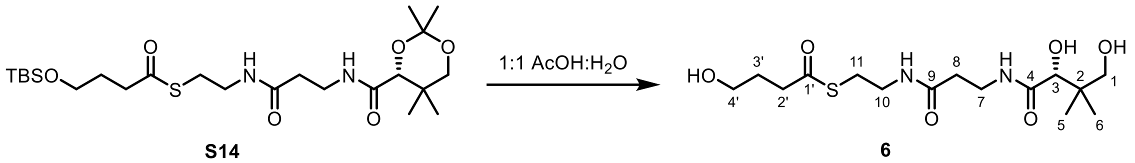

According to **GP04**, reaction of protected pantetheine **S14** (52 mg, 0.10 mmol) in AcOH and H_2O gave the title compound **6** (31 mg, 85 %) as a viscous colourless oil. **δ_H (500 MHz, CD_3OD)** 3.87 (1H, s, 3-H), 3.55 (2H, t, *J* 6.5, 4'-H_2), 3.52 – 3.39 (3H, m, 1-*H*H, 7-H_2), 3.37 (1H, d, *J* 11.0, 1-H*H*), 3.31 (2H, t, *J* 6.5, 10-H_2), 2.99 (2H, t, *J* 6.5, 11-H_2), 2.66 (2H, t, *J* 7.5, 2'-H_2), 2.39 (2H, t, *J* 6.5, 8-H_2), 1.83 (2H, m, 3'-H_2), 0.90 (6H, s, 5-H_3, 6-H_3). **δ_C (125 MHz, CD_3OD)** 200.3 (C-1'), 176.0 (C-4), 173.9 (C-9), 77.2 (C-3), 70.3 (C-1), 61.7 (C-4'), 41.3 (C-2'), 40.4 (C-2), 40.1 (C-10), 36.4 (C-8), 36.3 (C-7), 29.4 (C-3'), 29.2 (C-11), 21.3 (C-5), 20.9 (C-6). **HRMS** (ESI+) calc. for $[C_{15}H_{28}N_2NaO_6S]^+$ 387.1566 found 387.1568.

6-Hydroxyhexanoic pantetheine (7)

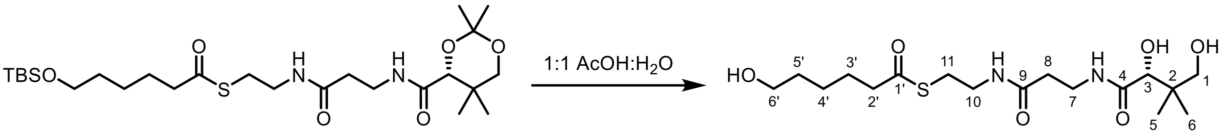

S16 **7**

According to **GP04**, reaction of protected pantetheine **S16** (37 mg, 0.07 mmol) in AcOH and H_2O gave the title compound **7** (18 mg, 68.4 %) as a viscous colourless oil. **δ_H (500 MHz, CD_3OD)** 3.90 (1H, s, 3-H), 3.55 (2H, t, *J* 6.5, 6'-H_2), 3.53 – 3.42 (3h, m, 1-*H*H, 7-H_2), 3.40 (1H, d, *J* = 11.0, 1-H*H*), 3.36 – 3.33 (2H, m, 10-H_2), 3.02 (2H, t, *J* 6.5, 11-H_2), 2.61 (2H, t, *J* 7.5, 2'-H), 2.42 (2H, t, *J* 6.5, 8-H_2), 1.69 (2H, p, *J* 7.5, 3'-H_2), 1.59 – 1.51 (2H, m, 5'-H_2), 1.44 – 1.37 (2H, m, 4'-H_2), 0.93 (6H, s, 5-H_3, 6-H_3). **δ_C (125 MHz, CD_3OD)** 200.6 (C-1), 176.1 (C-9), 173.9 (C-4f), 77.3 (C-3), 70.4 (C-1), 62.7 (C-6'), 44.8 (C-2'), 40.4 (C-2), 40.1 (C-10), 36.4 (C-8), 36.3 (C-7), 33.2 (C-5'), 29.2 (C-11), 26.5 (C-3'), 26.3 (C-4'), 21.3 (C-5), 20.9 (C-6). **HRMS** (ESI+): calculated for $[C_{17}H_{32}N_2NaO_6S]^+$ 415.1873 found 415.1879.

7-Hydroxyheptanoic pantetheine (8)

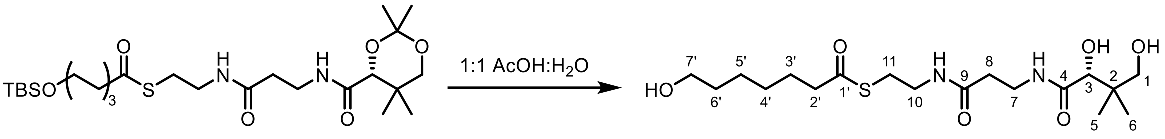

S17 **8**

According to **GP04**, reaction of protected pantetheine **S17** (67.1 mg, 0.12 mmol) in AcOH and H_2O gave the title compound **8** (24 mg, 49.2 %) as a viscous colourless oil. **δ_H (400 MHz, CD_3OD)** 3.88 (1H, s, 3-H), 3.53 (2H, t, *J* 6.5, 7'-H_2), 3.49 – 3.41 (3H, m, 1-*H*H, 8-H_2), 3.38 (1H, d, *J* 11.0, 1-H*H*), 3.32 (2H, m, 10-H_2), 2.99 (2H, t, *J* 6.5, 11-H_2), 2.58 (2H, t, *J* 7.5, 2'-H_2), 2.39 (2H, t, *J* 6.5, 8-H_2), 1.70 – 1.61 (2H, m, 3'-H_2), 1.51 (2H, m, 6'-H_2), 1.36 (4H, m, 4'-H_2, 5'-H_2), 0.91 (6H, s, 5-H_3, 6-H_3). **δ_C (100 MHz, CD_3OD)** 200.7 (C-1'), 176.1 (C-9), 173.9 (C-4), 77.3 (C-3), 70.3 (C-1), 62.8 (C-7'), 44.8 (C-2'), 40.4 (C-2), 40.1 (C-10), 36.4 (C-8), 36.3 (C-7), 33.4 (C-6'), 29.8 (C-4'), 29.1 (C-11), 26.7 (C-3'), 26.6 (C-5'), 21.3 (C-5), 20.9 (C-6). **HRMS (ESI+)** calc. for $[C_{18}H_{34}N_2NaO_6S]^+$ 429.2035 found 429.2029.

9-Hydroxynonanoic pantetheine (9)

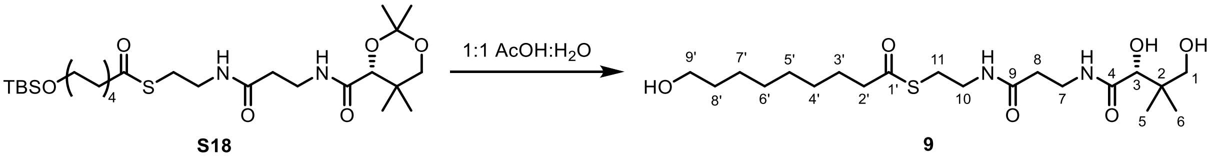

S18 **9**

According to **GP04**, reaction of protected pantetheine **S18** (63 mg, 0.11 mmol) in AcOH and H_2O gave the title compound **9** (9.0 mg, 18.9 %) as a viscous colourless oil. **δ_H (500 MHz, $CDCl_3$)** 7.45 (1H, t, *J* = 7.5,

NH), 6.61 (1H, t, *J* = 5.5, NH), 3.98 (1H, s, 3-H), 3.62 (2H, t, *J* = 6.5, 9'-H_2), 3.54 (2H, m, 7-H_2), 3.47 (2H, s, 1-H_2), 3.43 (1H, m, 10-*H*H), 3.37 (1H, m, 10-H*H*), 3.00 (2H, m, 11-H_2), 2.56 (2H, t, *J* = 7.5, 2'-H_2), 2.41 (2H, t, *J* = 6.0, 8-H_2), 1.64 (2H, m, 3'-H_2), 1.54 (2H, m, 8'-H_2), 1.37-1.27 (8H, m, 4 x C'-H_2), 0.99 (3H, s, 5-H_3), 0.91 (3H, s, 6-H_3). **δ_C (125 MHz, $CDCl_3$)** 200.5 (C-1'), 174.0 (C-4), 171.9 (C-9), 77.6 (C-3), 71.3 (C-1), 62.9 (C-9'), 44.2 (C-2'), 39.8 (C-10), 39.4 (C-2), 35.8 (C-8), 35.4 (C-7), 32.8 (C-8'), 29 (C-5', C-6'), 28.7 (C-4'), 28.4 (C-11), 25.7 (C-7'), 25.6 (C-3'), 21.6 (C-5), 20.6 (C-6). **HRMS** (ESI+) calc. for $[C_{20}H_{39}N_2O_6S]^+$ 435.2529 found 435.2531.

3. Materials and methods for molecular biology techniques

Reagents were purchased from Sigma-Aldrich, Thermo Fisher or Merck Millipore. *E. coli* competent cells were purchased from New England Biolabs (T7 Express and 5-α), Merck Millipore (Novagen BL21 (DE3)) or Agilent (ArcticExpress (DE3) RIL). All enzymes used were purchased from Thermo Fisher Scientific.

4. Plasmid generation, protein expression and purification.

Specific genes were amplified from *P. fluorescens* NCIMB 10856 genomic DNA with primers outlined in Table S1 and subcloned into either pOPINF (MacpB, MupB, MupD, MupE and MmpD_DH1) or pET28-a (MmpF & MmpF_C183A). pOPINF bears a N-terminal His_6 tag and a 3C protease cleavage site (LEVLFQGP), while pET28-a bears a N-terminal His_6 tag and a thrombin cleavage site (LVPRGS). The nucleotide sequence for MacpA, MmpB_ACP5, ACP6, ACP7, MmpB_KS, MmpB_DH1 and TacpB were synthesized and sub-cloned into a pET151-D/TOPO plasmid bearing an N-terminal His_6 tag and a tobacco etch virus (TEV) cleavage site (ENLYFQ) by Thermo Fisher. Both MupN and CoaA/CoaD/CoaE were expressed and purified as described previously [12].

Individual plasmids were transformed into *E. coli* T7 Express cells. Cultures were grown to OD_{600} = 0.7 in LB media (37 °C) supplemented with carbenicillin (100 μg/ml) or kanamycin (100 μg/ml) and induced (0.25-0.5 mM isopropyl β-D-1-thiogalactopyranoside) at 16 °C (16 hrs) before cell pellets were harvested by centrifugation (6000 rpm, 10 mins) and resuspended in buffer A (50 mM Tris-HCl, 500 mM NaCl, 10 % (v/v) glycerol, pH 8.0) before storing at -20 °C. For individual plasmids transformed into ArcticExpress RIL cells, individual colonies were incubated in LB media supplemented with carbenicillin (100 μg/ml) and gentamycin (20 μg/ml) for 16 hrs at 37 °C. Overnight cultures were supplemented into fresh LB media (1:10 dilution) and were grown to OD_{600} = 0.5-0.7 in LB media (30 °C) without antibiotic. Cultures were then induced (0.5 mM IPTG) at 10 °C for 24 hrs. Cell pellets were harvested (6000 rpm, 10 mins) and resuspended in buffer A before storing at -20 °C.

Harvested cells were sonicated and the soluble fraction was purified by immobilized metal affinity chromatography (IMAC) via a HiTrap 5 ml HP Ni column (GE Healthcare). Protein was eluted using a linear gradient from 6-100% of Buffer B (50 mM Tris-HCl, 500 mM NaCl, 10% (v/v) glycerol, 800 mM imidazole,

pH 8.0). Eluted protein was further purified by size exclusion chromatography (SEC) using either a HiPrep 26/60 Sephacryl S100 or S200 column (GE Healthcare) in Buffer C (25 mM Tris-HCl-, 150 mM NaCl, pH 7.5, 1 mM DTT) before protein concentration. MmpF, MmpF_C183 and MmpB_KS were purified by IMAC in Buffer A and B supplemented with 1mM TCEP, then by SEC in buffer A supplemented with 1 mM DTT. MacpA was cleaved overnight using in house TEV protease prior to SEC. Purified protein (50 μM) was analysed by analytical size exclusion chromatography using either a Superdex 75 10/300 or Superdex 200 increase 10/300 GL column (GE Healthcare) calibrated with molecular weight standards (GE Healthcare) [13].

For NMR studies, ^{15}N labelled protein was produced from cells grown to OD_{600} = 2.0 in LB media supplemented with carbenicillin (100 μg/ml) at 37 °C. Cells were then pelleted by centrifugation (6000 rpm, 10 mins), washed twice with sterile M9 media and then exchanged into M9 minimal media at a 4:1 volumetric ratio. Cells were supplemented with 1 gL^{-1} $^{15}NH_4Cl$, 0.5% (v/v) glycerol and 0.05% (w/v) glucose and induced with 0.5 mM IPTG, then harvested after 16 hrs at 16 °C. Cells were resuspended into buffer A and purified as described above for the unlabelled protein.

5. ESI-MS

Samples were desalted for ESI-MS analysis using a C_4 ZipTip (Merck) per the manufacturer's instructions. Denatured samples were analysed on a Synapt G2-Si (Waters) fitted with a TriVersa NanoMate (Advion) using the following parameters: sample cone, 10 V; capillary voltage 1.5 kV; trap collision energy, 10 V. The source as set to positive mode and spectra were acquired over 500-3000 m/z and analysed using MassLynx 4.1 software. For Ppant ejection assays, an appropriate charge state was isolated using the MSMS functionality. The transfer collision energy was increased until fragmentation was observed (typically 5 V to 20 V) and spectra were collected from 200-1000 m/z.

6 ESI-MS assay conditions

ACP upgrade reactions

100 μM MacpA, MacpB, MacpD, ACP5, ACP6 and ACP7 were upgraded with pantetheine substrates **5-8** or malonyl CoA as previously described[14] and desalted using a Zeba column (ThemoFisher), before used in the following assays.

Generation of 5-hydroxy-3-oxopentanoyl-ACP via MmpF

120 μM 3HP-MacpD was incubated with 20 μM MmpF and 20 μM malonyl-MacpA/B in buffer C at room temperature for 30 mins before monitoring by ESI-MS.

Generation of 3,5-dihydroxypentanoyl-MacpA/B via MmpF and MupD

37 μM 3HP-MacpD was incubated with 15 μM MmpF, 15 μM MupD, 1 μM NADPH and 50 μM malonyl-MacpA or malonyl-MacpB in buffer C at room temperature for 30 mins before monitoring by ESI-MS. For the negative control MmpF was substituted for MmpF_C183A mutant.

Dehydratase domain assays

37 μM 3HP-MacpD was incubated with 15 μM MmpF, 15 μM MupD, 1 mM NADPH, 50 μM malonyl-MacpB, 15 μM MupE, 1 mM NADH in buffer C at room temperature for 30 mins before ESI-MS.

MupE assays

80 μM crotonyl-MacpA or crotonyl-MacpB was incubated with 20 μM MupE and 200 μM NADH in buffer C at room temperature for up to 2 hrs before ESI-MS.

Generation of 3,7 dihydroxyheptanoyl-MacpA via MmpF and MupD

10 μM MmpF was incubated with 3.3 mM 5HP-pant, 15 μM MupD, 1 mM NADPH and 80 μM malonyl-MacpA or malonyl-MacpB in buffer C at room temperature for 3hrs before ESI-MS. For the negative control MmpF was substituted for the MmpF_C183A mutant. For assays testing the donor ACP 3.3 mM 5HP was substituted for 40 μM 5HP-MacpA or 5HP-MacpB and only 40 μM malonyl-MacpA was present.

MmpB_KS Substrate profile

40 μM MmpB_KS was incubated with 1 mM **4**, **5**, **6**, **7** or **9** in buffer C for 4 hrs at room temperature before ESI-MS.

Generation of 9-hydroxy β-ketononanoyl-ACP via MmpB_KS and malonyl-ACP5/6/7

20 μM MmpB_KS was incubated with 3.3 mM 7HH-pant and 80 μM malonyl-ACP5 (or malonyl-ACP6/7 counterparts) for up to 4 hrs in buffer C at room temperature before ESI-MS. For control experiments, malonyl-ACP5 was substituted for either 80 μM malonyl-MacpA or malonyl-MacpB. Alternatively 3.3 mM 7HH-pant was substituted for 3.3 mM 5HP-pant or 3.3 mM 6HH-pant to test the promiscuity of the MmpB_KS DCC reaction.

Generation of 9-hydroxy β-ketononanoyl-ACP5 via MmpB_KS and donor ACP species

20 μM MmpB_KS was incubated with 40 μM malonyl-ACP5 and either 40 μM 7HH-MacpA, 7HH-MacpB or 7HH-ACP5 in buffer C and incubated for 2 hrs before ESI-MS.

7. NMR parameters

All protein NMR experiments were acquired on a Bruker AVANCE III HD 700 MHz spectrometer equipped with a 1.7 mm triple-resonance cryogenically cooled detection probe. For titrations of ACP (ACP5, MacpA, MacpD, TacpB) with a partner protein (MmpB_KS, MmpF and MupB) ^{1}H-^{15}N HSQC spectra were recorded at pH 8.0 and either 288K (MmpB_KS, MmpF) or 298 K (MupB). The disappearance/reduction in intensity of chemical shifts correlated to a protein-protein interaction.

8. Phylogenetic tree analysis

Amino acid sequences of 663 KSs were extracted from 58 *trans*-AT PKS clusters with EryAII_KS3 and EryAIII_KS5 from the erythromycin *cis*-AT PKS chosen as outgroup KS domains. The sequences were aligned using the MUSCLE algorithm[15] with default settings and a Maximum-likelihood phylogenetic tree was computed using Mega-X with 100 bootstrap iterations using the LG +F substitution model, with Gamma distribution and gaps set to use all sites.[16] The tree was visualised using FigTree with clades manually assigned.

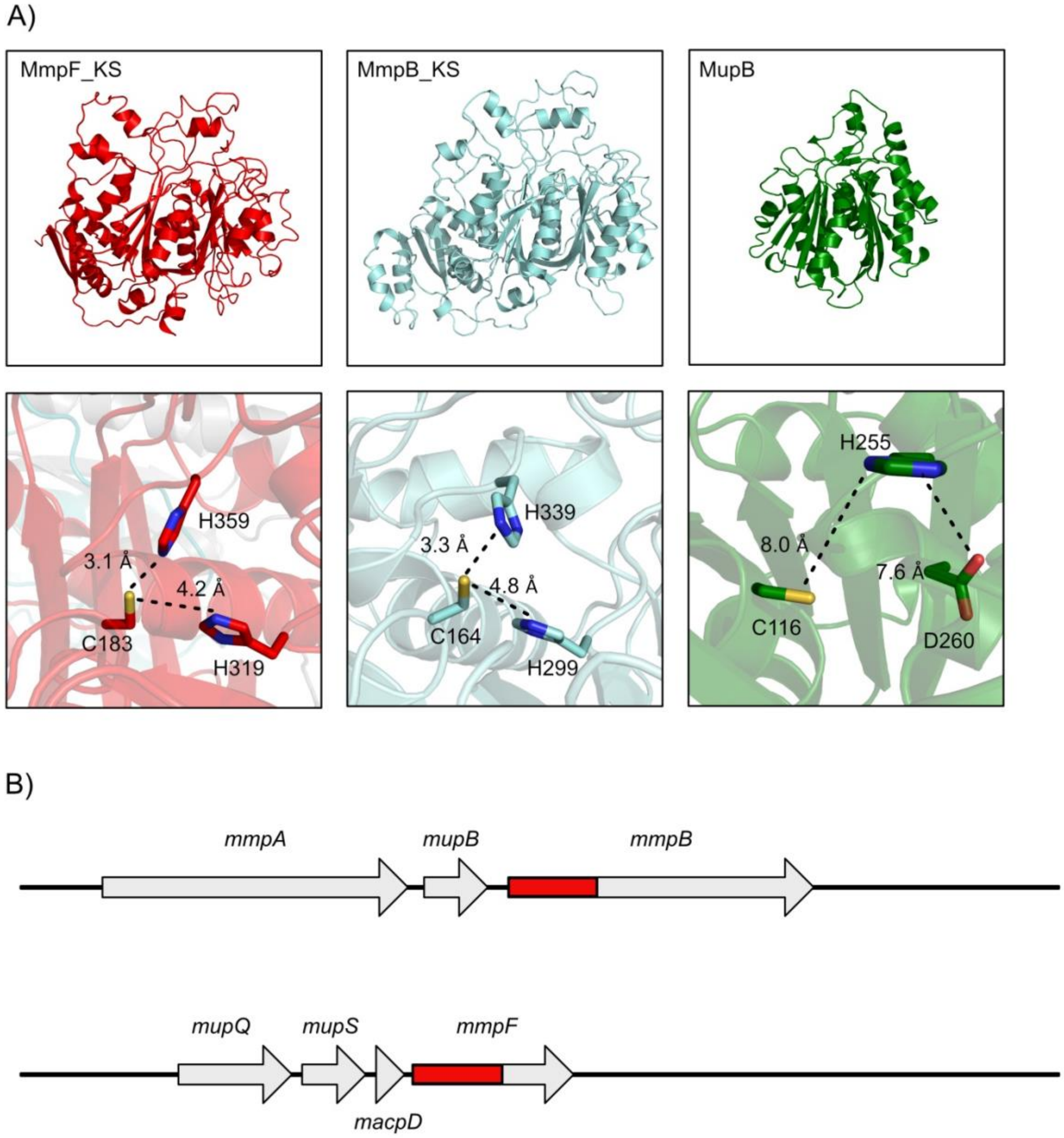

Supplementary Figure 1: Overview of the remaining unassigned KS domains within MmpF (red), MmpB (blue) and the trans acting module MupB (green) encoded within the mupirocin biosynthetic gene cluster. A) Cartoon overview of each KS domain illustrating conserved KAS I scaffold for both MmpF_KS and MmpB_KS, in comparison to MupB, which resembles KAS III enzymes. (Bottom panel): Active site triads of each enzyme, with distances to the catalytic cysteine highlighted (Å). Both MmpF and MmpB KS domains contain a Cys-His-His triad, whereas MupB contains a Cys-His-Asp triad. Each structure depicted is an *ab initio* homology model generated by I-TASSER.[17] B) Organisation of the unannotated KAS like enzymes in the mupirocin biosynthetic cluster. *mupB* is flanked by genes for the type I PKS modules *mmpA* and *mmpB*, while *mmpF* is encoded downstream of *mupQ*, *mupS* and *macpD*. KAS-like domains within the type I PKS are highlighted in red.

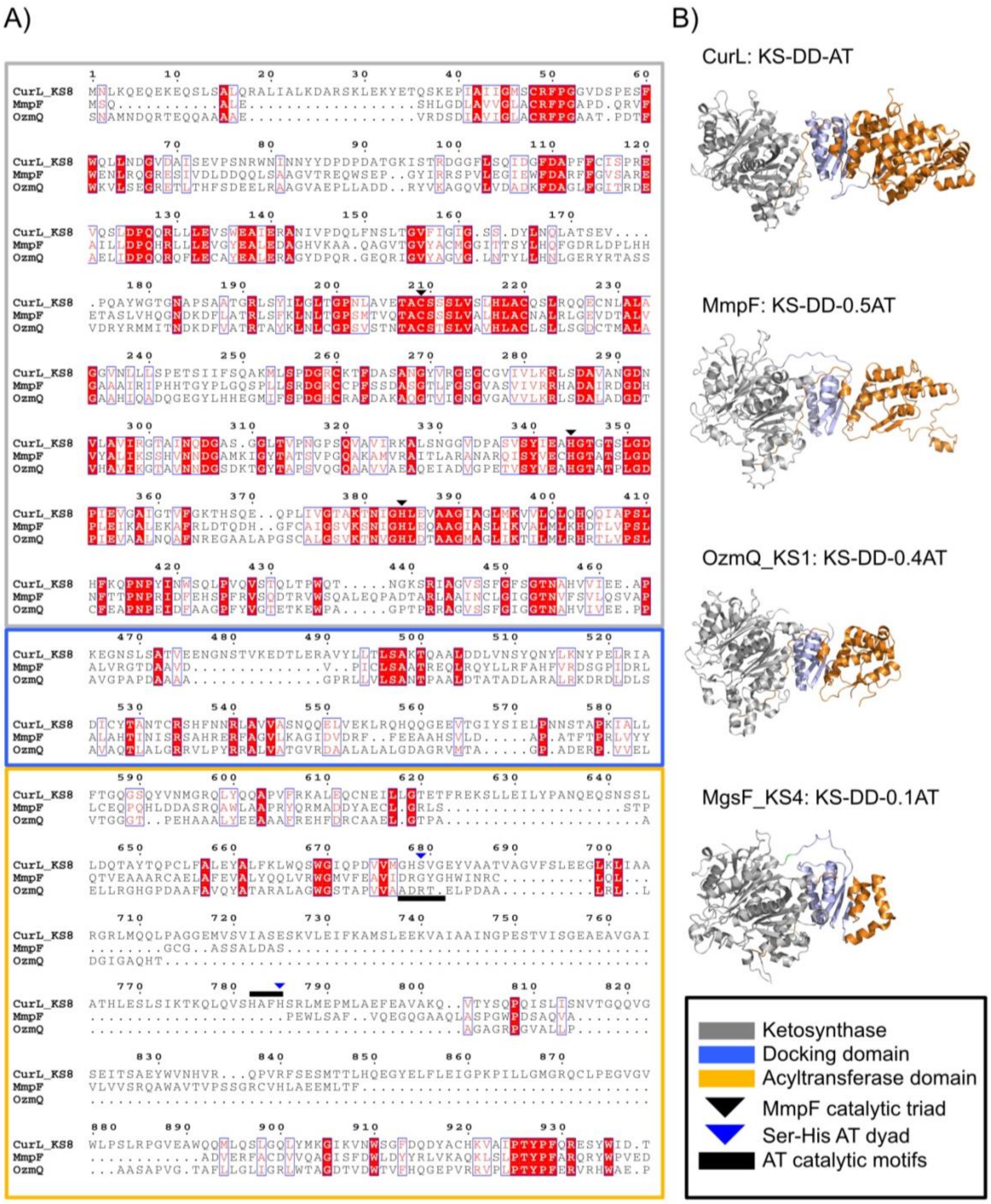

Supplementary figure 2: Bioinformatic analysis of MmpF. A) Multiple sequence alignment of MmpF with CurL_KS8 (KS-DD-AT, uniprot: F4Y424, 30.90% sequence identity) and OzmQ (KS-DD-0.4AT, uniprot: B2WW50, 38.72% sequence identity).[18] The catalytic triad within the active site of the KS domain with MmpF is highlighted (black arrows). Both the acyltransferase active site motif containing the critical serine (GxSxG) and the catalytic histidine whose motif dictates substrate specificity for malonyl CoA (HAFH) as observed for CurL_KS8 (black bar) is highlighted. Both residues of the Ser-His catalytic dyad (blue arrows) are not present within the di-domain of MmpF, suggesting the presence of a truncated, non-functional AT domain.[19] For clarity, each domain within the multiple sequence alignment: ketosynthase, docking domain and acyltransferase are highlighted in grey, blue and orange respectfully. B) Structural comparison of an *ab initio* homology model of MmpF [17] and the crystal structures of the KD-DD-AT homologues: CurL (PDB:4MZ0, RMSD: 0.7 Å), OzmQ (PDB:4OQJ, RMSD: 1.6 Å) and MgsF_KS4 (PDB:4ZDN, RMSD: 1.2 Å). KS domains are highlighted in grey, docking domain in blue and AT domain in orange. OzmQ and MgsF contain truncated AT domains and are inactive, when compared to the active full-length domain in CurL_KS8. As MmpF contains a truncated AT domain, this is presumed to be inactive. For clarity the sequence of MmpF was only compared to the sequence of the KS-AT di-domain encoded within the CurL module (KS-AT-OMT-KR-ACP) and the KS-DD-0.4 AT di-domain encoded within the OzmQ (KS-0.4AT-ACP) module.

A)

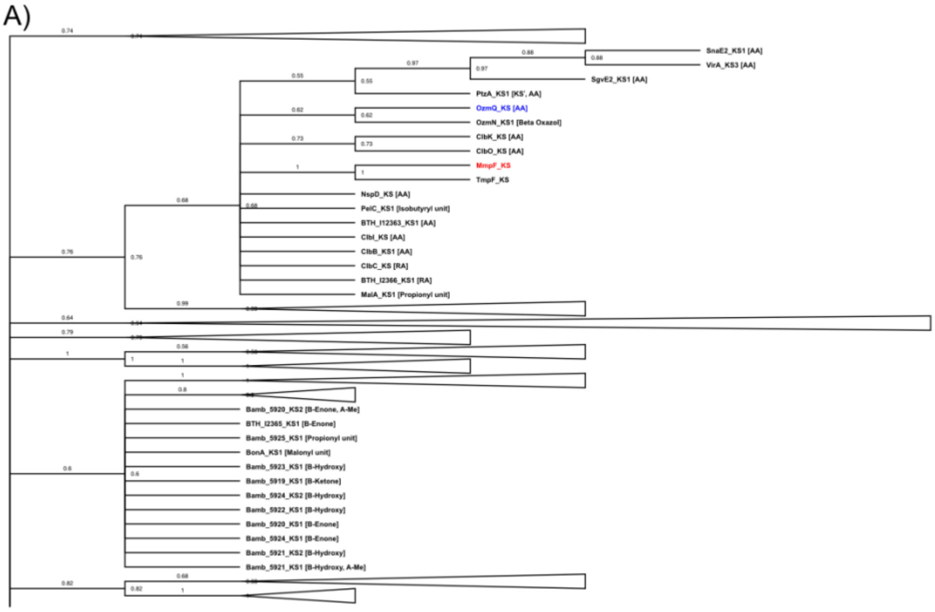
SnaE2_KS1 [AA]
VirA_KS3 [AA]
SgvE2_KS1 [AA]
PtzA_KS1 [KS', AA]
OzmQ_KS [AA]
OzmN_KS1 [Beta Oxazol]
ClbK_KS [AA]
ClbO_KS [AA]
MmpF_KS
TmpF_KS
NspD_KS [AA]
PelC_KS1 [Isobutyryl unit]
BTH_I12363_KS1 [AA]
ClbI_KS [AA]
ClbB_KS1 [AA]
ClbC_KS [RA]
BTH_I2366_KS1 [RA]
MalA_KS1 [Propionyl unit]
Bamb_5920_KS2 [B-Enone, A-Me]
BTH_I2365_KS1 [B-Enone]
Bamb_5925_KS1 [Propionyl unit]
BonA_KS1 [Malonyl unit]
Bamb_5923_KS1 [B-Hydroxy]
Bamb_5919_KS1 [B-Ketone]
Bamb_5924_KS2 [B-Hydroxy]
Bamb_5922_KS1 [B-Hydroxy]
Bamb_5920_KS1 [B-Enone]
Bamb_5924_KS1 [B-Enone]
Bamb_5921_KS2 [B-Hydroxy]
Bamb_5921_KS1 [B-Hydroxy, A-Me]

B)

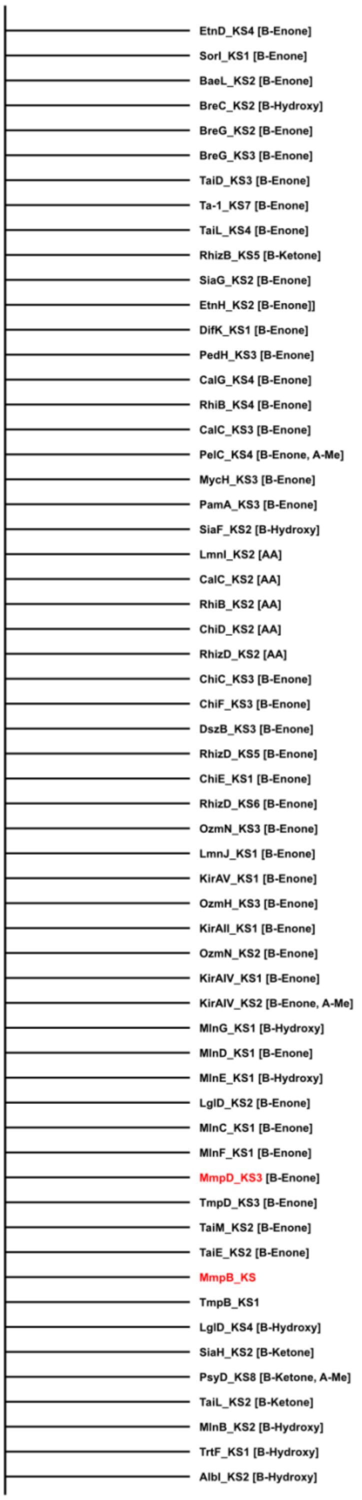

Supplementary Figure 3: Phylogram of 663 full-length *trans*-AT PKS KS domains and assignment of clades to biochemical transformation. Maximum likelihood phylogenetic tree of *trans*-AT PKS KS sequences from *trans*-AT PKS for which the metabolite has been identified. Two *cis*-AT PKS KS domains from the erythromycin PKS (EryAII_KS1 and EryAIII_KS1) were used as the outgroup. A) Cross-section of clades that contains MmpF and OzmQ. B) Cross-section of clades that contains MmpB_KS. Bootstrap values are represented as numerals at the nodes. KS's corresponding to the mupirocin pathway are highlighted in red, KS domains with a truncated AT domain: OzmQ and MgsF_KS4 are highlighted in blue. Key: AA: Aminoacyl, A-DiMe: Dimethyl α-branch, A-Me: Methyl α-branch, B-Enone: β-enone group, B-hydroxy: β-hydroxyl group, B-Ketone: β-ketone group, RA: Reduced β-acyl chain.

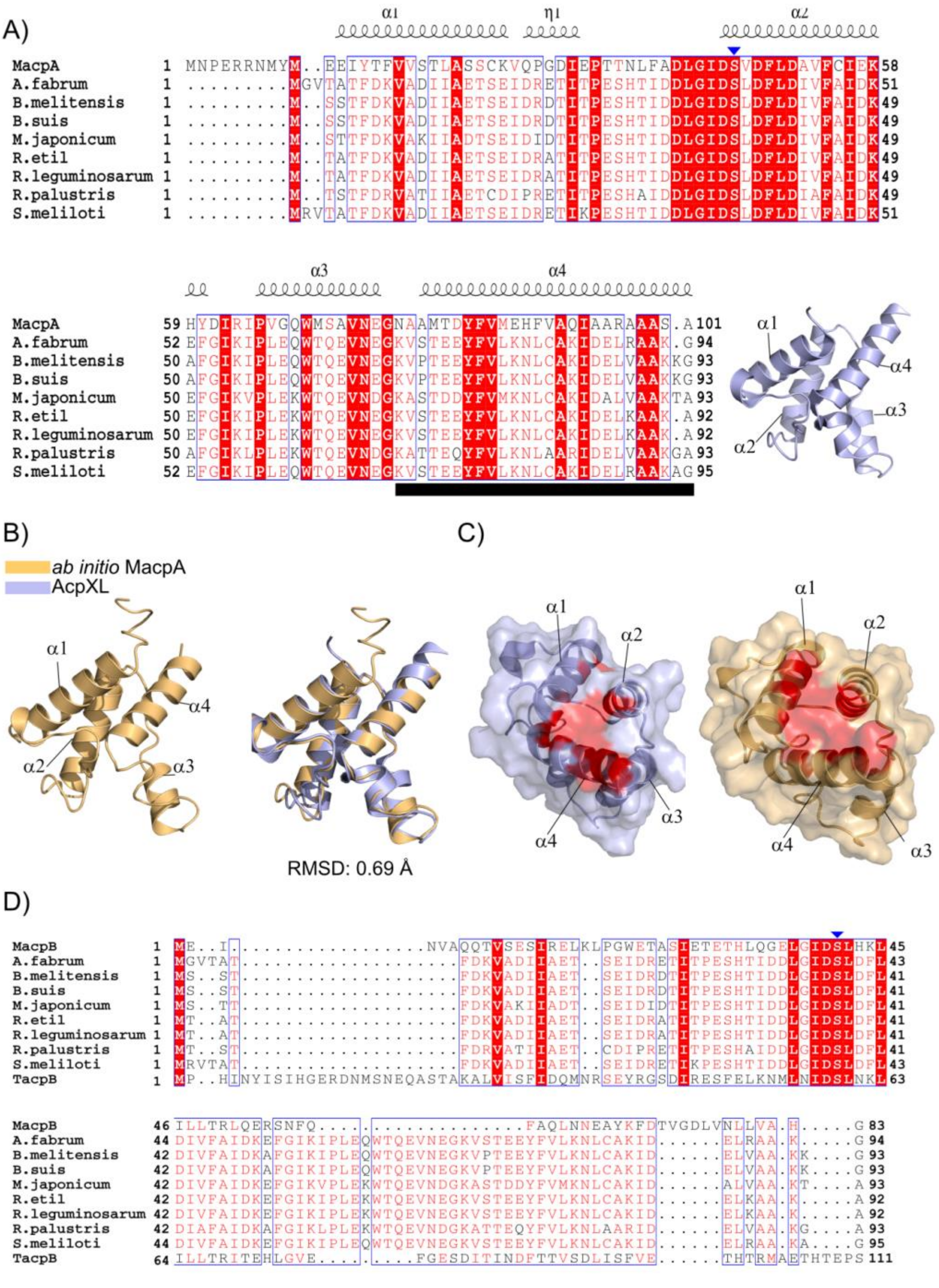

Supplementary Figure 4. Sequence alignment of mupirocin ACPs. A) Multiple sequence alignment of MacpA (Uniprot: Q8RL70) with AcpXL from *Agrobacterium fabrum* (40.0% identity), *Brucella melitensis* (38.5% identity), *Brucella suis biovar* (39.0% identity), *Mesorhizobium japonicum* (39.6 % identity), *Rhizobium etil* (38.0 % identity), *Rhizobium leguminosarum* (38.0% identity), *Rhodopseudomonas palustris* (37.0% identity) and *Shinorhizobium meliloti* (38.3%) identity). The active site serine is highlighted (blue arrow) and secondary structure elements from *R.palustris* ACPXL (PDB:2KW2, blue, inset) highlighted. B) *Ab initio* homology model of MacpA [17] (orange) and structural similarity with *R.palustris* ACPXL (blue), RMSD: 0.69 Å. C) Top view of *R. palustris* ACPXL with hydrophobic amino acids that form the hydrophobic cavity highlighted in red. Several amino acids from the extra helix (α4) are critical in forming this cavity. Comparison of this structure to the *ab initio* homology model of MacpA indicates an overall conservation of hydrophobic amino acids (red) within the extra helix (α4). D) Multiple sequence alignment of MacpB (Uniprot:Q8RL67) with AxpXL sequences from *A. fabrum* (27.2% identity), *B. melitensis* (25.3% identity), *B. suis* (25.3% identity), *M.japonicum* (20.3% identity), *R. leguminosarum* (28.8% identity), *R. palustris* (31.9% identity), *S. meliloti* (26.3% identity) and *Pseudoalteromonas* sp. SANK 73390 TacpB (31.6% identity). The active site serine is highlighted (blue arrow) and the black bar indicates the C-terminal α-helical extension.

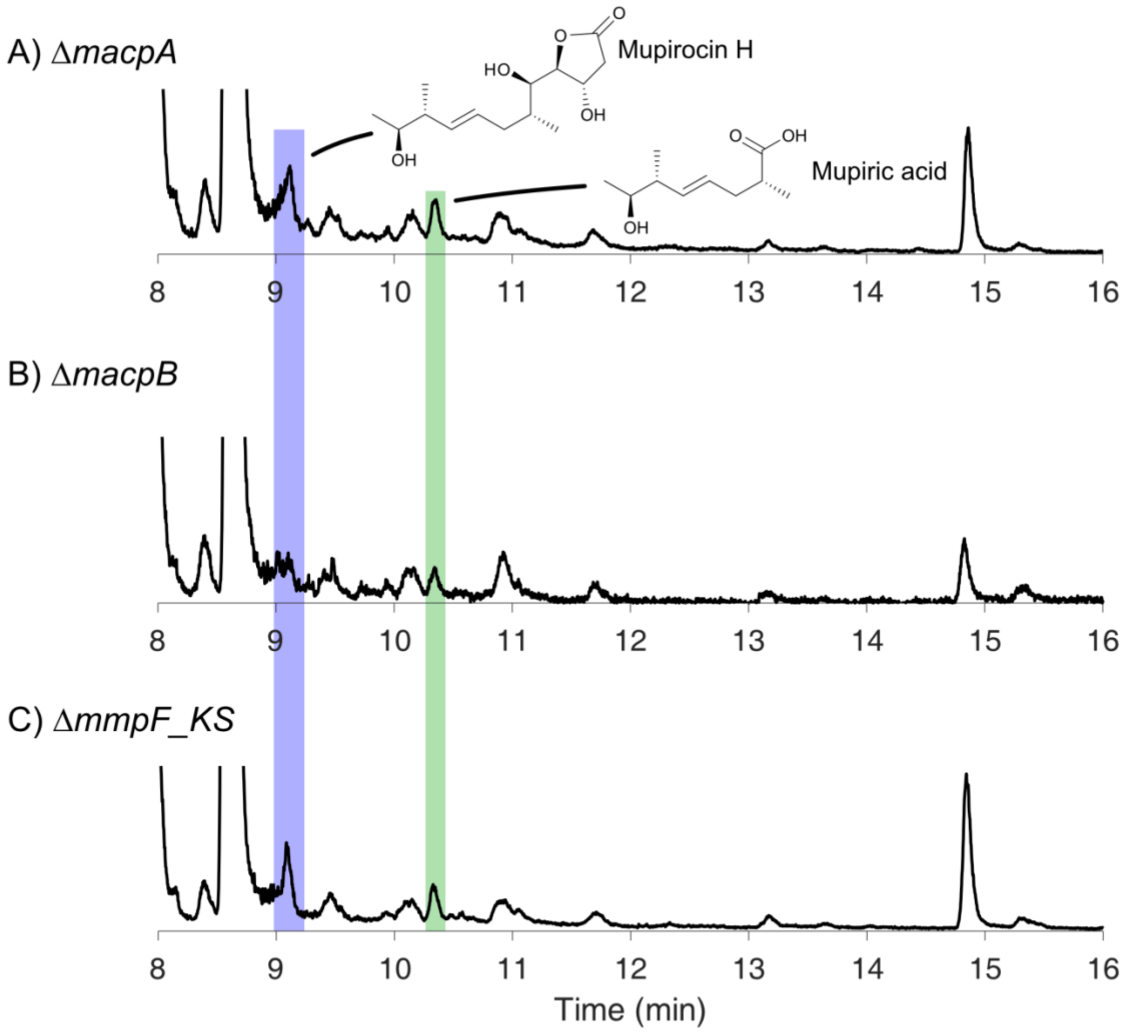

Supplementary Figure 5. Metabolites isolated from extracts of mutant strains of *Pseudomonas fluorescens* NCIMB 10585. A) Δ*macpA* mutant. B) Δ*macpB* mutant. C) Δ*mmpF_KS* mutant. Each mutant showed the presence of mupirocin H (blue), mupiric acid (green) and the abolishment of PA-A production.

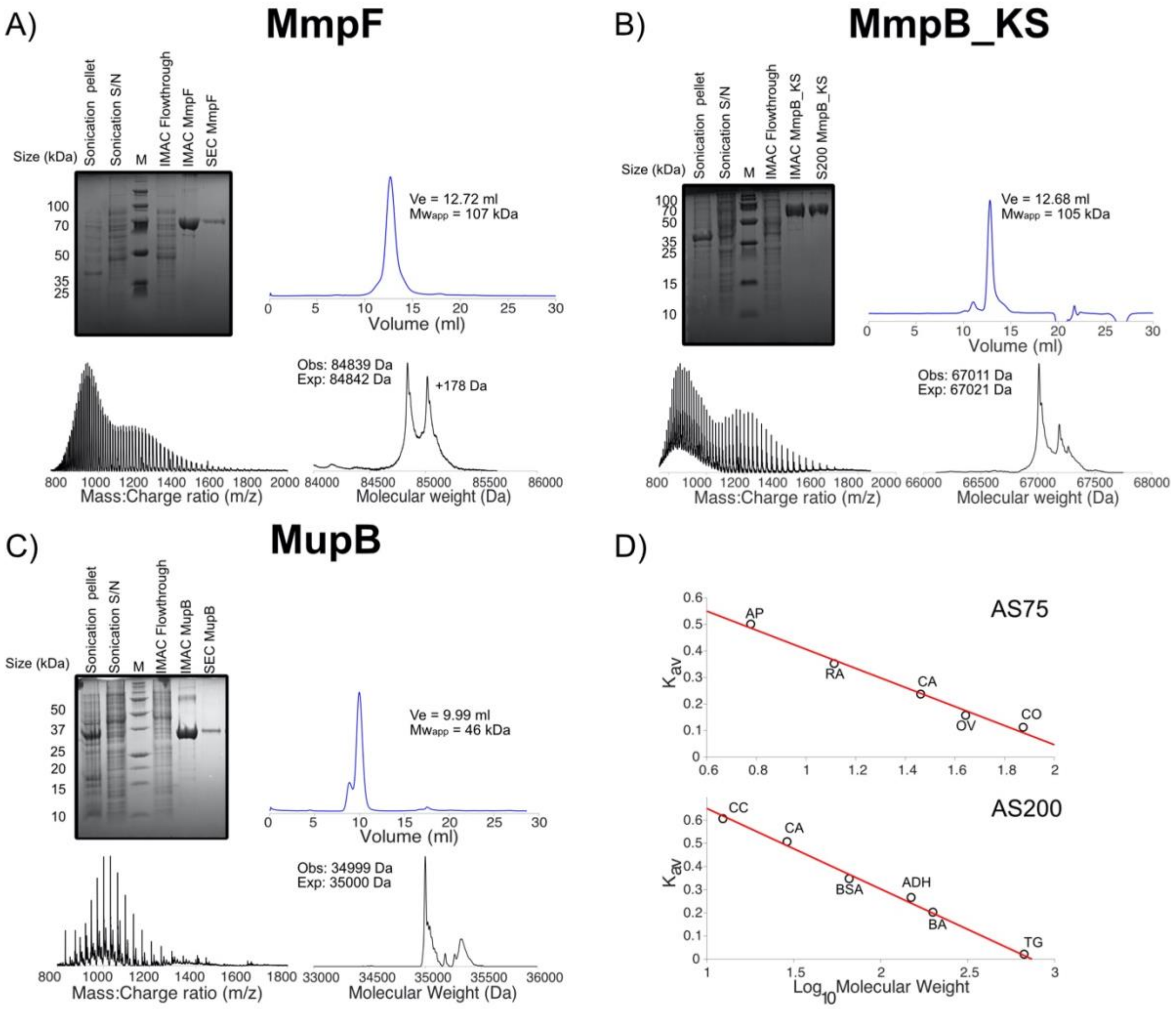

Supplementary Figure 6: Purification and characterisation of all KS domains used within this study. A) Purification of MmpF. SDS-PAGE following the purification of MmpF after sonication to isolation via IMAC and SEC. Analytical SEC showing MmpF eluting as a single, likely dimeric, species. ESI-MS of denatured MmpF yielding monomeric MmpF: Expected molecular weight: 84,842 Da. An adduct at +178 Da corresponding to the phosphogluconylation of the His_6 tag is highlighted. B) Purification of MmpB_KS. SDS-PAGE following the purification of MmpB_KS after sonication and isolation via IMAC and SEC. Analytical SEC showing elution of MmpB_KS as a single oligomeric species. ESI-MS of denatured MmpB_KS yielding monomeric MmpB_KS: Expected molecular weight: 67,021 Da. C) Purification of MupB. SDS-PAGE following the purification of MupB after sonication to isolation via IMAC and SEC (likely dimer). ESI-MS of denatured MupB yielding monomeric MupB: Expected molecular weight: 35,000 Da. D) Calibration curve for the Analytical S75 and Analytical S200 column using the following calibrants: Aprotinin (AP), Ribonuclease A (RA), Carbonic Anhydrase (CA), Ovalbumin (OV), Conalbumin (CO), Cytochrome C (CC), Bovine Serum Albumin (BSA), Alcohol Dehydrogenase (ADH), β-Amylase (BA) and Thyroglobulin (TG).

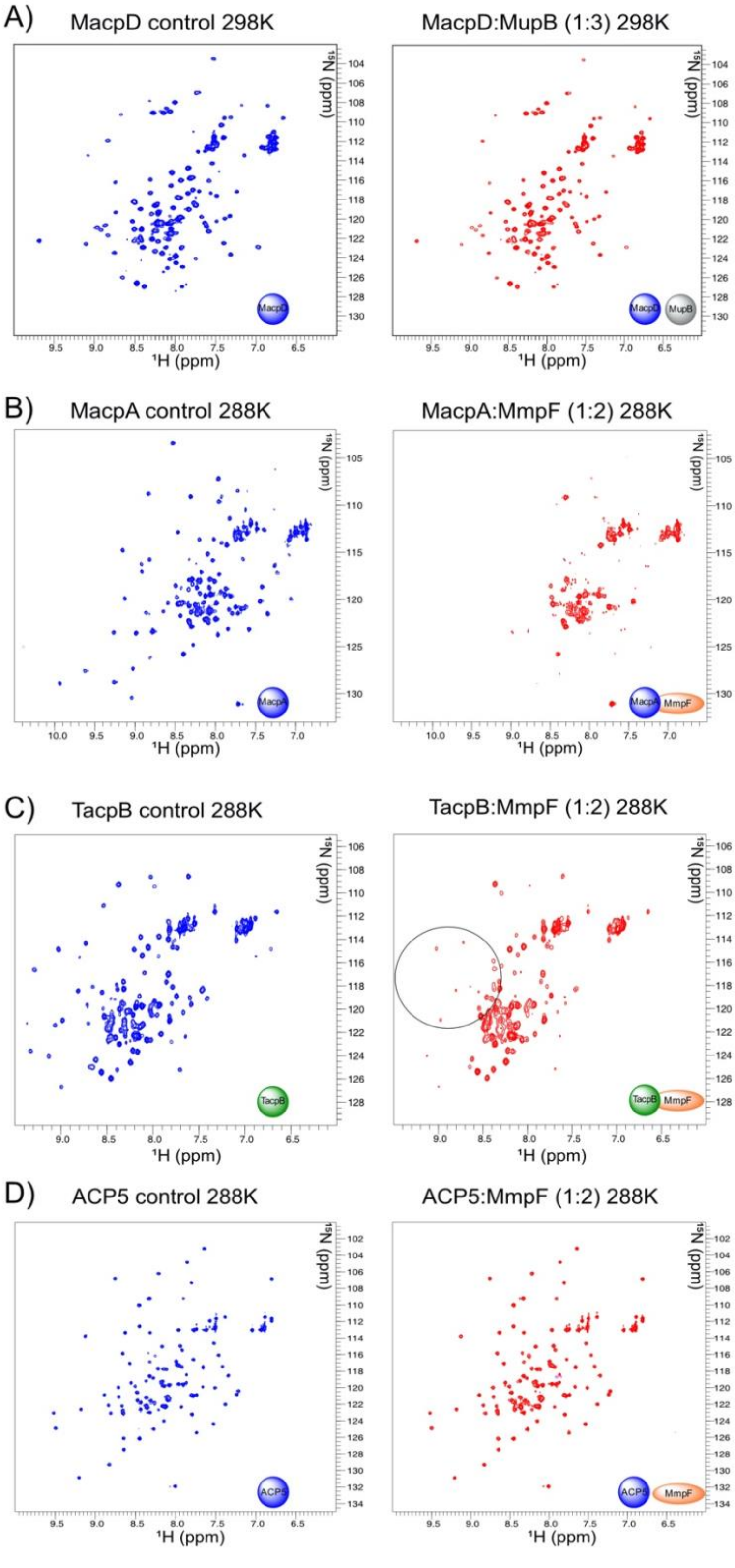

Supplementary Figure 7: ^{1}H-^{15}N HSQC experiments to determine protein-protein interactions between MupB and MacpD and then MmpF and MacpA/TacpB/ACP5. A) Control spectrum of *apo* MacpD at 298 K/pH 8.0 (blue) and in the presence of three equivalents of MupB (red). No discernible chemical shift perturbations (CSPs) or broadening of residues observed. B) Control spectrum of *apo* MacpA at 288 K/pH 8.0 (blue) and in the presence of two equivalents of MmpF (red). C) Control spectrum of *apo* TacpB at 288 K/pH 8.0 (blue) and in the presence of two equivalents of MmpF (red). Moderate resonance broadening or CSPs observed with all resonances remaining visible. D) Control spectrum of *apo* ACP5 at 288 K/pH 8.0 (blue) and in the presence of two equivalents of MmpF (red). No resonance broadening or CSPs were observed.

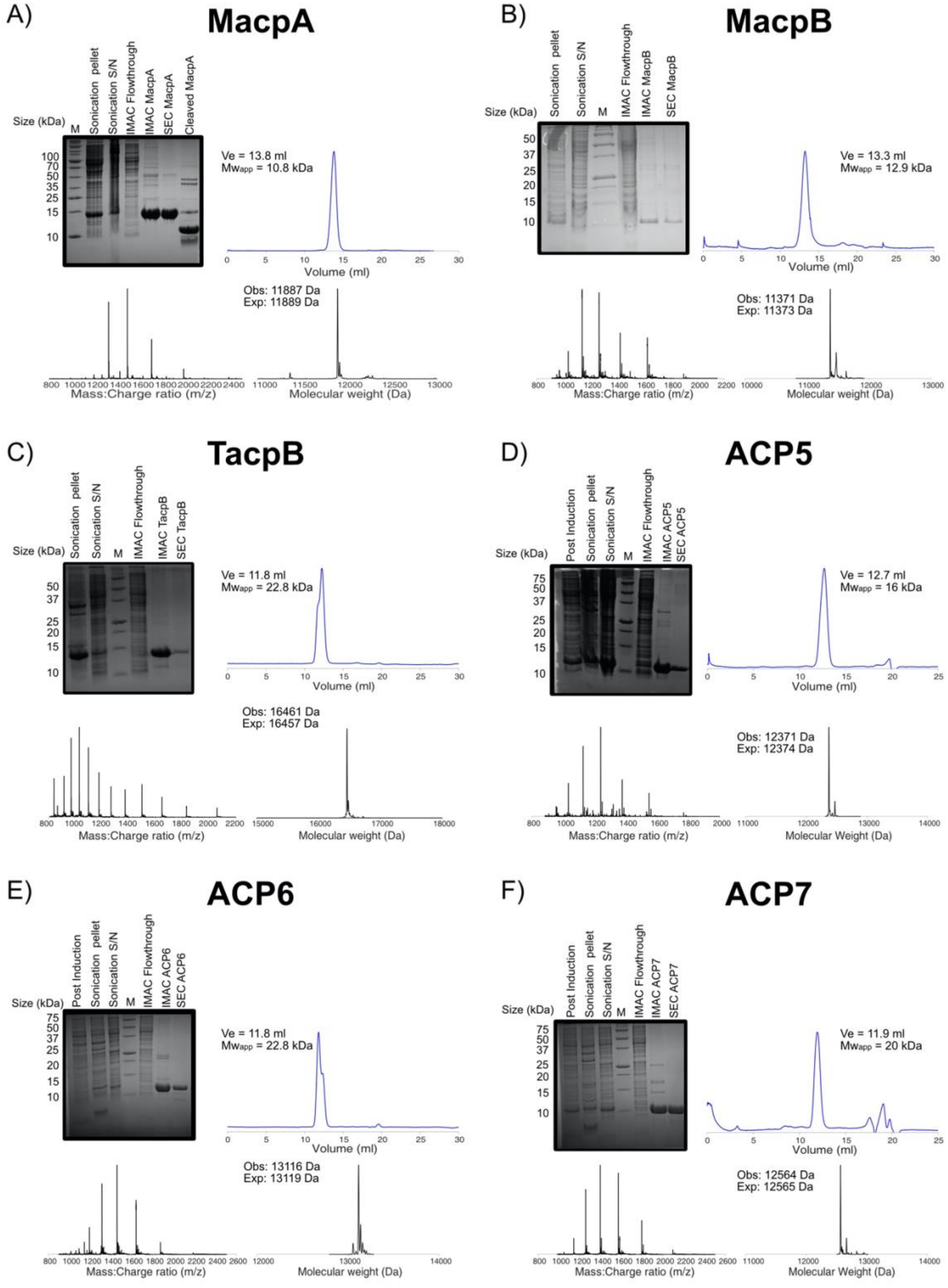

Supplementary Figure 8: Purification followed by SDS-PAGE and characterisation of all ACPs used within this study. A) Purification of MacpA. SDS-PAGE following the purification of MacpA after sonication to isolation via IMAC and SEC before cleavage of the His_6 tag. Analytical SEC showing MacpA eluting as a single oligomeric species. ESI-MS of denatured MacpA yielding monomeric MacpA. B) MacpB. Including analytical SEC showing elution of MacpB.6His as a single species. C) TacpB. Including analytical SEC showing elution of TacpB.6His as a single species. D) ACP5 E) ACP6 and F) ACP7. All Including analytical SEC showing elution as a single species and correct masses by ESI-MS.

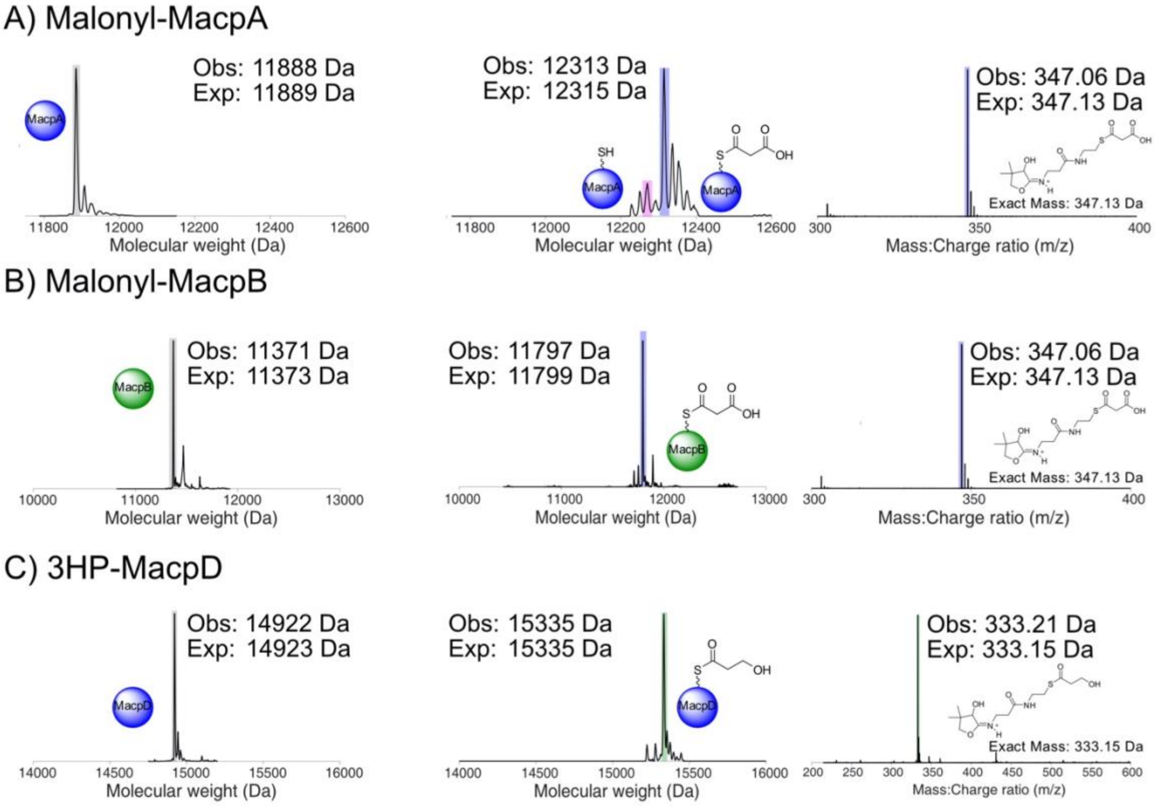

Supplementary Figure 9: Generation of ACP-loaded substrates. A) Deconvoluted spectra of *apo* MacpA (left) (obs: 11,888 Da, exp: 11,889 Da) and malonyl-MacpA (right) (obs: 12,311 Da, exp: 12,316 Da) and the Ppant ejection for this species. B) Deconvoluted spectra of *apo* MacpB (left) (obs: 11,371 Da, exp: 11,371 Da) and malonyl-MacpB (right) (obs: 11,796 Da, exp: 11,798 Da) and the Ppant ejection for this species. C) Deconvoluted spectra of *apo* MacpD (left) (obs: 14,922 Da, exp: 14,923 Da) and 3HP-MacpD (right) (obs: 15,337 Da, exp: 15,335 Da) and the Ppant ejection for this species (obs: 333.26 Da, exp: 333.15 Da). Species are colour coded as *apo*-ACP (grey), *holo*-ACP (pink), malonyl-ACP (blue) and 3HP-MacpD (green).

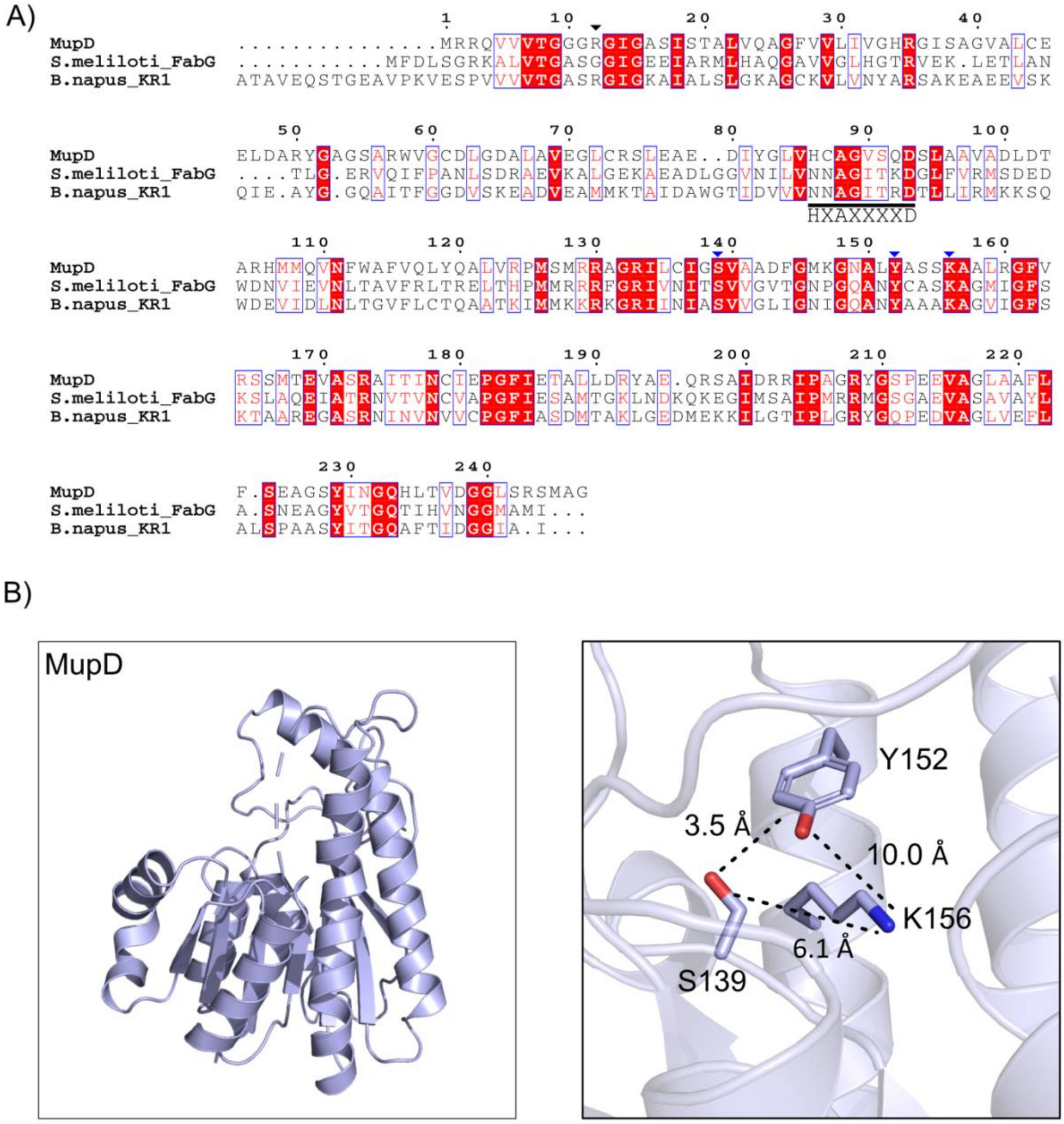

Supplementary Figure 10. Bioinformatic analysis of MupD. A) Multiple sequence alignment between MupE and ketoreductase homologs from *Shinorhizobium meliloti* (FabG, Uniprot:A0A222J7X4, 35.29% sequence identity) and *Brassica napus* (KR, Uniprot:Q93X62, 36.10% sequence identity). The arginine that dictates NAD(P)H specificity and residues that form the active site are highlighted by black and blue arrows respectively. The HXAXXXXD motif that dictates stereochemistry is also highlighted. B) *Ab initio* homology of MupD, with the active site triad (S139, Y152, K156) is highlighted.

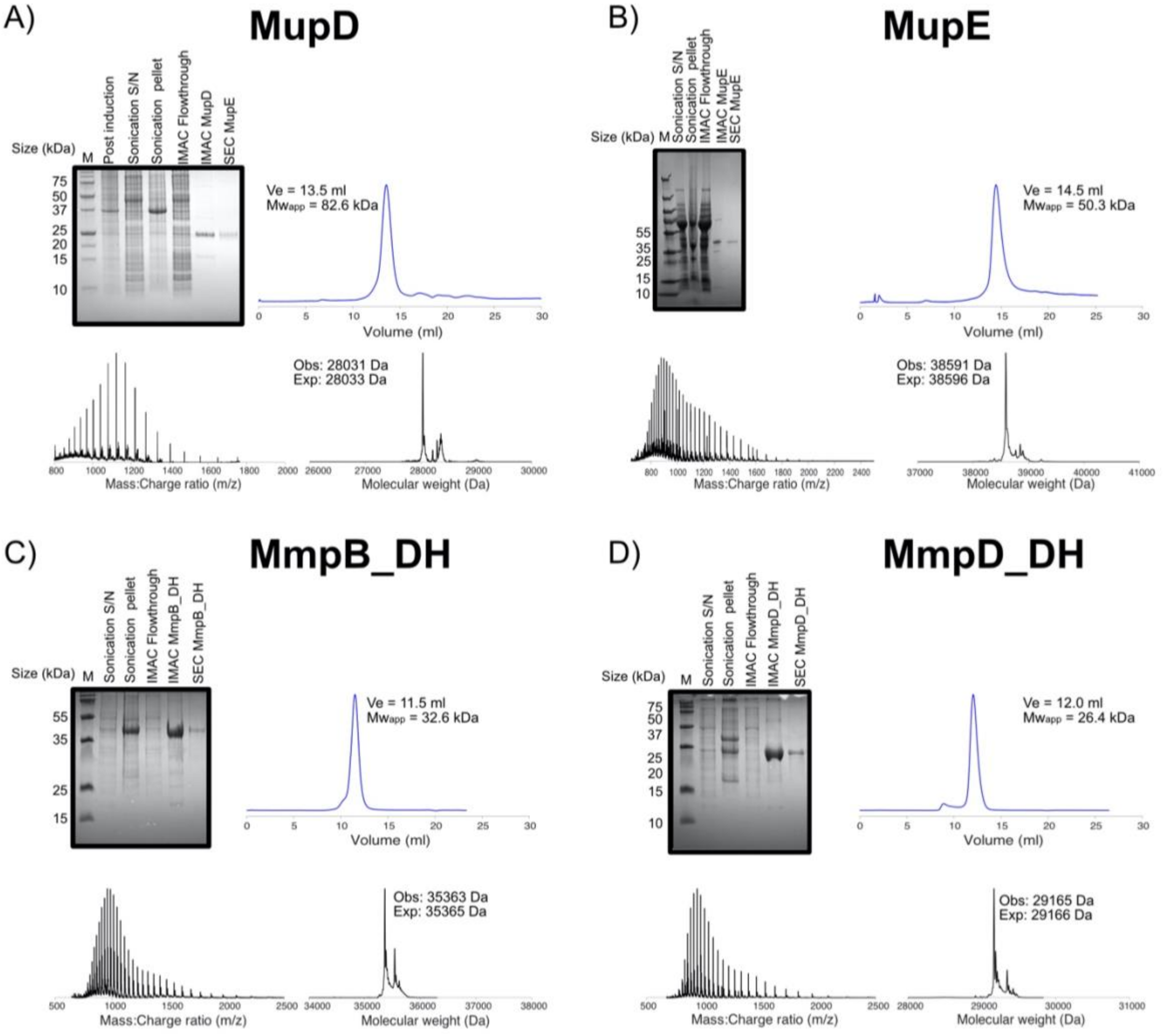

Supplementary Figure 11: Purification of enzymes involved in processing downstream of MmpF. A) Purification of MupD. SDS-PAGE following the purification of MupD.6His after sonication to isolation via IMAC and SEC. Analytical SEC showing MupD.6His eluting as a single oligomeric species. ESI-MS of denatured MupD.6His yielding monomeric MupD.6His: Expected molecular weight: 28,033 Da. B) Purification of MupE. SDS-PAGE following the purification of MupE.6His after sonication and isolation via IMAC and SEC. Analytical SEC showing elution of MupE.6His as a single species. ESI-MS of denatured MupE.6His yielding monomeric MupE.6His: Expected molecular weight: 38,596 Da. C) Purification of MmpB_DH. SDS-PAGE following the purification of MmpB_DH.6His after sonication to isolation via IMAC and SEC. Analytical SEC showing elution of MmpB_DH.6His as a single species. ESI-MS of denatured MmpB_DH.6His yielding monomeric MmpB_DH.6His: Expected molecular weight: 35,365 Da. D) Purification of MmpD_DH. SDS-PAGE following the purification of MmpD_DH.6His after sonication and isolation via IMAC and SEC. Analytical SEC showing elution of MmpD_DH.6His as a single oligomeric species. ESI-MS of denatured MmpD_DH.6His yielding monomeric MmpD_DH.6His: Expected molecular weight: 29,165 Da.

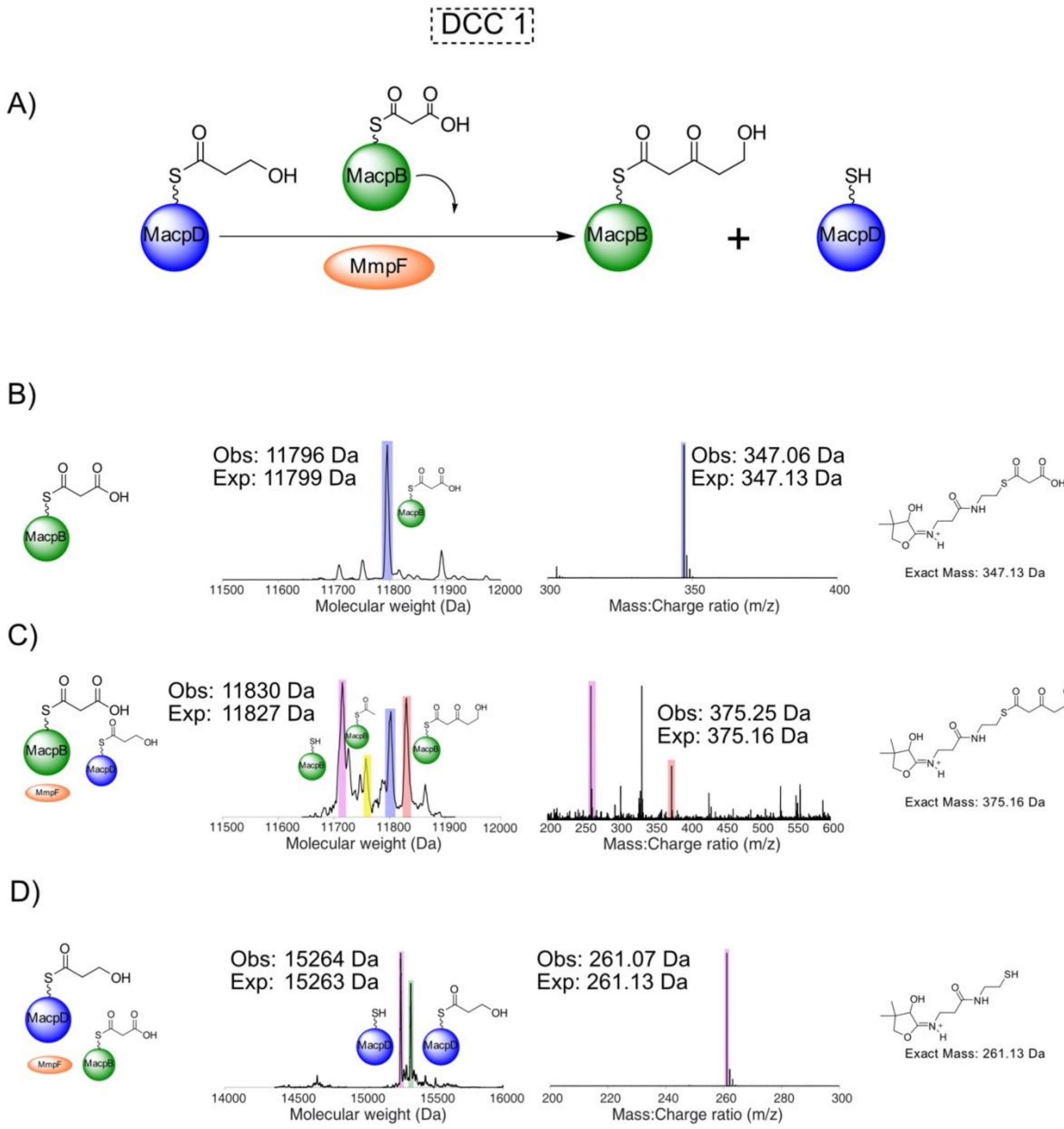

Supplementary Figure 12. Condensation reaction between 3HP-MacpD, MmpF and Malonyl-MacpB. A) Proposed reaction scheme for the decarboxylative Claisen condensation of 3HP-MacpD by MmpF and Malonyl-MacpB to produce β-keto hydroxypentanoyl MacpB carbon intermediate attached to MacpB. B) Deconvoluted spectra and corresponding Ppant ejection for Malonyl-MacpB only with observed and expected mass indicated (blue). C) Deconvoluted spectra and corresponding Ppant ejection for MacpB when incubated with MmpF and 3HP-MacpD to produce species (red). D) Deconvoluted spectra and corresponding Ppant ejection for 3HP-MacpD (green). E) Deconvoluted spectra and corresponding Ppant ejection for MacpD when incubated with MmpF and Malonyl-MacpB where *holo*-MacpD is produced (pink). For each panel the observed and expected mass is highlighted.

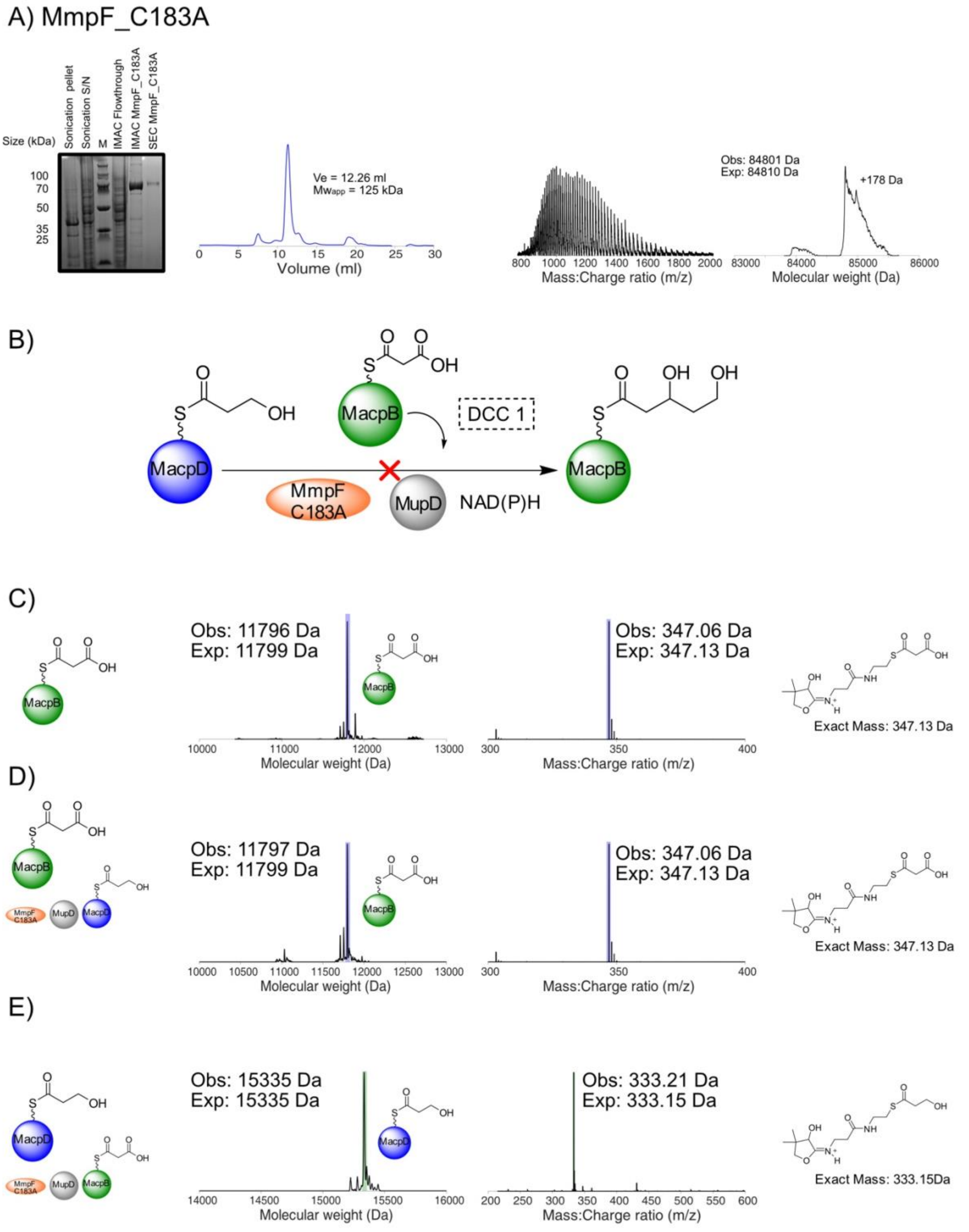

Supplementary Figure 13: Purification of MmpF_C183A and *in vitro* ESI-MS assays. A) Purification of MmpF_C183A. SDS-PAGE following the purification of MmpF_C183A.6His after sonication to isolation via IMAC and SEC. Analytical SEC showing MmpF_C183A.6His eluting as a single oligomeric species. ESI-MS of denatured MmpF_C183A.6His yielding monomeric MmpF_C183A.6His: Expected molecular weight: 84,810 Da. B) Proposed reaction scheme for the decarboxylative claisen condensation of 3HP-MacpD with MmpF_C183A, Malonyl-MacpB, MupD and NAD(P)H to yield 3, 5-hydroxypentanoyl MacpB. C) Deconvoluted spectra and corresponding Ppant ejection of Malonyl-MacpB (green). D) Deconvoluted spectra and corresponding Ppant ejection of MacpB (green) when incubated with 3HP-MacpD and Malonyl-MacpB. E) Deconvoluted spectra and corresponding Ppant ejection for MacpD (blue) when incubated with MmpF_C183A and MacpB_malonyl. For each panel, the observed and expected mass is highlighted.

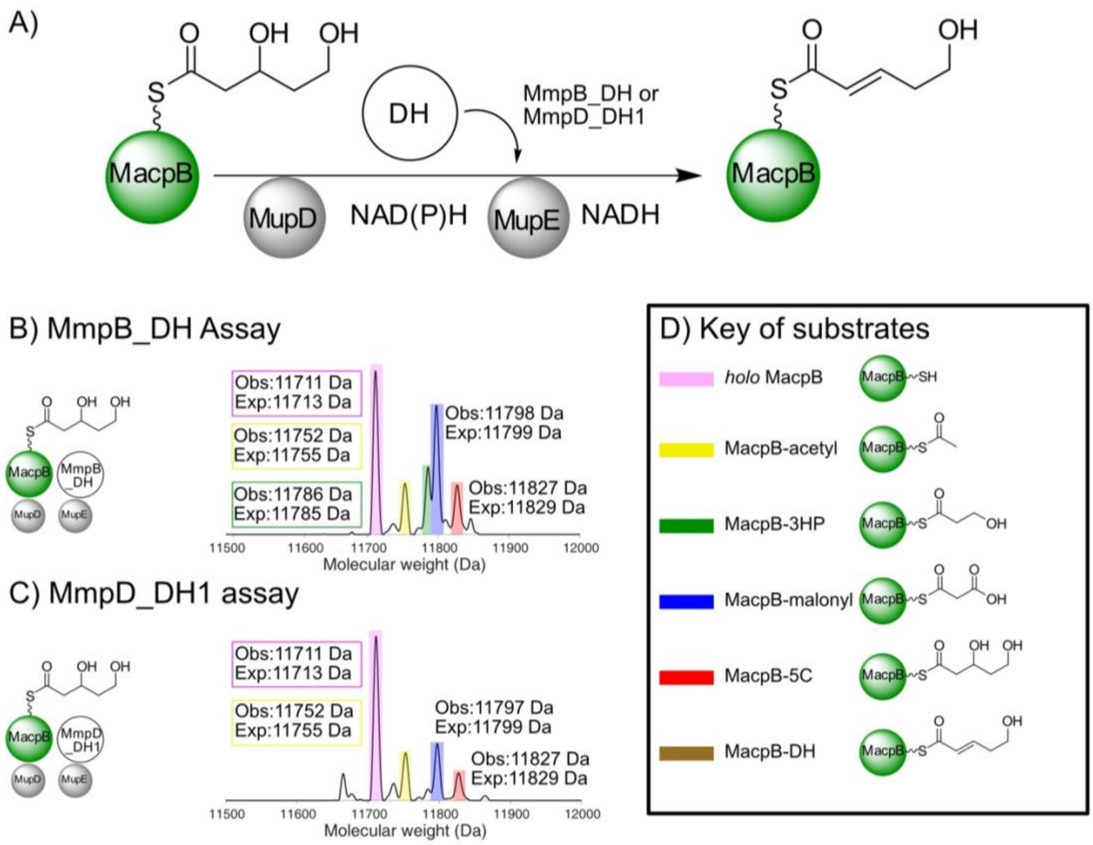

Supplementary Figure 14: Dehydration assays with MmpB_DH and MmpD_DH1 domains. A) Proposed mechanistic scheme for the dehydration of species 3, 5-dihydroxypentanoyl MacpB by either MmpB_DH or MmpD_DH1. The proposed enoyl reductase MupE and NADH are also included to aid the tailoring enzyme reaction. B) Deconvoluted spectra of 3, 5-dihydroxypentanoyl MacpB when incubated with MmpB_DH and C) when incubated with MmpD_DH1. D) Key of observed and expected compounds produced in this assay. No presence of the enoyl species (brown) is observed in either assay.

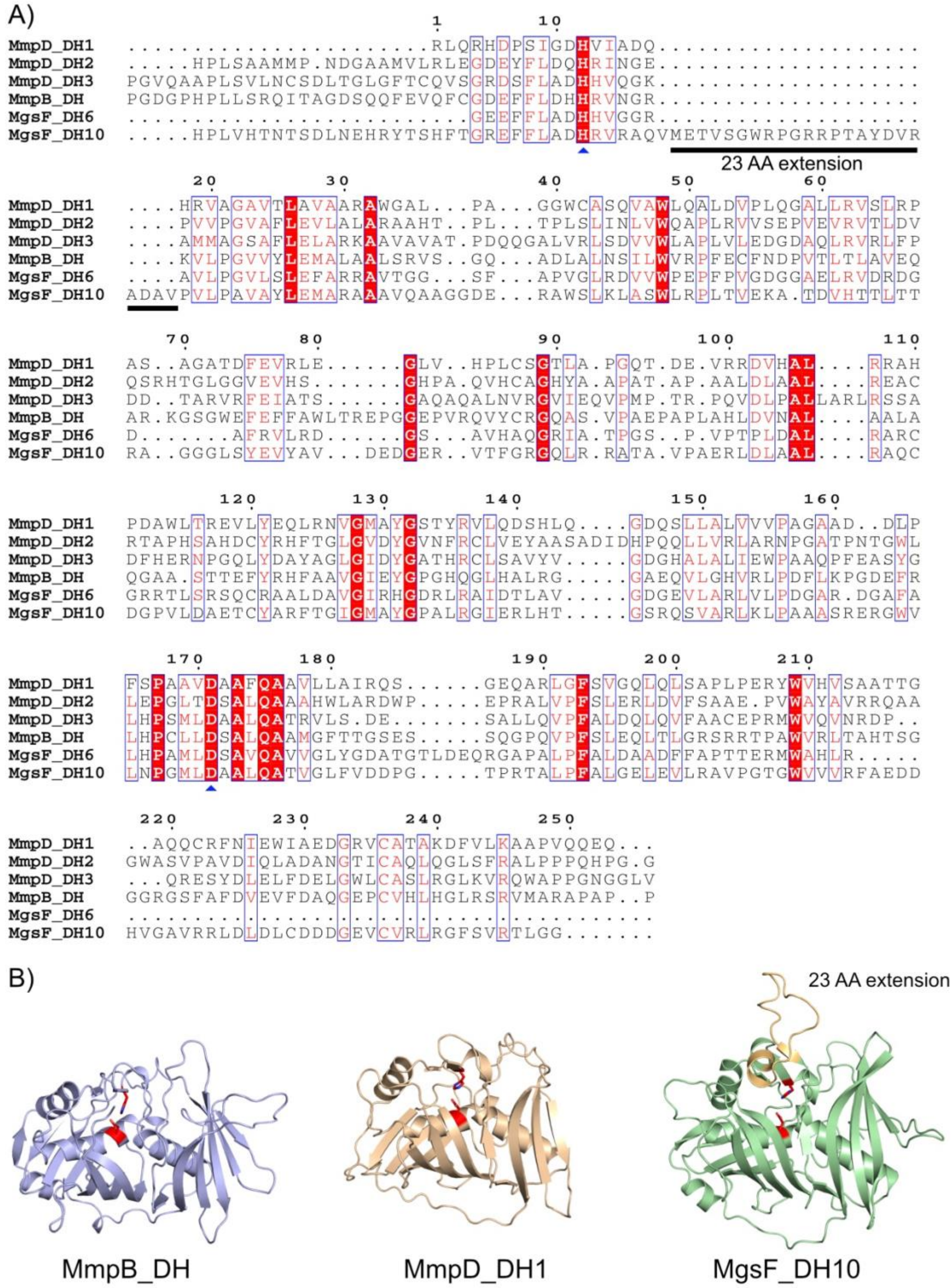

Supplementary Figure 15. Bioinformatic analysis of DH domains within the mupirocin biosynthetic pathway. A) Multiple sequence alignment of mupirocin type 1 encoded DH domains (MmpD_DH1, MmpD_DH2, MmpD_DH3 (Uniprot: Q8RL72) and MmpB_DH (Uniprot: Q8RL74)) with DH domains encoded within *Streptomyces platensis* subsp. rosaceus: MgsF_DH6 and MgsF_DH10 (Uniprot: D0U2E4). Blue arrows represent the conserved catalytic histidine and aspartic acid dyad and the 23 amino acid extension solely encoded within MgsF_DH10 that enables the long-range acting dehydratase activity.[20] B) *Ab initio* homology modelling of MmpB_DH (blue), MmpD_DH1 (beige) (both using Phyre2[21] and MgsF_DH10 (green)[22] where the structure consists of two 'hot-dog' folds, as observed in previously characterised dehydratase domains.[23] For each DH domain the catalytic dyad is highlighted in red. The 23 amino acid extension in MgsF_DH10 is highlighted in orange.

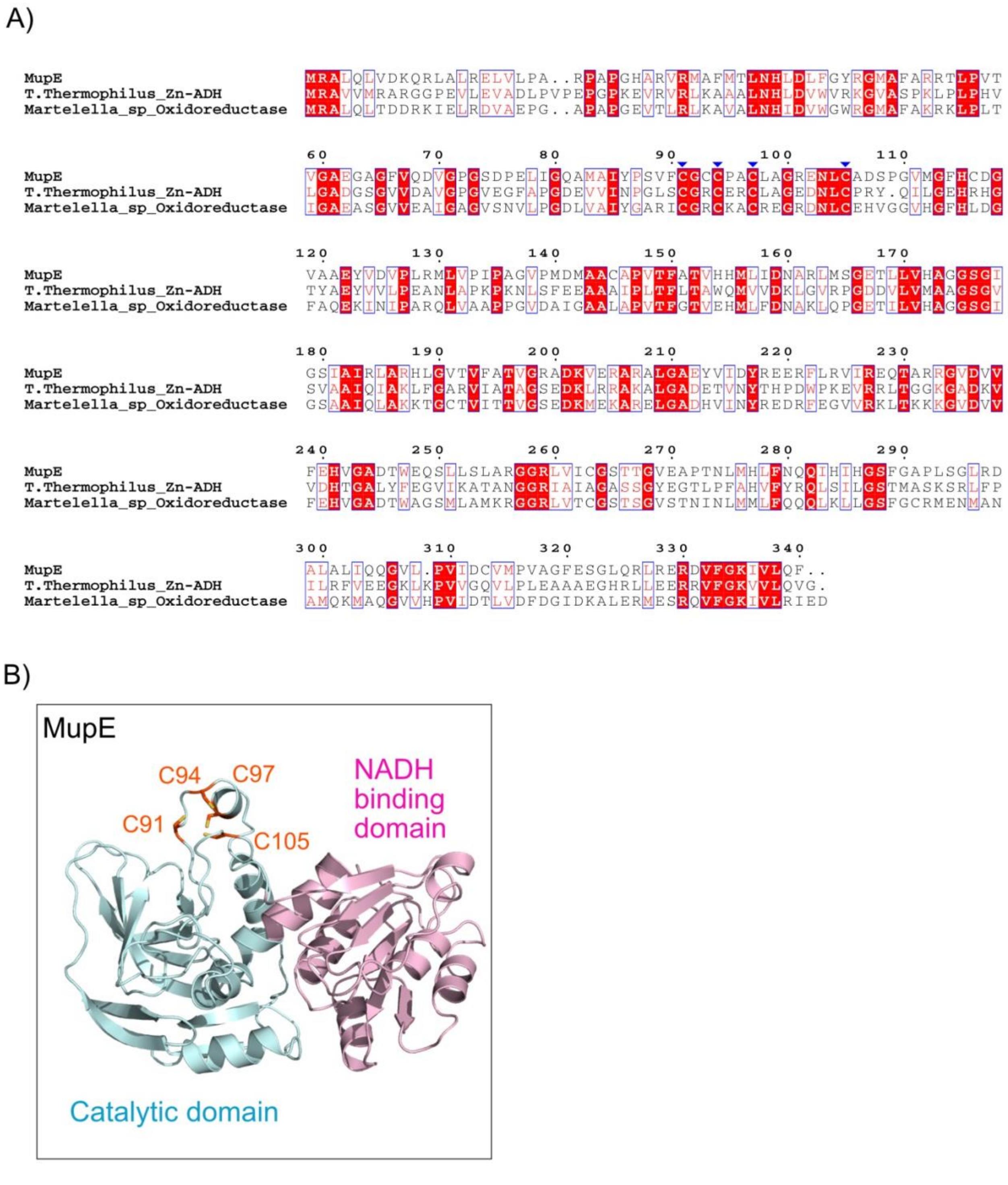

Supplementary Figure 16. Bioinformatic analysis of MupE. A) Multiple sequence alignment of MupE with *Thermus Thermophilus* Zn-ADH (Uniprot: Q5SL27, 37.46% sequence identity) and *Martelella* sp. oxidoreductase (accession number: MAU21815 , 54.12% sequence identity). Amino acids that form a putative zinc binding site are highlighted by blue arrows. B) *Ab initio* homology model of MupE, the two domains: catalytic and NADH binding domain are highlighted in cyan and pink respectively. Within the catalytic domain, there is a cysteine tetrad that forms a putative zinc binding site.

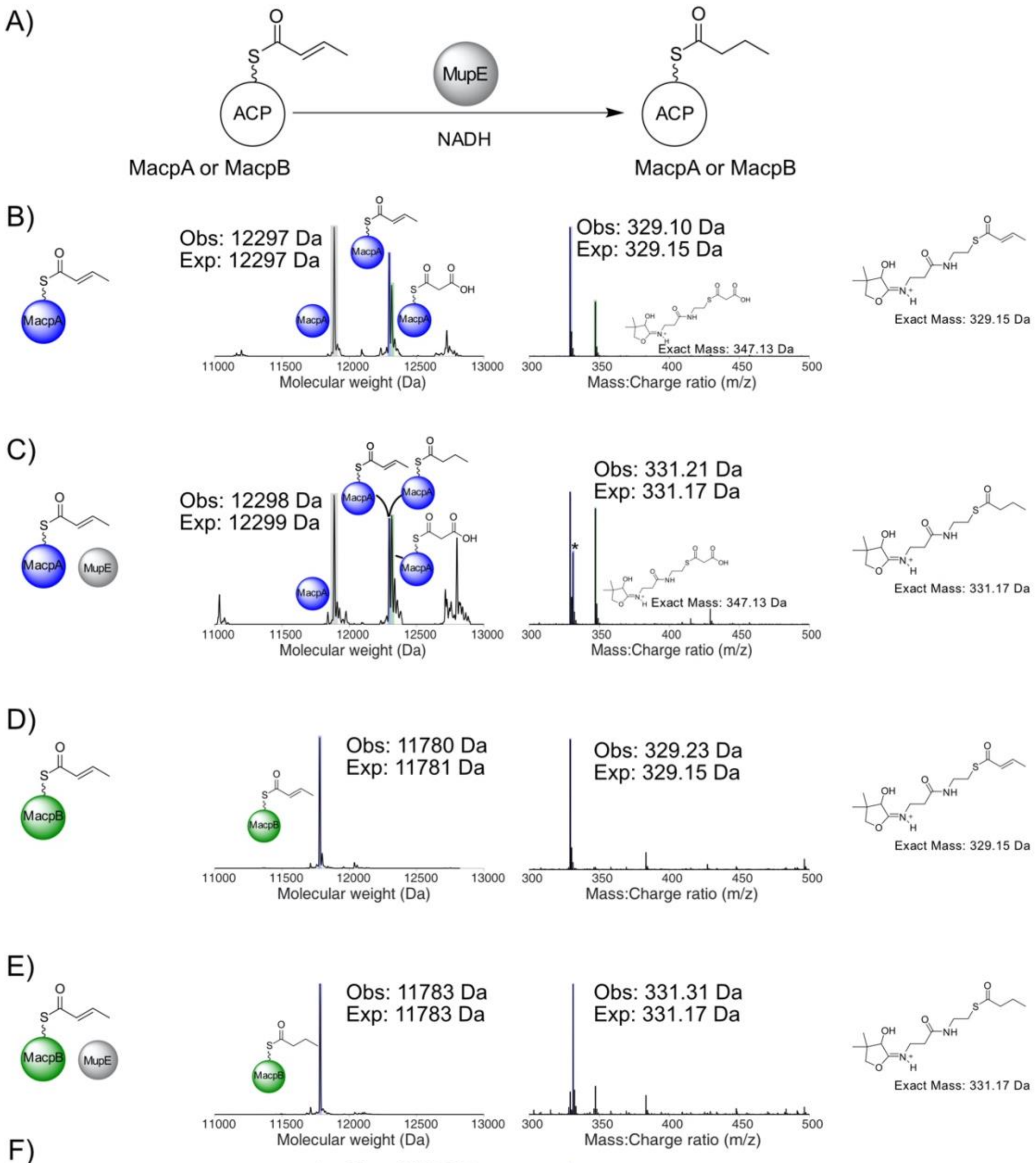

Supplementary Figure 17: Enoyl reductase assays of MupE with crotonoyl-MacpA and crotonoyl-MacpB. A) Proposed reaction scheme of the enoyl reductase activity of MupE either on MacpA or MacpB bearing a control crotonyl pantetheine moiety with the cofactor NADH to yield butyryl-ACP. B) Deconvoluted spectra and corresponding Ppant ejection for upgraded MacpA-crotonyl with observed and expected mass indicated (blue). C) Reaction of crotonyl-MacpA and MupE monitored by ESI-MS. Deconvoluted and corresponding Ppant ejection spectra are highlighted. (*) highlights a new ion corresponding to 331.21 Da: the expected mass for the reduction of crotonyl substrate to butyryl group. A adduct corresponding to malonyl-MacpA was also formed (green). D) Deconvoluted spectra and corresponding Ppant ejection for upgraded crotonyl-MacpB with observed and expected mass indicated (blue). E) Reaction of crotonyl-MacpB and MupE monitored by ESI-MS with reduction to butyryl-MacpB observed. F) Reaction of crotonyl-MacpB and NADH in the absence of MupE, with no reduction to butyryl-MacpB observed.

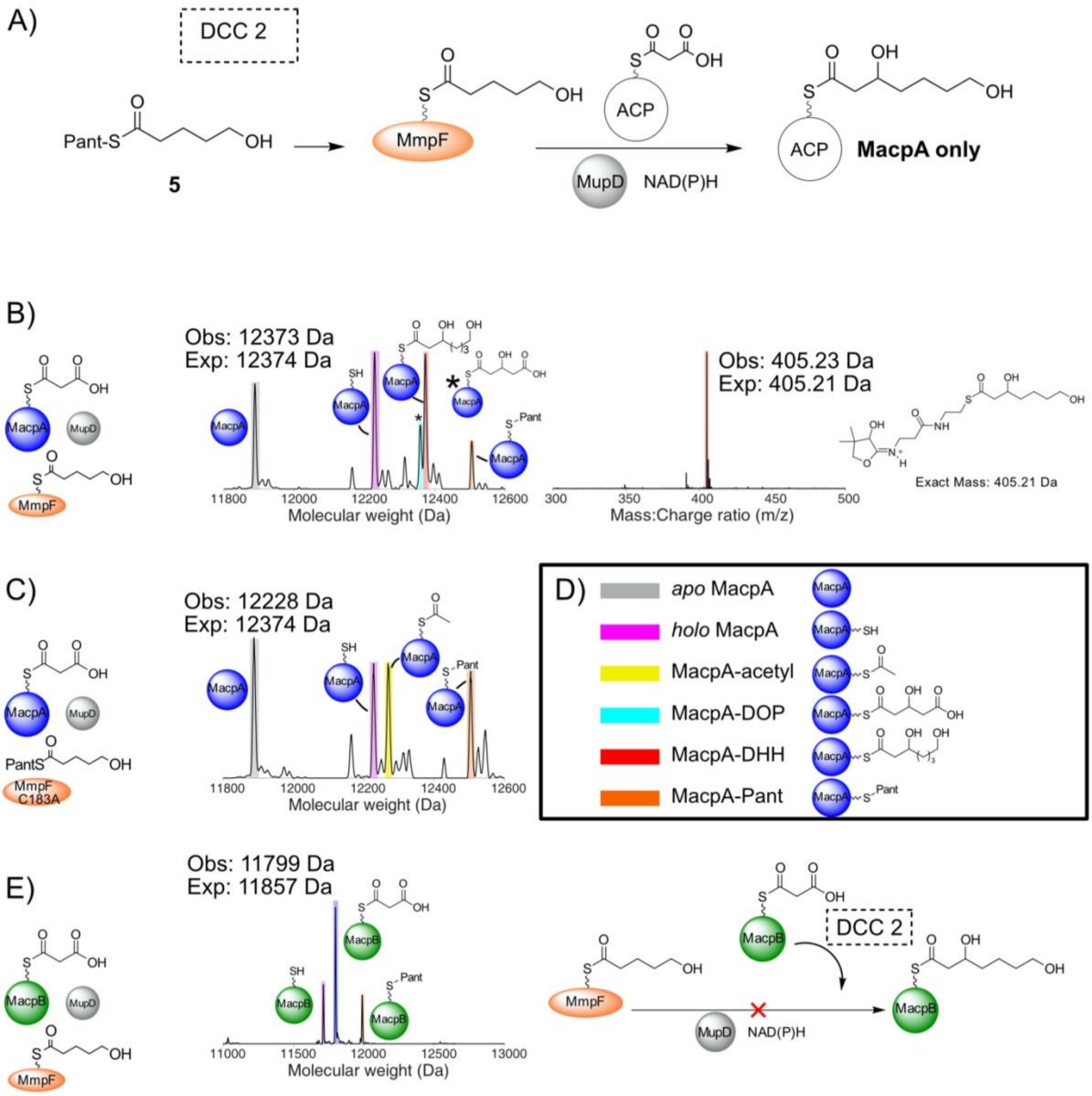

Supplementary Figure 18: MmpF is capable of catalysing the C_5 to C_7 decarboxylative Claisen condensation reaction. A) Proposed reaction scheme of the reaction between 5HP-MmpF and either MacpA or malonyl-MacpB alongside MupD and NAD(P)H to catalyse formation of an ACP bound 7-carbon intermediate bearing a β-hydroxyl group. B) Deconvoluted spectra and Ppant ejection from the malonyl-MacpA, MmpF, 5HP and MupD assay. *apo* MacpA (grey), *holo* MacpA (pink) and *holo* MacpA with pantetheine bound (orange) are formed as adducts. The major peak is the species (red) (observed: 12,373 Da, expected: 12,374 Da) corresponding to a C_7 intermediate with a Ppant ejection of 405.23 Da (expected: 405.21 Da). A side reaction arising presumably from transfer of malonate to MmpF and DCC with malonyl-MacpA followed by ketoreduction led to generation of a significant proportion of MacpA bound 3-hydroxy, pentan-1, 5-dioic acid (cyan). C) Deconvoluted spectra when malonyl-MacpA is used as the acceptor ACP with MmpF_C183A mutant, 5HP and MupD. *apo* MacpA (grey), *holo* MacpA (pink) and *holo* MacpA with pantetheine bound via transthiolation (orange) are formed as adducts. A side product of acetyl-MacpA (yellow) was also observed. D) Key of MacpA species produced during ESI-MS assays. E) Deconvoluted spectra of MacpB when malonyl-MacpB is used as the acceptor ACP with MmpF, 5HP and MupD. No reaction was observed with *apo* MacpB (pink), malonyl MacpB (blue) and *holo* MacpB with pantetheine bound (orange) observed.

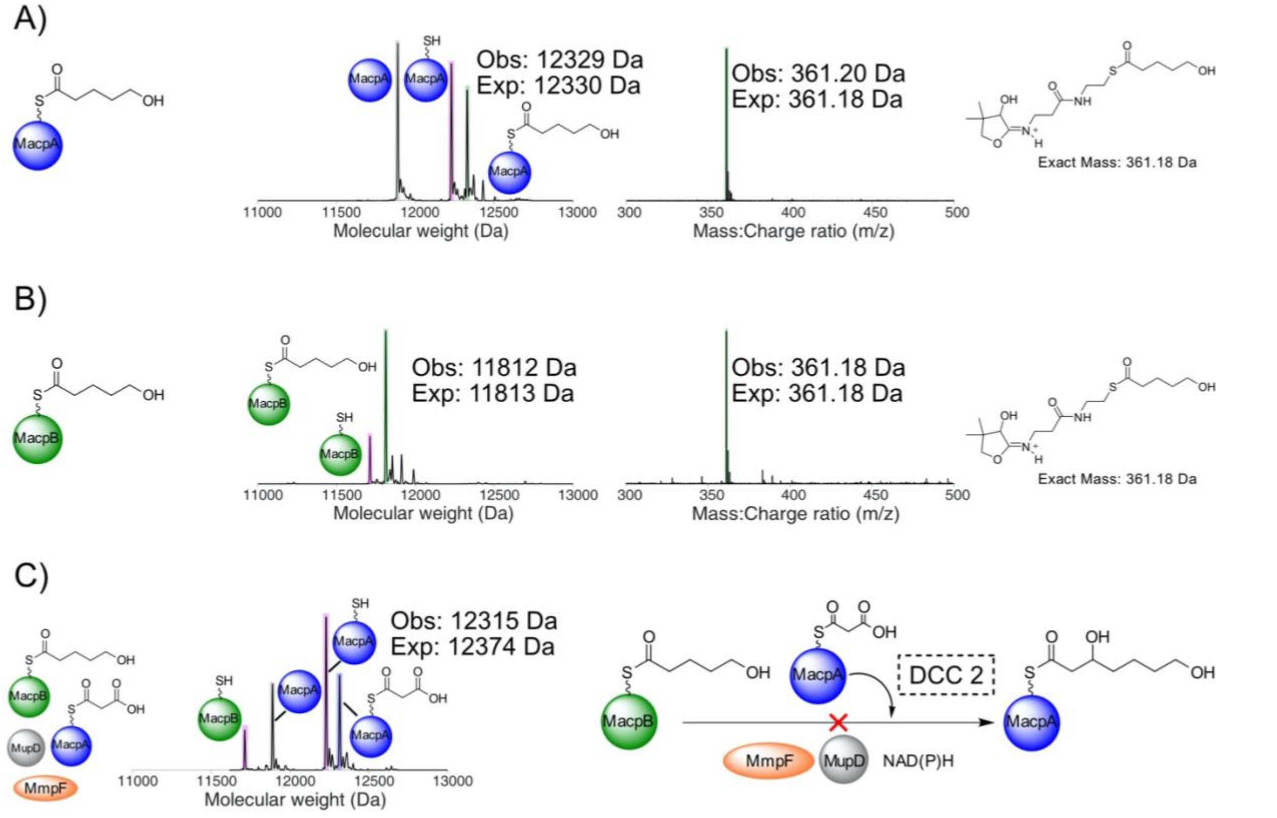

Supplementary Figure 19: Generation of 5HP-ACP substrates monitored by ESI-MS and Ppant ejection assays. A) Deconvoluted spectra and Ppant ejection from 5HP-MacpA upgrade reaction. The following species were observed; *apo* MacpA (grey), *holo* MacpA (pink) and 5HP-MacpA (green). B) Deconvoluted spectra and Ppant ejection of 5HP-MacpB upgrade assay with *holo* MacpB (pink) and 5HP-MacpB (green) observed. C) Deconvoluted spectra when 5HP-MacpB is used as the donor ACP with MmpF, malonyl-MacpA and MupD. No presence of 3,7-dihydroxylheptanoyl-MacpA (red) (exp: 12,374 Da) was observed.

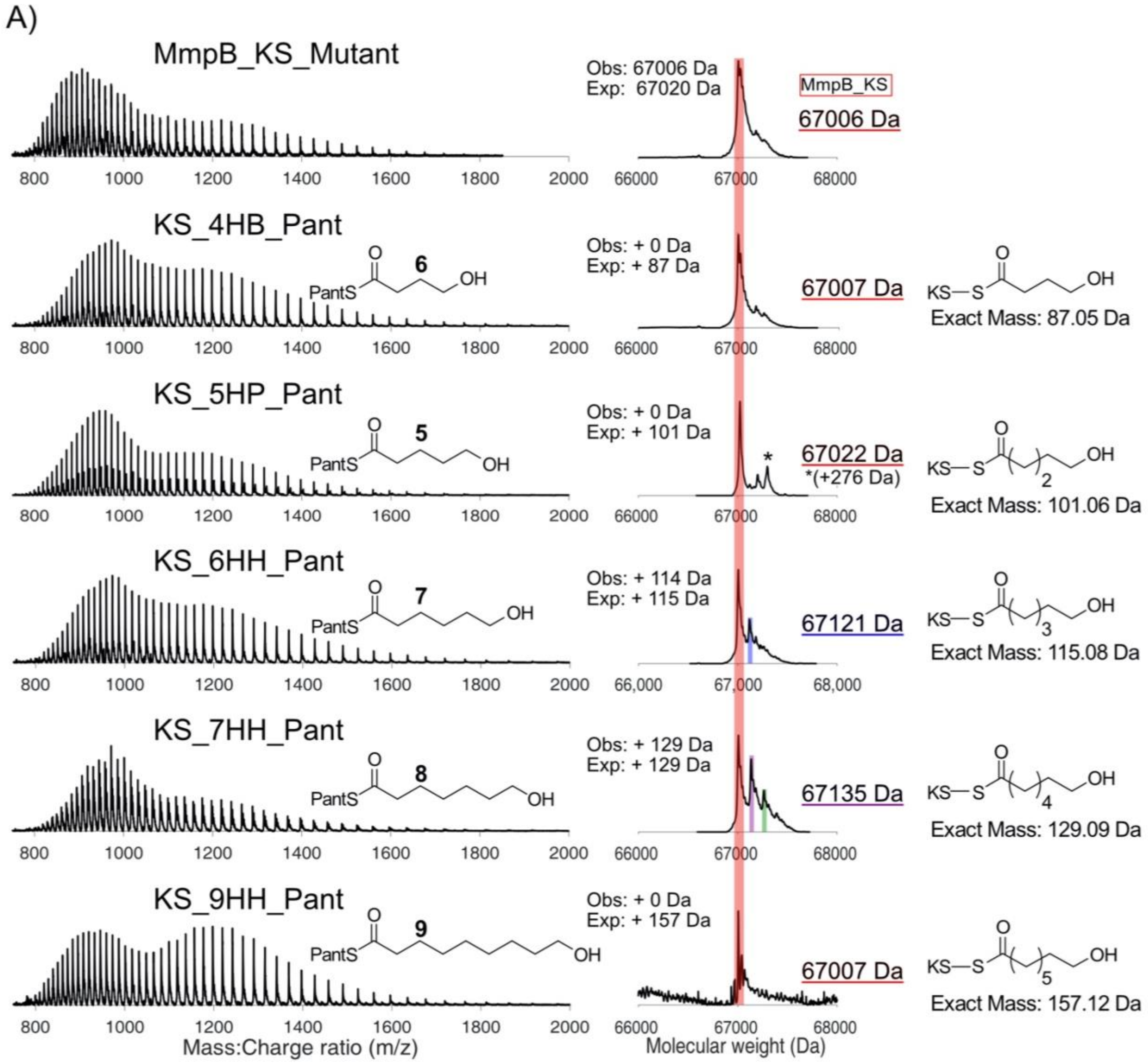

Supplementary Figure 20: Fatty acid binding profile of MmpB KS. A) 40 μM MmpB KS was incubated with 1 mM pantetheine substrate (4HB (**4**), 5HP (**5**), 6HH (**6**), 7HH (**7**) and 9HN (**9**)) to detect binding. Expected molecular weight: MmpB-KS: 67,020 Da, 4HB-MmpB-KS: 67,107 Da (+87 Da), 5HP-MmpB-KS: 67,121 Da (+101 Da), 6HH-MmpB-KS: 67,135 Da (+115 Da), 7HH-MmpB-KS: 67149 Da (+129 Da), 9HN-MmpB-KS: 67,177 Da (+157 Da). *apo* MmpB-KS is highlighted in red, with binding of 6HH (**6**) and 7HH (**7**) highlighted in blue and purple respectively with their observed molecular weight indicated. Highlighted in green is a +178 adduct associated with phosphogluconylation. * refers to the addition of +276 Da, attributed to excess free pantetheine within the 5HP-Pant substrate which non-specifically bound MmpB-KS.

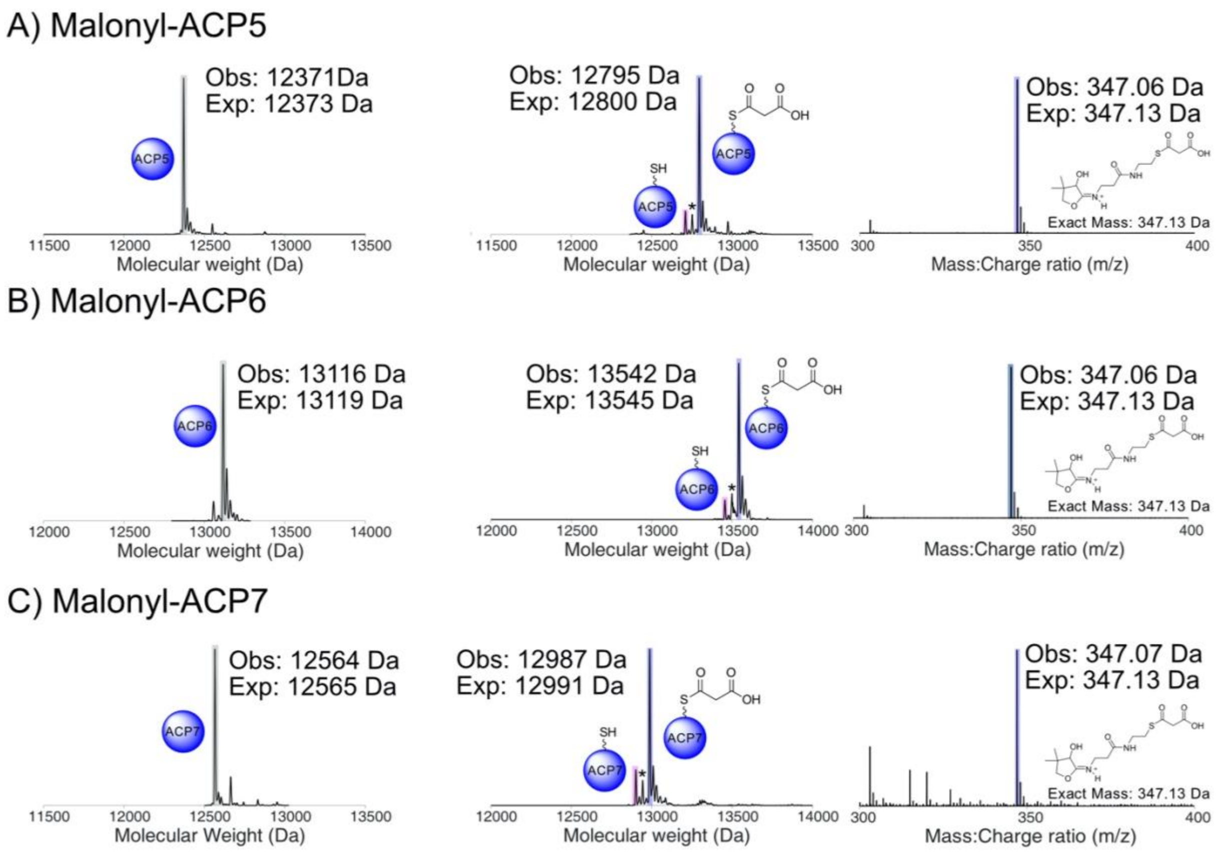

Supplementary Figure 21: Generation of malonyl MmpB_ACPs. Deconvoluted ESI-MS spectra and Ppant ejection assays for A) of *apo* ACP5 (left) and malonyl-ACP5 (right) B) *apo* ACP6 (left) and malonyl-ACP6 (right) C) Deconvoluted spectra of *apo* ACP7 (left) and malonyl-ACP7 (right). For each spectra the observed and expected mass is shown. For malonyl upgrades, adducts corresponding to *holo* ACP (pink) and acetyl-ACP (*) are highlighted.

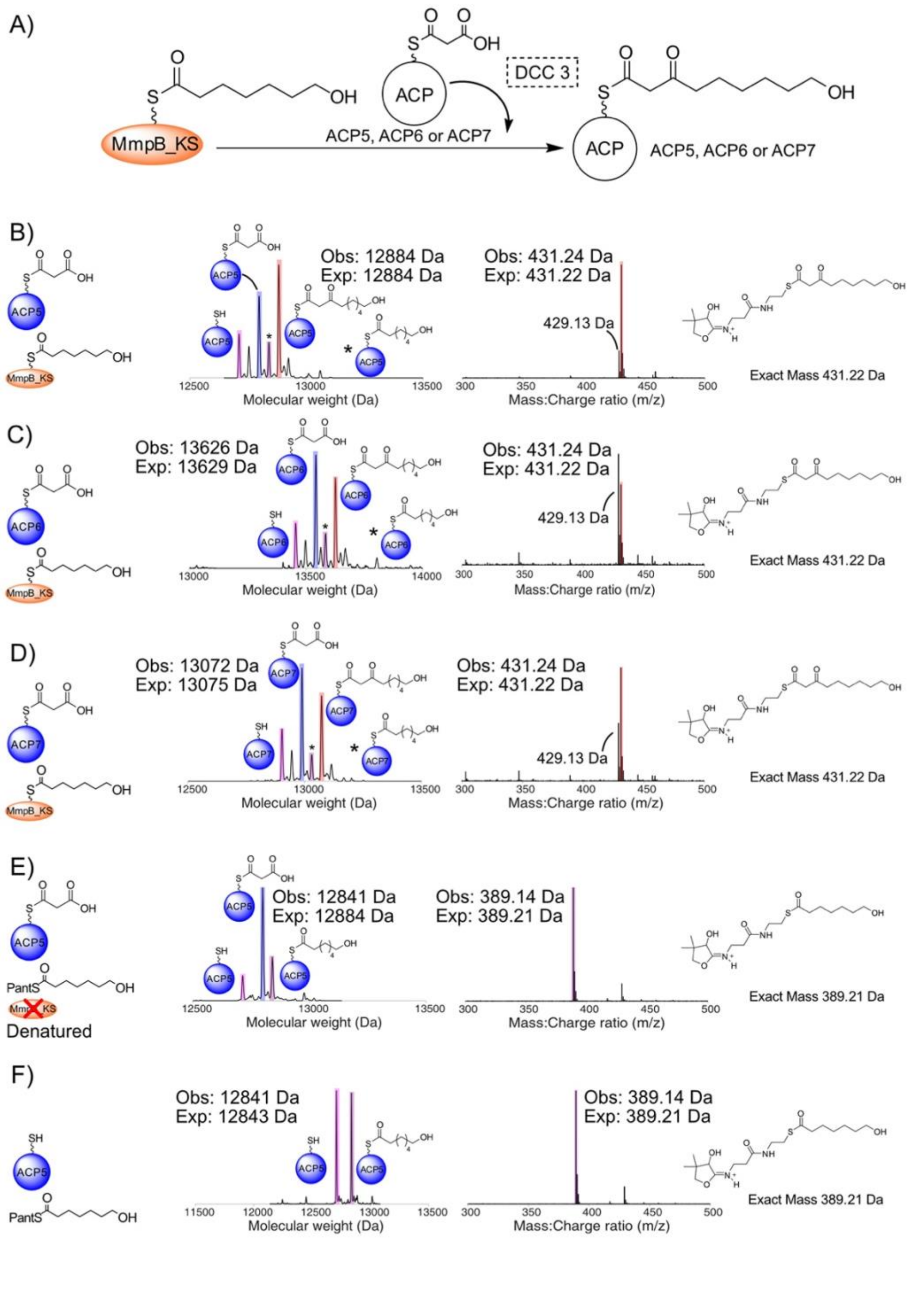

Supplementary Figure 22. Decarboxylative Claisen condensation catalysed by MmpB_KS. A) Proposed reaction scheme for DCC3 from 7HH-pantetheine. B) Deconvoluted spectra and Ppant ejection of malonyl-ACP5 incubated with MmpB_KS and 7HH-pantetheine (left). C) Deconvoluted spectra when Malonyl-ACP5 is incubated with denatured MmpB_KS and 7HH pantetheine. Ppant ejection of the 7HH-ACP5 is shown. D) and E) As C) for ACP6 and ACP7 respectively. F) Deconvoluted spectra when *holo* ACP5 is incubated with 7HH pantetheine. Ppant spectra of 7HH-ACP5 is highlighted. *Holo*-ACP5 was able to self-load 7HH-pantetheine and explains background levels of 7HH-ACP5 arising from the initial generation of *holo* ACP in the assays. Shading as follows: *apo* ACP (pink), malonyl-ACP (blue), 7HH-ACP (purple) and 9-hydroxy-3-oxononanoyl-ACP (red). For Ppant spectra where the 9-hydroxy-3-oxononanoyl-ACP was formed, an additional species at 429.13 Da was observed and is predicted to correlate to oxidation of the terminal hydroxyl group to a carbonyl moiety (exp mass: 429.21 Da)

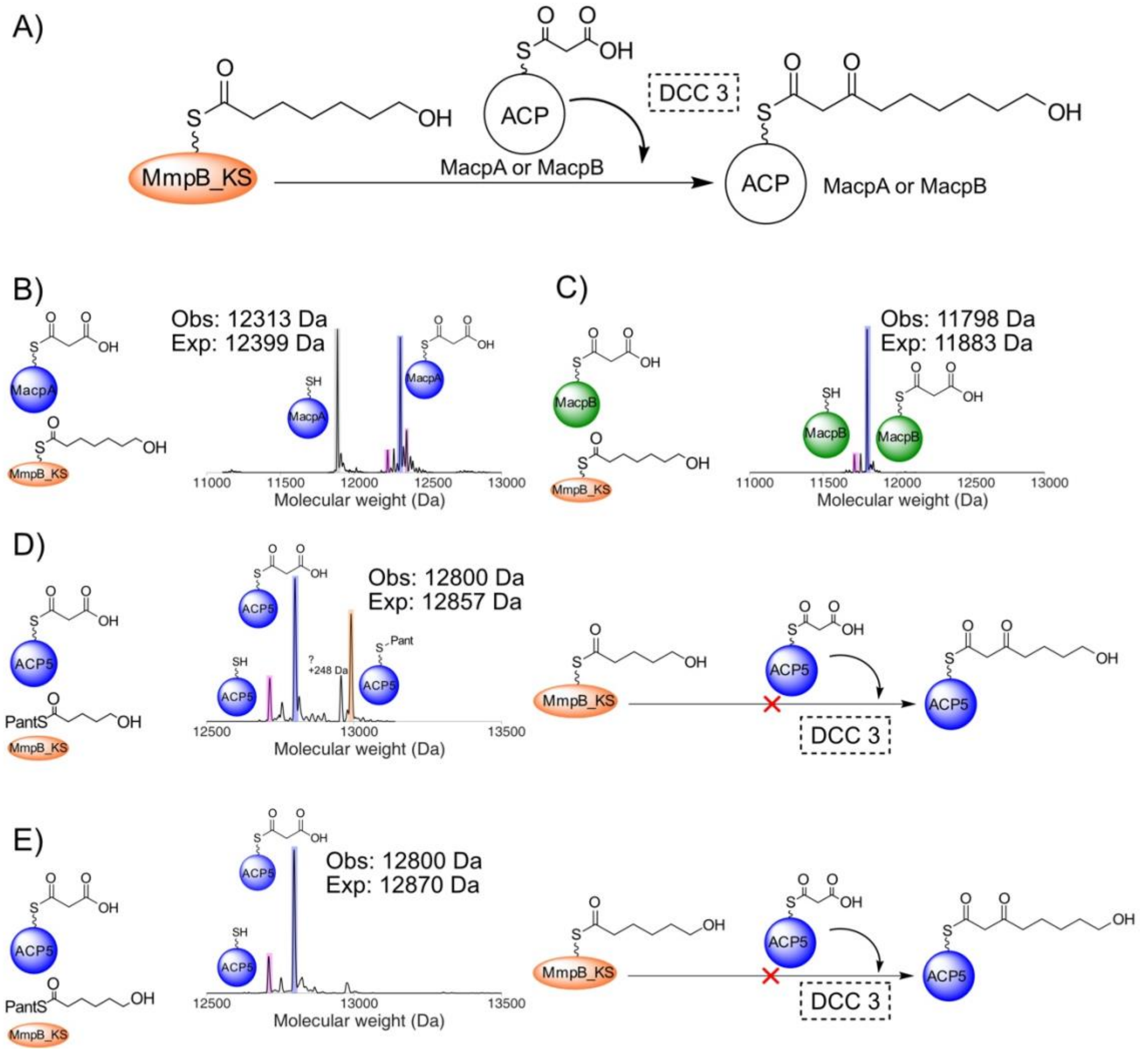

Supplementary Figure 23. Control DCC experiments with MmpB_KS. A) Proposed reaction scheme for DCC 3 where MmpB_KS converts 7HH-pantetheine to a 9-hydroxy-3-oxononanoyl species attached to MacpA or MacpB. B) Deconvoluted spectra when malonyl-MacpA is used as the acceptor ACP within the DCC 3 reaction. C) Deconvoluted spectra when malonyl-MacpB is used as the acceptor ACP within the DCC 3 reaction. D) Deconvoluted spectrum of ACP5 when MmpB_KS is incubated with 5HP pantetheine and malonyl-ACP5. E) Deconvoluted spectra of ACP5 when MmpB_KS is incubated with 6HH pantetheine and malonyl-ACP5. For each spectra, the observed and expected mass are highlighted with species highlighted: *apo* MacpA (grey), *holo* ACP (pink), malonyl-ACP species (blue) and ACP-pantetheine (orange).

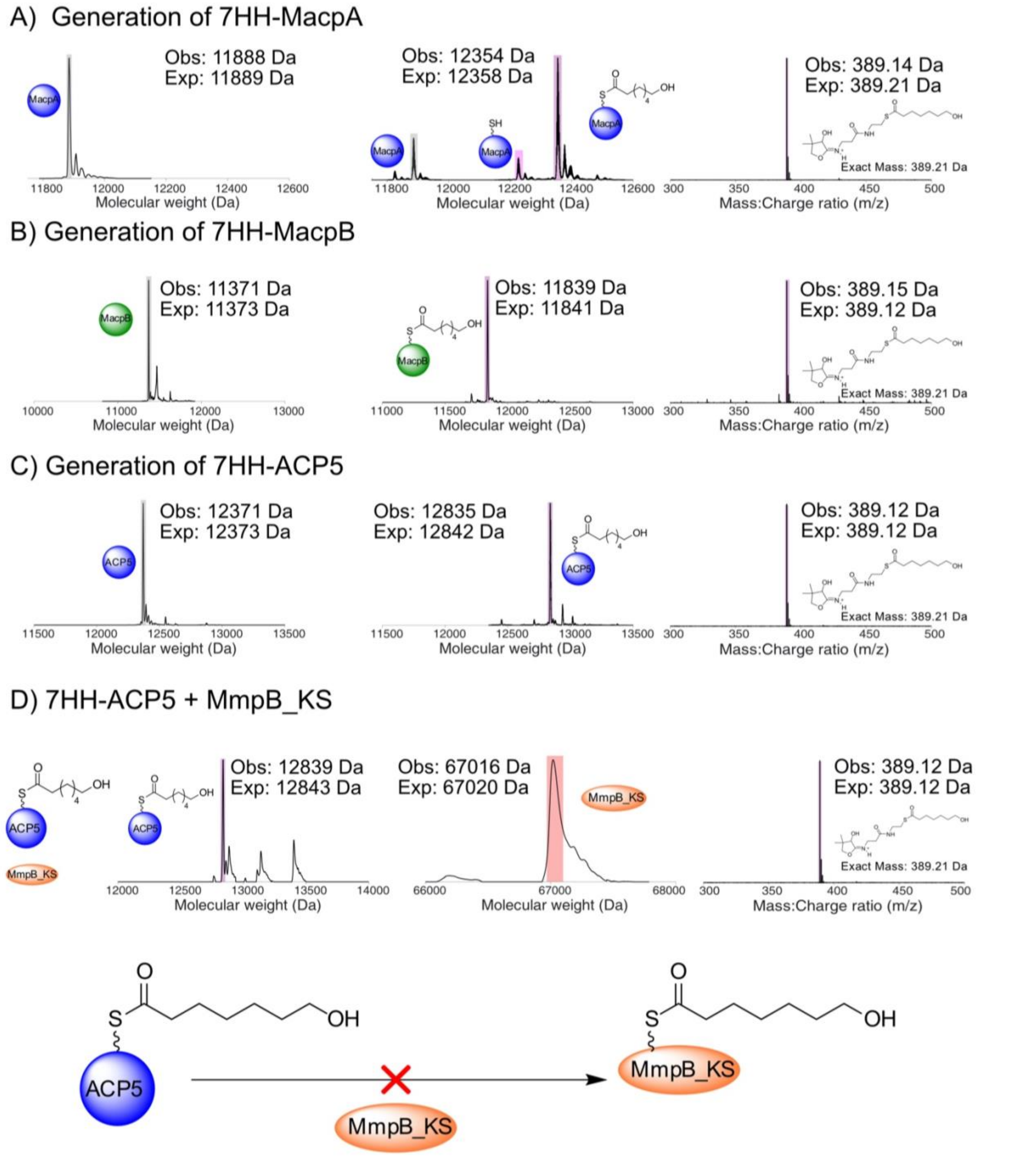

Supplementary Figure 24: Generation of 7HH-ACP substrates. A) Deconvoluted spectra of *apo* MacpA (left), 7HH-MacpA (right) and the Ppant ejection for this species. B) Deconvoluted spectra of *apo* MacpB (left), 7HH-MacpB (right) and the Ppant ejection for this species. C) Deconvoluted spectra of *apo* ACP5 (left), 7HH-ACP5 (right) and the Ppant ejection for this species. D) Transthiolation assay to determine if ACP5 can transfer 7HH-pantetheine to MmpB-KS. Deconvoluted spectrum of ACP5 (left), MmpB-KS (right) and the Ppant ejection for this assay are highlighted. Shading; *apo* MacpA (grey), *holo* MacpA (pink) and 7HH-ACP species (purple). MmpB_KS is highlighted in red.

9. Amino Acid Residue Sequences.

MacpA.6His

MHHHHHHGKPIPNPLLGLDSTENLYFQGIDPFTMNPERRNMYMEEIYTFVVSTLASSCKVQPGDIEPTTNL
FADLGIDSVDFLDAVFCIEKHYDIRIPVGQWMSAVNEGNAAMTDYFVMEHFVAQIAARAAASA

Calculated Mw: 15042.03 Da

MacpA (His$_6$ cleaved)

GIDPFTMNPERRNMYMEEIYTFVVSTLASSCKVQPGDIEPTTNLFADLGIDSVDFLDAVFCIEKHYDIRIP
VGQWMSAVNEGNAAMTDYFVMEHFVAQIAARAAASA

Calculated Mw: 11889.48 Da

MacpB

AHHHHHHSSGLEVLFQGPMEINVAQQTVSESIRELKLPGWETASIETETHLQGELGIDSLHKLILLTRLQE
RSNFQFAQLNNEAYKFDTVGDLVNLLVAHG

Calculated Mw (-M) = 11372.74 Da

MacpD

MHHHHHHGKPIPNPLLGLDSTENLYFQGIDPFTLNHQVMDQVFDQVEHQIAQVLGAKGGPLVAVEIDSRFS
DLGLSSLDLATLISNLEAVYGTDPFADAVAITSIVTVADLARAYAQQGVPGPSPDPLDAQLRDLRQL

Calculated Mw: 14922.96 Da

MmpB_ACP5

MHHHHHHGKPIPNPLLGLDSTENLYFQGIDPFTRSQVDPSDTLRGLVGQILKVDAQEIDDTTAFSDMGFDS
VMLTELATAINRTYTLELGTAALFEHPTLQALAAHLQGARTA

Calculated Mw: 12373.88 Da

MmpB_ACP6

MHHHHHHGKPIPNPLLGLDSTENLYFQGIDPFTPPAPGLTRAQVAQGVREVVAEALKVRLEDIGDDDPWSD
YGMDSVSSVQMTGLLNERFDIQLAADTFQAFGNVVELTTAIADIQVVMA

Calculated Mw: 13118.74 Da

MmpB_ACP7

MHHHHHHGKPIPNPLLGLDSTENLYFQGIDPFTIADTALLDELVALVCQLLKTVAGDIDPHTDLHDFGFDS
VLLTQLLAQISSTYGVELDPGSVLEDATVAGLVAQVQAQRHGAEPA

Calculated Mw: 12565.13 Da

MmpB_DH

MHHHHHHGKPIPNPLLGLDSTENLYFQGIDPFTPGDGPHPLLSRQITAGDSQQFEVQFCGDEFFLDHHRVN
GRKVLPGVVYLEMALAALSRVSGQADLALNSILWVRPFECFNDPVTLTLAVEQARKGSGWEFEFFAWLTRE
PGGEPVRQVYCRGQASVPAEPAPLAHLDVNALAALAQGAASTTEFYRHFAAVGIEYGPGHQGLHALRGGAE
QVLGHVRLPDFLKPGDEFRLHPCLLDSALQAAMGFTTGSESSQGPQVPFSLEQLTLGRSRRTPAWVRLTAH
TSGGGRGSFAFDVEVFDAQGEPCVHLHGLRSRVMARAPAPP

Calculated Mw: 35364.93 Da

MmpB_KS_CDD

MHHHHHHGKPIPNPLLGLDSTENLYFQGIDPFTDIAIIGLAGRYPQAENIEELWENLKLGRDCITTVPSQR
WDHDAIYDPSKGVSGKTYSKWGGFLRGVDEFDPRFFNISPREAEIMDPQERLFLQCAYHVLEDAGYTRQSL
SAKGRVGVYVGVQYTEYTAFSTAQTLVAALPASIANRVSFFCDFRGPSLTLDSMCSSSLTTIHLACQSLRS
GESEYAIAGGVNVSIHPNKYLLHAQGRFASSVGCCKTFGQGGDGYVPAEGVGAVLLKPLPQAIADGDRIHA
VIKGSSINHGGRATGFTVPKSSAQAVAVRSALHQAGLASSDLSYIEAHGTGTALGDLIEIEGLRTVFEADG
FEPQTCAIGSIKSNIGHCESAAGIAGLTKVLLQLKHRQLVPSLHSTQLNRNIDFAGSPFHVQQTLEPWLPK
GAADAVQPRRAGISSFGAGGSNAHLVVEEYSLQAQAHTHVPTLPSDVLIVLSARTHERLQAVAALLLGRLE
QTPLACDLAGLHDLAYTLQVGREALDARAAFTAQSVQVLKERLVALADGAQHPDVLIGQALKPVRLRAGET
AVPVQSPAMDDNQAVMRHWVSGGQVEWAQLYQGFQPARISLPLYPFVRERAWVP

Calculated Mw: 67020.91 Da

MmpD_DH

MAHHHHHHSSGLEVLFQGPRLQRHDPSIGDHVIADQHRVAGAVTLAVAARAWGALPAGGWCASQVAWLQAL
DVPLQGALLRVSLRPASAGATDFEVRLEGLVHPLCSGTLAPGQTDEVRRDVHALRRAHPDAWLTREVLYEQ
LRNVGMAYGSTYRVLQDSHLQGDQSLLALVVVPAGAADDLPFSPAAVDAAFQAAVLLAIRQSGEQARLGFS
VGQLQLSAPLPERYWVHVSAATTGAQQCRFNIEWIAEDGRVCATAKDFVLKAAPVQQEQ

Calculated Mw: 29297.19 Da

MmpF

MGSSHHHHHHSSGLVPRGSHMASMTGGQQMGRGSEFVSQALESHLGDLAVVGLACRFPGAPDQRVFWENLR
QGRESIVDLDDQQLSAAGVTREQWSEPGYIRRSPVLEGIEWFDARFFGVSAREAILLDPQHRLLLEVGYEA
LEDAGHVKAAQAGVTGVYACMGGITTSYLHQFGDRLDPLHHETASLVHQGNDKDFLATRLSFKLNLTGPSM
TVQTACSSSLVALHLACNALRLGEVDTALVGAAAIRIPHHTGYPLGQSPLLSRDGRCCPFSSDASGTLFGS
GVASVIVRRHADAIRDGDHVYALIKSSHVNNDGAMKIGYTATSVPGQAKAMVRAITLARANARQISYVECH
GTATSLGDPLEIKALEKAFRLDTQDHGFCAIGSVKSNIGHLEQAAGIASLIKVALMLKHDTLVPSLNFTTP

NPRIDFEHSPFRVSQDTRVWSQALEQPADTARLAAINCLGIGGTNVFSVLQSVAPALVRGTDAAVDVPICL
SAATREQLRQYLLRFAHFVRDSGPIDRLALAHTINISRSAHRERFAGVLKAGIDVDRFFEEAAHSVLDAPA
TFTPRLVYYLCEQPQHLDDASRQAWLAAPRYQRMADDYAECLGRLSSTPQTVEAAARCAELAFEVALYQQL
VRWGMVFEAVIDRGYGHWINRCLQLGCGASSALDASPEWLSAFVQEGQGAAQLASPGWPDSAQVAVLVVSR
QAWAVTVPSSGRCVHLAEEMLTFADVERFACDVVQAGISFDWLDYYRLVKAQKLSLPTYPFARQRYWPVED

Calculated Mw (-M): 84842.02 Da

MmpF_C183A

MGSSHHHHHHSSGLVPRGSHMASMTGGQQMGRGSEFVSQALESHLGDLAVVGLACRFPGAPDQRVFWENLR
QGRESIVDLDDQQLSAAGVTREQWSEPGYIRRSPVLEGIEWFDARFFGVSAREAILLDPQHRLLLEVGYEA
LEDAGHVKAAQAGVTGVYACMGGITTSYLHQFGDRLDPLHHETASLVHQGNDKDFLATRLSFKLNLTGPSM
TVQTAASSSLVALHLACNALRLGEVDTALVGAAAIRIPHHTGYPLGQSPLLSRDGRCCPFSSDASGTLFGS
GVASVIVRRHADAIRDGDHVYALIKSSHVNNDGAMKIGYTATSVPGQAKAMVRAITLARANARQISYVECH
GTATSLGDPLEIKALEKAFRLDTQDHGFCAIGSVKSNIGHLEQAAGIASLIKVALMLKHDTLVPSLNFTTP
NPRIDFEHSPFRVSQDTRVWSQALEQPADTARLAAINCLGIGGTNVFSVLQSVAPALVRGTDAAVDVPICL
SAATREQLRQYLLRFAHFVRDSGPIDRLALAHTINISRSAHRERFAGVLKAGIDVDRFFEEAAHSVLDAPA
TFTPRLVYYLCEQPQHLDDASRQAWLAAPRYQRMADDYAECLGRLSSTPQTVEAAARCAELAFEVALYQQL
VRWGMVFEAVIDRGYGHWINRCLQLGCGASSALDASPEWLSAFVQEGQGAAQLASPGWPDSAQVAVLVVSR
QAWAVTVPSSGRCVHLAEEMLTFADVERFACDVVQAGISFDWLDYYRLVKAQKLSLPTYPFARQRYWPVED

Calculated Mw (-M): 84809.96 Da

MupB

MAHHHHHHSSGLEVLFQGPMRTAYLASTAYVLGEQAHDYRDAASFEAVCKQHSMPDFASVFGWGTYWRTTR
SVGELLVESVASTLASSGLRGCDIDALVVCSSNFESGQVVDYLPLLRELQLARAFPLGVTWGDCTMLLAGL
EVARAQVLAGLDNVLVVSANRIEDEAFRFQHYALFSDGAASCLVTSGRRRGFEMLGSLARSNAGLAHDPKE
DDTRLFREVHEQFMHRQQINTADLEQVVCSNVFLPVLKIKEGRQGVSGTQLYVDNVTRVGHCFSADSLINL
CDYQARAQQVHGGLVMLTANADGLRCQTLLQRVSDA

Calculated Mw (-M): 35000.52 Da

MupD

MAHHHHHHSSGLEVLFQGPMRRQVVVTGGGRGIGASISTALVQAGFVVLIVGHRGISAGVALCEELDARYG
AGSARWVGCDLGDALAVEGLCRSLEAEDIYGLVHCAGVSQDSLAAVADLDTARHMMQVNFWAFVQLYQALV
RPMSMRRAGRILCIGSVAADFGMKGNALYASSKAALRGFVRSSMTEVASRAITINCIEPGFIETALLDRYA
EQRSAIDRRIPAGRYGSPEEVAGLAAFLFSEAGSYINGQHLTVDGGLSRSMAG

Calculated Mw (-M): 28032.99 Da

MupE

MAHHHHHHSSGLEVLFQGPMRALQLVDKQRLALRELVLPARPAPGHARVRMAFMTLNHLDLFGYRGMAFAR
RTLPVTVGAEGAGFVQDVGPGSDPELIGQAMAIYPSVFCGCCPACLAGRENLCADSPGVMGFHCDGVAAEY
VDVPLRMLVPIPAGVPMDMAACAPVTFATVHHMLIDNARLMSGETLLVHAGGSGIGSIAIRLARHLGVTVF
ATVGRADKVERARALGAEYVIDYREERFLRVIREQTARRGVDVVFEHVGADTWEQSLLSLARGGRLVICGS
TTGVEAPTNLMHLFNQQIHIHGSFGAPLSGLRDALALIQQGVLPVIDCVMPVAGFESGLQRLRERDVFGKI
VLQF

Calculated Mw (-M): 38595.83 Da

TacpB

MHHHHHHGKPIPNPLLGLDSTENLYFQGIDPFTMPHINYISIHGERDNMSNEQASTAKALVISFIDQMNRS
EYRGSDIRESFELKNMLNIDSLNKLILLTRITEHLGVEFGESDITINDFTTVSDLISFVETHTRMAETHTE
PS

Calculated Mw: 16457.41 Da

Table S1: A list of primers used in this study.

Primers	Sequence (5’ -3’)
MmpF_FOR_pET28a	TGGGTCGCGGATCCGAATTCGTGAGCCAGGCCCTAGAG
MmpF_REV_pET28a	CAAGCTTGTCGACGGAGCTCTCAGTCTTCAACTGGCCAAT
MupD_FOR_pOPINF	AAGTTCTGTTTCAGGGCCCGATGCGTAGGCAGGTAGTCGT
MupD_REV_pOPINF	ATGGTCTAGAAAGCTTTACTAGCCGGCCATGCTG
MupE_FOR_pOPINF	AAGTTCTGTTTCAGGGCCCGATGCGGGCGCTTCAA
MupE_REV_pOPINF	ATGGTCTAGAAAGCTTTATTAGAATTGCAATACGATCCTGCC
MacpB_FOR_pOPINF	AAGTTCTGTTTCAGGGCCCGATGGAAATCAACGTGGCG
MacpB_REV_pOPINF	ATGGTCTAGAAAGCTTTACTAGCCATGCGCTACCAG
MmpF_C183A_FOR	GTGCAGACCGCGGCGTCAAGTTCGCTG
MmpF_C183A_REV	CAGCGAACTTGACGCCGCGGTCTGCAC
MmpF_C183A_FOR_2	ACCGTGCAGACCGCGGCGTCAAGTTCGCTG
MmpF_C183A_REV_2	CAGCGAACTTGACGCCGCGGTCTGCACGGT
MmpD_DH_FOR_pOPINF	AAGTTCTGTTTCAGGGCCCGCGCCTGCAACGGCATGAC
MmpD_DH_REV_pOPINF	ATGGTCTAGAAAGCTTTATTATTGCTCTTGTTGGACTGGGGC
MupB_FOR_pOPINF	AAGTTCCTGTTTCAGGGCCCGATGAGAACTGCCTATTTGGCCTCC
MupB_REV_pOPINF	ATGGTCTAGAAAGCTTTATCAGGCATCACTCACCCGTTG

[1] J. Hothersall, J. Wu, A. S. Rahman, J. A. Shields, J. Haddock, N. Johnson, S. M. Cooper, E. R. Stephens, R. J. Cox, J. Crosby, C. L. Willis, T. J. Simpson, C. M. Thomas, *J. Biol. Chem.* **2007**, *282*, 15451-15461.

[2] A. B. Pangborn, M. A. Giardello, R. H. Grubbs, R. K. Rosen, F. J. Timmers, *Organometallics* **1996**, *15*, 1518-1520.

[3] W. C. Still, M. Kahn, A. Mitra, *J. Org. Chem.* **1978**, *43*, 2923-2925.

[4] S. A. Snyder, D. S. Treitler, A. P. Brucks, W. Sattler, *J. Am. Chem. Soc.* **2011**, *133*, 15898-15901.

[5] J. R. Huckins, J. de Vicente, S. D. Rychnovsky, *Org. Lett.* **2007**, *9*, 4757-4760.

[6] H. Ishiwata, T. Nemoto, M. Ojika, K. Yamada, *J. Org. Chem.* **1994**, *59*, 4710-4711.

[7] D. M. Withall, S. W. Haynes, G. L. Challis, *J. Am. Chem. Soc.* **2015**, *137*, 7889-7897.

[8] K. Takizawa, C. Tang, C. J. Hawker, *J. Am. Chem. Soc.* **2008**, *130*, 1718-1726.

[9] K. S. Rao, S. Ghosh, *Synthesis* **2013**, *45*, 2745-2751.

[10] P. D. Walker, C. Williams, A. N. M. Weir, L. Y. Wang, J. Crosby, P. R. Race, T. J. Simpson, C. L. Willis, M. P. Crump, *Angew. Chem. Int. Ed.* **2019**, *58*, 12446-12450.

[11] V. Agarwal, S. Diethelm, L. Ray, N. Garg, T. Awakawa, P. C. Dorrestein, B. S. Moore, *Org. Lett.* **2015**, *17*, 4452-4455.

[12] a A. S. Haines, X. Dong, Z. Song, R. Farmer, C. Williams, J. Hothersall, E. Płoskoń, P. Wattana-Amorn, E. R. Stephens, E. Yamada, R. Gurney, Y. Takebayashi, J. Masschelein, R. J. Cox, R. Lavigne, C. L. Willis, T. J. Simpson, J. Crosby, P. J. Winn, C. M. Thomas, M. P. Crump, *Nat. Chem. Biol.* **2013**, *9*, 685-692; b V. Agarwal, S. Diethelm, L. Ray, N. Garg, T. Awakawa, P. C. Dorrestein, B. S. Moore, *Org. Lett.* **2015**, *17*, 4452-4455.

[13] S. Tayyab, S. Qamar, M. Islam, *Biochem. Educ.* **1991**, *19*, 149-152.

[14] P. D. Walker, M. T. Rowe, A. J. Winter, A. N. M. Weir, N. Akter, L. Y. Wang, P. R. Race, C. Williams, Z. S. Song, T. J. Simpson, C. L. Willis, M. P. Crump, *ACS Chem. Biol.* **2020**, *15*, 494-503.

[15] R. C. Edgar, *Nuc. Acids Res.* **2004**, *32*, 1792-1797.

[16] S. Kumar, G. Stecher, M. Li, C. Knyaz, K. Tamura, *Mol. Biol. Evol.* **2018**, *35*, 1547-1549.

[17] A. Roy, A. Kucukural, Y. Zhang, *Nat. Protocols* **2010**, *5*, 725-738.

[18] C. Notredame, D. G. Higgins, J. Heringa, *J. Mol. Biol.* **2000**, *302*, 205-217.

[19] aU. Sundermann, K. Bravo-Rodriguez, S. Klopries, S. Kushnir, H. Gomez, E. Sanchez-Garcia, F. Schulz, *ACS Chem. Biol.* **2013**, *8*, 443-450; bF. T. Wong, X. Jin, Mathews, II, D. E. Cane, C. Khosla, *Biochemistry* **2011**, *50*, 6539-6548.

[20] B. Zhang, Z. R. Xu, Q. H. Teng, G. H. Pan, M. Ma, B. Shen, *Angew. Chem. Int. Ed.* **2017**, *56*, 7247-7251.

[21] L. A. Kelley, S. Mezulis, C. M. Yates, M. N. Wass, M. J. E. Sternberg, *Nat. Protoc.* **2015**, *10*, 845-858.

[22] J. Jumper, R. Evans, A. Pritzel, T. Green, M. Figurnov, O. Ronneberger, K. Tunyasuvunakool, R. Bates, A. Zidek, A. Potapenko, A. Bridgland, C. Meyer, S. A. A. Kohl, A. J. Ballard, A. Cowie, B. Romera-Paredes, S. Nikolov, R. Jain, J. Adler, T. Back, S. Petersen, D. Reiman, E. Clancy, M. Zielinski, M. Steinegger, M. Pacholska, T. Berghammer, S. Bodenstein, D. Silver, O. Vinyals, A. W. Senior, K. Kavukcuoglu, P. Kohli, D. Hassabis, *Nature* **2021**, *596*, 583-+.

[23] A. Keatinge-Clay, *J. Mol. Biol.* **2008**, *384*, 941-953.
